# Magnetic, Photo- and Electroluminescent: Multifunctional Ionic Tb Complexes

**DOI:** 10.1021/acs.inorgchem.1c01875

**Published:** 2021-11-16

**Authors:** Guillaume Bousrez, Olivier Renier, Veronica Paterlini, Volodymyr Smetana, Anja-Verena Mudring

**Affiliations:** Department of Materials and Environmental Chemistry, Stockholm University, Svante Arrhenius väg 16 C, 10691 Stockholm, Sweden

## Abstract

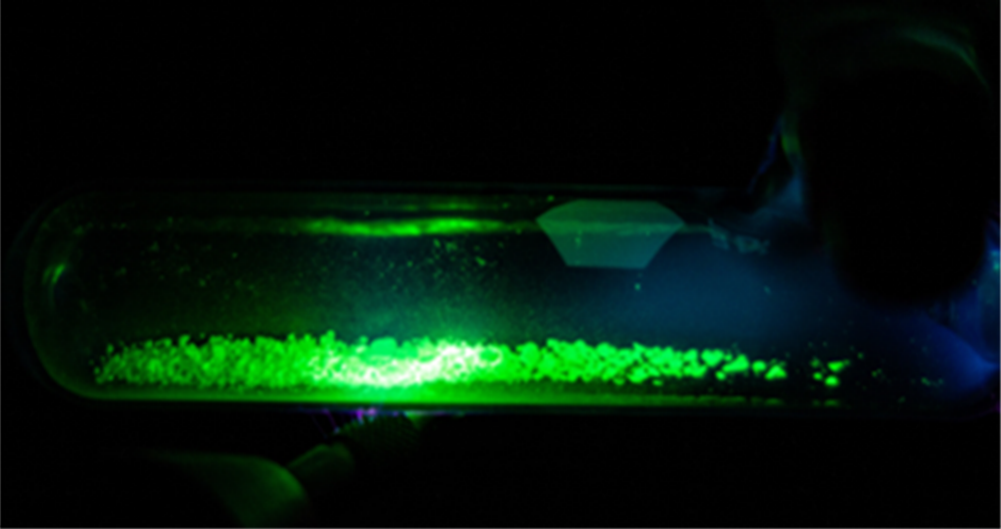

In the search for new multifunctional materials, particularly for application in solid-state lighting, a set of terbium salicylato (Sal) complexes of general composition [Cat][Tb(Sal)_4_] with the commonly ionic liquid-forming (IL) cations [Cat] = (2-hydroxyethyl)trimethylammonium (choline) (Chol^+^), diallyldimethylammonium (DADMA^+^), 1-ethyl-3-methylimidazolium (C_2_C_1_Im^+^), 1-butyl-3-methylimidazolium (C_4_C_1_Im^+^), 1-ethyl-3-vinylimidazolium (C_2_Vim^+^), and tetrabutylphosphonium (P_4444_^+^) were synthesized. All Tb compounds exhibit strong green photoluminescence of high color purity by energy transfer from the ligand in comparison with what the analogous La compounds show, and quantum yields can reach up to 63% upon ligand excitation. When excited with an HF generator, the compounds show strong green electroluminescence with the same features of mission. The findings promise a high potential of application as emitter materials in solid-state lighting. As an additional feature, the Tb compounds show a strong response to applied external fields, rendering them multifunctional materials.

## Abstract 2

Terbium-containing organic-inorganic hybrid materials such as ionic complexes allow for unique property combinations that can open up new applications

## Introduction

Out of societal and environmental concerns, reducing energy consumption is becoming more and more important. Currently, about 25% of the energy consumed worldwide is for lighting applications.^1,2^ Thus, a tremendous contribution to energy saving could be made by more energy efficient lighting devices. Compact fluorescent lamps (CFLs), light emitting diodes (LEDs), and organic light emitting diodes (OLEDs) constitute the current alternatives to non-energy efficient incandescent lamps. Frequently, these devices incorporate rare earth (RE) phosphors as essential light-converting materials. RE ions, in particular, Eu^3+^, Tb^3+^, and Tm^3+^, are widely used due to their efficient emission and high color purity (red, green, and blue, respectively), resulting from the shielding of the *f*-electrons by the outer 5*s* and 5*p* levels. Unfortunately, owing to the parity forbidden *f*-*f* transitions, they also suffer from poor absorption. The incorporation of a sensitizer that can efficiently absorb light and transfer energy can help circumvent this issue. As sensitizers, different RE ions, such as Ce^3+^, broadband semiconductors,^3,4^ organic ligands, and dyes can be used.^5−13^ However, the photostability of organic fluorophores is often quite poor^14^ but can be drastically improved by their incorporation into ionic liquids (ILs).^15,16^ Since the first observation,^9,17−19^ numerous strongly luminescent ILs could be realized based on this design concept.^8,20−22^ The addition of transition metals or *f* elements that are optically active to ILs is also an excellent pathway to luminescent organic-inorganic hybrid materials.^9,23−27^ Ionic transition metal complexes (iTMC) have garnered interest for application in a new post-OLED lighting technology, namely, for light-emitting electrochemical cells (LECs).^28^ Albeit significant recent progress has been made on stable and efficient iTMCs,^29^ enhancing the light-emitting properties of these soft optical materials has always been a challenge.^30−33^ IL cations can act as an antenna and enhance the emission when combined with the Eu^3+^ ion in an imidazolium-based system.^34^ However, the IL cation is not in close proximity to the metal center. Consequently, an improved design approach is to complex the RE metal center with a ligand that allows for efficient RE emission sensitization. By designing the IL in such a way that there is a limited number of high frequency oscillators present in the molecule (such as O–H, N–H, or C–H bonds), it will be possible to enhance the efficiency of photoemission.^35^ RE(III) benzoate complexes have already been studied for their optical properties in their solid state.^36,37^ In this context, the salicylato ligand appears to be quite promising. Salicylic acid and sodium salicylate are cheap, readily available, and nontoxic. The latter has shown a high quantum yield under a large range of excitation wavelengths.^38^ In addition, highly luminescent and color-tunable salicylate ILs have been studied by us previously.^39^ Terbium(III) was chosen as the emitting ion, because it shows in its compound reliable emission of green light in high color purity. Green light emission is of interest in various applications such as signaling (for example, to designate the safety exits), security tags, and many more. With respects to LEC technology, recently, significant progress could be made with respect to green light emitting LECs.^40−42^ However, the iTMCs used in these devices are based on Ir(III) which is considerably more expensive than Tb(III).

In the context of multifunctional materials, the Tb^3+^ ion features a high magnetic moment. This allows combining light emission with a response to a magnetic field, which is an interesting properties combination in terms of stimuli responsive materials.^43^

Consequently, the combination of terbium(III)salicylato anions with cations that are known to support IL formation such as (2-hydroxyethyl)trimethylammonium (choline) (Chol^+^), diallyldimethylammonium (DADMA^+^), 1-ethyl-3-methylimidazolium (C_2_C_1_Im^+^), 1-butyl-3-methylimidazolium (C_4_C_1_Im^+^), 1-ethyl-3-vinylimidazolium (C_2_Vim^+^), and tetrabutylphosphonium (P_4444_^+^) was investigated. DADMA^+^ and C_2_Vim^+^ are polymerizable cations, which allow for the generation of ionic polymers which can be relevant for device application.^13,44^ To verify energy transfer from the salicylate ligand to the terbium center, we have also synthesized the isostructural complexes with lanthanum, a RE cation that shows no light emission itself.

## Experimental Section

1-Vinylimidazole (99%) was purchased from Alfa Aesar (Kandel, Germany) and distilled under reduced pressure at 80 °C prior to use. Tributylphosphine (97%), diallyldimethylammonium chloride (65% solution in water), 1-bromoethane, 1-chlorobutane, and 1-methylimidazole were purchased from Sigma-Aldrich (Steinheim, Germany). 1-Methylimidazole was distilled over KOH before use. Sodium salicylate (99%) and lanthanum(III)-chloride heptahydrate were purchased from ABCR (Kalrsruhe, Germany) and used as received. Terbium (III,IV) oxide (99.99%) was purchased from SmartElements (Vienna, Austria). Choline chloride (97%) and ethanol (99.5%) were purchased from Acros Organics (Geel, Belgium).

### Synthesis of Imidazolium/Ammonium/Phosphonium Salicylate Derivatives

In a flask, the corresponding imidazolium/ammonium/phosphonium chloride ([Cat]Cl) (1.0 equiv) and sodium salicylate (Na[Sal]) (1.0 equiv) were combined in anhydrous ethanol (5 mL). The precipitated NaCl was filtered off and washed with ethanol. The filtrate was concentrated under reduced pressure.

#### 1-Ethyl-3-methylimidazolium Salicylate [C_2_C_1_Im][Sal]

Colorless oil. ^1^H NMR (400 MHz, DMSO-*d*_6_): 1.39 (t, *J*_H-H_ = 7.2 Hz, 3H), 3.85 (s, 3H), 4.19 (q, *J*_H-H_ = 14.8 Hz, *J*_H-H_ = 7.2 Hz, 2H), 6.57–6.63 (m, 2H), 7.13 (t, *J*_H-H_ = 7.6 Hz, 1H), 7.68 (d, *J*_H-H_ = 7.6 Hz, 1H), 7.72 (s, 1H), 7.81 (s, 1H), 9.35 (s, 1H). υ_max_ (cm^–1^): 3387, 3152, 3103, 2991, 1627, 1573, 1485, 1455, 1384, 1329, 1292, 1254, 1221, 1168, 1139, 1087, 1027, 957, 857, 809, 762, 706, 666, 648, 621, 566, 539, 451, 432, 413.

#### 1-Butyl-3-methylimidazolium Salicylate [C_4_C_1_Im][Sal]

Colorless oil. ^1^H NMR (400 MHz, DMSO-*d*_6_): 0.85 (t, *J*_H-H_ = 7.2 Hz, 3H), 1.17–1.24 (m, 2H), 1.70–1.75 (m, 2H), 3.86 (s, 3H), 4.16 (t, *J*_H-H_ = 6.8 Hz, 2H), 6.57–6.63 (m, 2H), 7.13 (t, *J*_H-H_ = 7.6 Hz, 1H), 7.69 (d, *J*_H-H_ = 7.6 Hz, 1H), 7.74 (s, 1H), 7.81 (s, 1H), 9.39 (s, 1H). υ_max_ (cm^–1^): 3394, 3148, 3095, 2961, 2935, 2874, 1627, 1572, 1485, 1455, 1383, 1328, 1291, 1254, 1167, 1139, 1087, 1027, 950, 857, 809, 759, 705, 666, 621, 565, 538, 451.

#### 1-Ethyl-3-vinylimidazolium Salicylate [C_2_Vim][Sal]

White solid. ^1^H NMR (400 MHz, DMSO-*d*_6_): 1.44 (t, *J*_H-H_ = 6.8 Hz, 3H), 4.24 (q, *J*_H-H_ = 14.4 Hz, *J*_H-H_ = 7.2 Hz, 2H), 5.41 (d, *J*_H-H_ = 9.6 Hz, 1H), 5.98 (d, *J*_H-H_ = 15.6 Hz, 1H), 6.57–6.63 (m, 2H), 7.13 (t, *J*_H-H_ = 7.6 Hz, 1H), 7.32 (dd, *J*_H-H_ = 15.6 Hz, *J*_H-H_ = 8.8 Hz, 1H), 7.67 (d, *J*_H-H_ = 7.6 Hz, 1H), 7.97 (s, 1H), 8.23 (s, 1H), 9.67 (s, 1H), 16.11 (s, 1H). υ_max_ (cm^–1^): 3131, 3054, 2990, 2990, 2979, 1649, 1649, 1649, 1649, 1579, 1545, 1483, 1458, 1374, 1296, 1247, 1185, 1168, 1141, 1083, 1048, 1029, 978, 926, 857, 809, 783, 764, 741, 699, 669, 634, 618, 595, 567, 537, 466.

#### Choline Salicylate [Chol][Sal]

Colorless oil. ^1^H NMR (400 MHz, DMSO-*d*_6_): 1.03 (s, 9H), 1.31–1.33 (m, 4H), 1.75 (s, 2H), 3.63 (s, 1H), 4.47–4.53 (m, 2H), 5.03 (dd, *J*_H-H_ = 7.2 Hz, *J*_H-H_ = 6.8 Hz, 1H), 5.56 (d, *J*_H-H_ = 7.6 Hz, 1H), 14.17 (s, 1H). υ_max_ (cm^–1^): 3231, 3031, 2856, 1627, 1577, 1483, 1455, 1381, 1326, 1292, 1253, 1138, 1085, 1056, 1027, 1004, 951, 857, 808, 762, 706, 665, 566, 538, 451.

#### Diallyldimethylammonium Salicylate [DADMA][Sal]

Colorless oil. ^1^H NMR (400 MHz, DMSO-*d*_6_): 0.48 (s, 6H), 1.46–1.48 (m, 4H), 3.09 (t, *J*_H-H_ = 11.6 Hz, 4H), 3.53 (h, *J*_H-H_ = 24.4 Hz, *J*_H-H_ = 15.2 Hz, *J*_H-H_ = 7.6 Hz, 2H), 4.08–4.14 (m, 2H), 4.64 (t, *J*_H-H_ = 7.6 Hz, 1H), 5.19 (d, *J*_H-H_ = 7.6 Hz, 1H). υ_max_ (cm^–1^): 3385, 3028, 2985, 1627, 1583, 1484, 1455, 1422, 1382, 1324, 1289, 1255, 1139, 1086, 993, 952, 871, 857, 809, 762, 706, 666, 606, 566, 538, 451.

#### Tetrabutylphosphonium Salicylate [P_4444_][Sal]

Yellow oil. ^1^H NMR (400 MHz, DMSO-*d*_6_): 0.91 (t, *J*_H-H_ = 6.8 Hz, 12H), 1.37–1.48 (m, 16H), 2.15–2.22 (m, 8H), 6.58 (dd, *J*_H-H_ = 14.4 Hz, *J*_H-H_ = 7.2 Hz, 2H), 7.12 (dd, *J*_H-H_ = 8.8 Hz, *J*_H-H_ = 8.8 Hz, 1H), 7.66 (d, *J*_H-H_ = 9.2 Hz, 1H). ^31^P NMR (162 MHz, DMSO-*d*_6_): 33.9. υ_max_ (cm^–1^): 2959, 2932, 2872, 1632, 1588, 1486, 1457, 1387, 1332, 1302, 1255, 1224, 1188, 1138, 1095, 1052, 1027, 1002, 966, 907, 860, 810, 758, 705, 666, 566, 537, 454.

### Synthesis of the Lanthanide(III) Salicylates

Rare earth (III) chloride hydrate (RECl_3_·*x*H_2_O (RE = La or Tb; *x* = 6 or 7)) (10 mmol, 1.0 equiv) and sodium salicylate (Na[Sal]) (30 mmol, 3.0 equiv) were combined in acetone (10 mL). NaCl forms a precipitate that is then removed by filtration. After washing the remainder with acetone, the solvent was removed under reduced pressure.

#### Lanthanum Salicylate La(Sal)_3_·H_2_O

White solid. ^1^H NMR (400 MHz, DMSO-*d*_6_): 6.70–6.74 (m, 2H), 7.26 (t, *J*_H-H_ = 7.2 Hz, 1H), 7.75 (d, *J*_H-H_ = 7.2 Hz, 1H). υ_max_ (cm^–1^): 3325, 3066, 1667, 1607, 1594, 1560, 1546, 1508, 1480, 1464, 1438, 1417, 1404, 1381, 1308, 1240,1220, 1162, 1148, 1106, 1032, 945, 902, 880, 848, 802, 749, 701, 660, 569, 551, 528, 477, 465, 417.

#### Terbium Salicylate Monohydrate Tb(Sal)_3_·H_2_O

Light brown solid. υ_max_ (cm^–1^): 3054, 1667, 1622, 1593, 1581, 1548, 1482, 1460, 1385, 1308, 1241, 1218, 1159, 1145, 1101, 1080, 1031, 953, 878, 834, 804, 751, 701, 661, 568, 528, 472.

### Synthesis of Imidazolium/Ammonium/Phosphonium Tetrakissalicylatolanthanidates [Cat][RE(Sal)_4_^¬^]

The RE(III) salicylate (RE(Sal)_3_·H_2_O (RE = La or Tb)) (1.0 mmol, 1.0 equiv) and the corresponding imidazolium/ammonium/phosphonium salicylate ([Cat][Sal]) (1.5 mmol, 1.5 equiv) were dissolved in anhydrous ethanol (5 mL). The precipitate, later identified as [Cat][RE(Sal)_4_], was separated by filtration and washed with ethanol. Finally, the compound was dried at 80 °C for at least 6 h. The final yield is between 80% and 85%.

#### 1-Ethyl-3-methylimidazolium Tetrakissalicylatolanthanate [C_2_C_1_Im][La(Sal)_4_]

White solid. ^1^H NMR (400 MHz, DMSO-*d*_6_): 1.40–1.42 (m, 3H), 3.84 (s, 3H), 4.17–4.19 (m, 2H), 6.68 (bs, 8H), 7.21 (bs, 4H), 7.70 (s, 1H). υ_max_ (cm^–1^): 3152, 3050, 1626, 1593, 1563, 1482, 1458, 1385, 1346, 1309, 1249, 1223, 1162, 1143, 1092, 1030, 957, 885, 864, 816, 756, 703, 663, 620, 588, 570, 534, 457, 431. ESI TOF *m*/*z* (positive mode) 111.1342 (calculated *m*/*z* = 111.0917). ESI TOF *m*/*z* (negative mode) 687.0403 (calculated *m*/*z* = 687.0018).

#### 1-Ethyl-3-methylimidazolium Tetrakissalicylatoterbate [C_2_C_1_Im][Tb(Sal)_4_]

Light brown solid. υ_max_ (cm^–1^): 2993, 1634, 1577, 1481, 1461, 1388, 1341, 1309, 1246, 1217, 1160, 1144, 1090, 1029, 957, 866, 819, 806, 752, 703, 662, 647, 621, 597, 574, 534, 458. ESI TOF *m*/*z* (positive mode) 111.1342 (calculated *m*/*z* = 111.0917). ESI TOF *m*/*z* (negative mode) 707.0661 (calculated *m*/*z* = 707.0208).

#### 1-Butyl-3-methylimidazolium Tetrakissalicylatolanthanate [C_4_C_1_Im][La(Sal)_4_]

White solid. ^1^H NMR (400 MHz, DMSO-*d*_6_): 0.89 (t, *J*_H-H_ = 7.2 Hz, 3H), 1.25 (h, *J*_H-H_ = 22.0 Hz, *J*_H-H_ = 14.4 Hz, *J*_H-H_ = 7.2 Hz, 2H), 1.75 (p, *J*_H-H_ = 14.8 Hz, *J*_H-H_ = 7.2 Hz, 2H), 3.84 (s, 3H), 4.15 (t, *J*_H-H_ = 7.2 Hz, 2H), 6.65–6.70 (m, 8H), 7.20 (d, *J*_H-H_ = 7.6 Hz, 2H), 7.22 (d, *J*_H-H_ = 7.2 Hz, 2H), 7.71–7.73 (m, 5H), 7.77 (s, 1H), 9.15 (s, 1H), 14.88 (bs, 3H). υ_max_ (cm^–1^): 3152, 3087, 2969, 2937, 2861, 1675, 1591, 1568, 1481, 1459, 1385, 1337, 1306, 1251, 1223, 1192, 1174, 1156, 1141, 1092, 1029, 976, 957, 863, 843, 816, 806, 760, 703, 663, 638, 622, 600, 569, 536, 460, 430. ESI TOF *m*/*z* (positive mode) 139.1635 (calculated *m*/*z* = 139.1230). ESI TOF *m*/*z* (negative mode) 687.0403 (calculated *m*/*z* = 687.0018).

#### 1-Butyl-3-methylimidazolium Tetrakissalicylatoterbate [C_4_C_1_Im][Tb(Sal)_4_]

Light brown solid. υ_max_ (cm^–1^): 3053, 2959, 2871, 1624, 1592, 1556, 1481, 1458, 1382, 1308, 1246, 1220, 1157, 1142, 1029, 955, 886, 866, 818, 753, 703, 662, 620, 598, 570, 551, 533, 463. ESI TOF *m*/*z* (positive mode) 139.1635 (calculated *m*/*z* = 139.1230). ESI TOF *m*/*z* (negative mode) 707.0661 (calculated *m*/*z* = 707.0208).

#### 1-Ethyl-3-vinylimidazolium Tetrakissalicylatolanthanate [C_2_Vim][La(Sal)_4_]

White solid. ^1^H NMR (400 MHz, DMSO-*d*_6_): 1.43 (t, *J*_H-H_ = 7.2 Hz, 3H), 4.22 (q, *J*_H-H_ = 14.4 Hz, *J*_H-H_ = 6.8 Hz, 2H), 5.41 (d, *J*_H-H_ = 8.4 Hz, 1H), 5.95 (d, *J*_H-H_ = 15.6 Hz, 1H), 6.64 (bs, 8H), 7.16 (bs, 4H), 7.30 (dd, *J*_H-H_ = 15.6 Hz, *J*_H-H_ = 8.8 Hz, 1H), 7.72 (bs, 4H), 7.94 (s, 1H), 8.20 (s, 1H), 9.55 (s, 1H). υ_max_ (cm^–1^): 3436, 3162, 3141, 3048, 1624, 1597, 1546, 1494, 1456, 1386, 1335, 1310, 1251, 1161, 1140, 1107, 1031, 945, 917, 886, 863, 829, 817, 805, 756, 736, 702, 662, 615, 586, 575, 553, 534, 458, 432. ESI TOF *m*/*z* (positive mode) 123.1323 (calculated *m*/*z* = 123.0917). ESI TOF *m*/*z* (negative mode) 687.0403 (calculated *m*/*z* = 687.0018).

#### 1-Ethyl-3-vinylimidazolium Tetrakissalicylatoterbate [C_2_Vim][Tb(Sal)_4_]

Light brown solid. υ_max_ (cm^–1^): 3054, 1623, 1593, 1548, 1480, 1459, 1381, 1309, 1244, 1220, 1158, 1143, 1098, 1030, 952, 917, 886, 865, 817, 805, 753, 703, 662, 595, 571, 553, 532, 463. ESI TOF *m*/*z* (positive mode) 123.1323 (calculated *m*/*z* = 123.0917). ESI TOF *m*/*z* (negative mode) 707.0661 (calculated *m*/*z* = 707.0208).

#### Choline Tetrakissalicylatolanthanate [Chol][La(Sal)_4_]

White solid. ^1^H NMR (400 MHz, DMSO-*d*_6_): 3.10 (s, 9H), 3.39 (t, *J*_H-H_ = 4.8 Hz, 2H), 3.83 (bs, 2H), 5.36 (t, *J*_H-H_ = 4.4 Hz, 1H), 6.62 (bs, 8H), 7.15 (bs, 4H), 7.68 (bs, 4H). υ_max_ (cm^–1^): 1593, 1561, 1529, 1480, 1457, 1380, 1308, 1246, 1145, 1090, 1031, 995, 951, 878, 865, 820, 805, 755, 704, 661, 592, 569, 536, 493, 459. ESI TOF *m*/*z* (positive mode) 104.1485 (calculated *m*/*z* = 104.1075). ESI TOF *m*/*z* (negative mode) 687.0403 (calculated *m*/*z* = 687.0018).

#### Choline Tetrakissalicylatoterbate [Chol][Tb(Sal)_4_]

Light brown solid. υ_max_ (cm^–1^): 3052, 1624, 1593, 1560, 1481, 1460, 1385, 1341, 1308, 1245, 1145, 1087, 1031, 999, 952, 886, 866, 819, 755, 704, 663, 572, 551, 534, 462. ESI TOF *m*/*z* (positive mode) 104.1485 (calculated *m*/*z* = 104.1075). ESI TOF *m*/*z* (negative mode) 707.0661 (calculated *m*/*z* = 707.0208).

#### Diallyldimethylammonium Tetrakissalicylatolanthanate [DADMA][La(Sal)_4_]

White solid. ^1^H NMR (400 MHz, DMSO-*d*_6_): 2.95 (s, 6H), 3.91 (d, *J*_H-H_ = 6.8 Hz, 4H), 5.60–5.64 (m, 4H), 6.06 (h, *J*_H-H_ = 24.0 Hz, *J*_H-H_ = 16.0 Hz, *J*_H-H_ = 7.2 Hz, 2H), 6.63 (bs, 8H), 7.16 (bs, 4H), 7.72 (bs, 4H). υ_max_ (cm^–1^): 3617, 3390, 3046, 1625, 1597, 1559, 1544, 1492, 1457, 1383, 1336, 1309, 1251, 1156, 1138, 1098, 1031, 1006, 991, 958, 887, 863, 832, 818, 805, 755, 701, 661, 586, 575, 552, 535, 457, 431. ESI TOF *m*/*z* (positive mode) 126.1679 (calculated *m*/*z* = 126.1277). ESI TOF *m*/*z* (negative mode) 687.0403 (calculated *m*/*z* = 687.0018).

#### Diallyldimethylammonium Tetrakissalicylatoterbate [DADMA][Tb(Sal)_4_]

Light brown solid. υ_max_ (cm^–1^): 3052, 1625, 1594, 1559, 1482, 1458, 1387, 1308, 1248, 1158, 1143, 1095, 1030, 990, 959, 868, 819, 754, 704, 663, 598, 571, 549, 533, 461. ESI TOF *m*/*z* (positive mode) 126.1679 (calculated *m*/*z* = 126.1277). ESI TOF *m*/*z* (negative mode) 707.0661 (calculated *m*/*z* = 707.0208).

#### Tetrabutylphosphonium Tetrakissalicylatolanthanate [P_4444_][La(Sal)_4_]

Light brown oil. ^1^H NMR (400 MHz, DMSO-*d*_6_): 0.91 (t, *J*_H-H_ = 6.8 Hz, 12H), 1.37–1.46 (m, 16H), 2.14–2.21 (m, 8H), 6.57 (d, *J*_H-H_ = 7.2 Hz, 4H), 6.61 (d, *J*_H-H_ = 8.0 Hz, 4H), 7.13 (t, *J*_H-H_ = 7.2 Hz, 4H), 7.66 (d, *J*_H-H_ = 7.2 Hz, 4H). ^31^P NMR (162 MHz, DMSO-*d*_6_): 34.6. υ_max_ (cm^–1^): 3307, 3248, 2959, 2932, 2872, 1631, 1585, 1486, 1458, 1383, 1346, 1289, 1256, 1226, 1190, 1154, 1138, 1096, 1052, 1027, 1003, 967, 907, 856, 808, 757, 704, 665, 566, 538, 455. ESI TOF *m*/*z* (positive mode) 259.3063 (calculated *m*/*z* = 269.2555). ESI TOF *m*/*z* (negative mode) 707.0661 (calculated *m*/*z* = 707.0208).

#### Tetrabutylphosphonium Tetrakissalicylatoterbate [P_4444_][Tb(Sal)_4_]

Light brown solid. υ_max_ (cm^–1^): 3057, 2962, 2932, 2872, 1625, 1594, 1558, 1483, 1459, 1386, 1308, 1249, 1222, 1157, 1142, 1091, 1046, 1030, 964, 908, 868, 819, 805, 754, 703, 663, 567, 550, 533, 461. ESI TOF *m*/*z* (positive mode) 259.3063 (calculated *m*/*z* = 269.2555). ESI TOF *m*/*z* (negative mode) 707.0661 (calculated *m*/*z* = 707.0208).

## Materials and Methods

Photoluminescence (absorption, fluorescence, and phosphorescence) measurements were carried out on a Fluorolog FL 3-22 spectrometer at room temperature (Horiba JobinYvon, Unterhachingen, Germany), equipped with a double excitation monochromator, a single emission monochromator (HR320), and a R928P PMT detector. For steady state measurements, a continuous xenon lamp (450 W) is used, whereas a pulsed xenon lamp is used for lifetime measurements. To perform low temperature measurements, the solid samples were put in quartz capillaries located in a Dewar filled with liquid nitrogen.

The absorbance was calculated as *A* = log(*I*_0_/*I*_s_), where *I*_0_ and *I*_s_ are the intensities of the synchronous scans of the blank (BaSO_4_) and the sample, respectively. Absolute quantum yield (QY) was measured with a G8 integrating sphere (GMP), using BaSO_4_ as blank.

For electroluminescence measurements, the samples were put in a Schlenk flask under vacuum and were excited with a Tesla generator VP202 High Frequency Tester (Leybold Heraeus, Germany). An optical fiber was used to collect the light emitted from the sample and was inserted into the spectrometer chamber through a hole, directed toward the detector. The electroluminescence was then collected with a standard emission mode.

Thermogravimetric Analysis (TGA) was performed with a TG 449 F3 Jupiter (Netzsch, Selb, Germany). Measurements were carried out in aluminum oxide crucibles with a heating rate of 10 °C/min and nitrogen as purge gas.

Differential scanning calorimetry (DSC) was performed with a computer-controlled PhoenixDSC 204 F1 thermal analyzer (Netzsch, Selb, Germany). A heating rate of 5 °C/min was used for the measurements that are carried out from −60 to 210 °C under a nitrogen atmosphere supplied by a flow at a rate of 40 mL/min. Cold-sealed and punctured aluminum pans were used as sample containers. The samples were first cooled to −60 °C. Given temperatures correspond to the onset.

A Bruker 400 MHz spectrometer equipped with a BBO probe (Bruker, Ettlingen, Germany) was used to collect the ^1^H NMR at room temperature in DMSO-*d*_6_. Chemical shifts are reported in delta (δ) units, expressed in parts per million (ppm). The following abbreviations were used for the observed multiplicities: s (singlet), d (doublet), t (triplet), q (quartet), bs (broad singlet), m (multiplet for unresolved lines). The residual solvent signal (2.50 ppm) was used as a reference for the chemical shifts.

The mass spectrometry experiments were performed on a SYNAPT G2-S HDMS Q-ToF Mass Spectrometer (Waters, Manchester, United Kingdom) with an ESI operated in the positive and negative ion modes. The following parameters were used for the ion source: capillary voltage: 2500 V, extractor: 1.0 V, RF lens: 0.5 V, ion source temperature: 120 °C, and desolvation temperature: 250 °C. Both the cone and desolvation gas was nitrogen and supplied at a rate of 70 L/h and 500 L/h, respectively. Argon was used as a collision gas at a pressure of 2.95 × 10^–4^ mbar. The data reported correspond to low resolution mass spectrometry (LRMS).

The infrared spectroscopy (IR) was conducted with a Bruker Alpha-P ATR-spectrometer (Karlsruhe, Germany) in attenuated total reflection configuration. The data evaluation was carried out with the program OPUS (Bruker, Ettlingen, Germany).

Crystal structure determination: Suitable crystals of [C_2_C_1_Im]_4_[RE_4_(Sal)_16_(H_2_O)_2_] (RE = La, Tb) were mounted on a cryoloop (Hampton Research, Aliso Viejo). A STOE IPDS I single crystal X-ray diffractometer using monochromated Mo Kα X-ray radiation (0.70173 Å) was used in this study. Experimental conditions, and the most important crystallographic data are presented in Table S1. X-Red^45^ was used for data reduction which included Lorentz corrections for background and polarization effects. Crystal shape optimization was done using X-Shape,^46^ followed by absorption correction with X-Red.^45^ The crystal structures were solved by direct methods using SIR92,^47^ and the atoms were refined with SHELXL-97^48^ against *F*^2^ by a full-matrix least-squares procedure. All atoms were anisotropically refined apart from the hydrogens atoms which were constrained to match the atom they are bonded to. Structure factors were taken from International Tables for Crystallography (International Table for Crystallography; PrinceE., Ed.; Kluwer). Diamond^49^ was used to draw the crystal structure.

The magnetic properties were measured on a Physical Properties Measurement System (PPMS) from Quantum design (USA). The Vibrating Sample Magnetometer (VMS) option was used for temperature and field dependence in static (DC) fields at 0.1 T. Polycrystalline samples with an approximate mass of 10 mg were loaded into polypropylene capsules, which were mounted in a brass sample holder.

## Results and Discussion

**Scheme 1:**
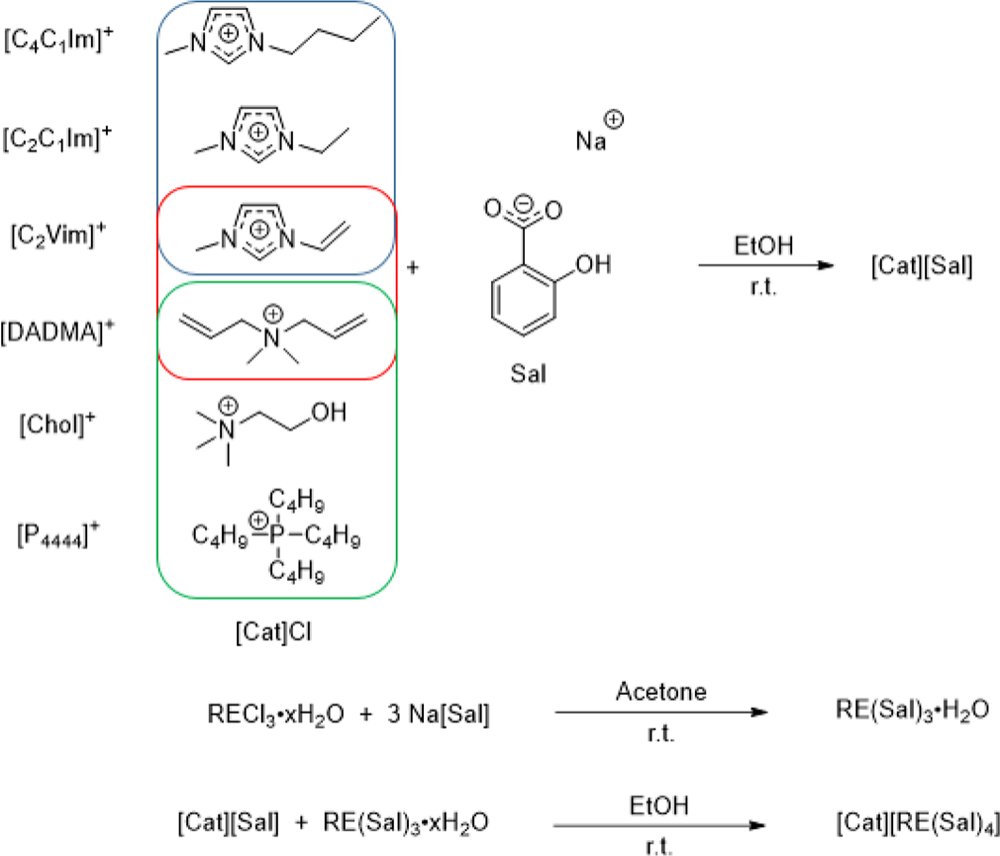
Preparation of [Cat][RE(Sal)_4_] (RE = La or Tb) Blue: aromatic; red: polymerizable; and green: nonaromatic.

The set of ionic lanthanum and terbium salicylate complexes with IL supporting cations were prepared by combining ethanolic solutions of an equimolar amount of RE(III) salicylate (RE(Sal)_3_·H_2_O, RE = La or Tb) and the salicylate IL ([Cat][Sal]). This led to the immediate precipitation of the products ([Cat][RE(Sal)_4_]) as white solids (Scheme 1). The cations were chosen to have different properties, such as strong-to-moderate hydrogen bonding (Chol^+^, C_2_C_1_Im^+^, C_4_C_1_Im^+^, C_2_Vim^+^), non-supporting hydrogen bonding (DADMA^+^, P_4444_^+^), aromatic (C_2_C_1_Im^+^, C_4_C_1_Im^+^, C_2_Vim^+^), nonaromatic (Chol^+^, DADMA^+^, P_4444_^+^), and polymerizable (DADMA^+^, C_2_Vim^+^) (Scheme 1).

### Structural Properties

As expected for compounds with a general formula [Cat][RE(Sal)_4_], electrospray ionization (ESI-MS) mass spectra measured in negative mode show peaks for the tetrakissalicylato anion, [RE(Sal)_4_]^−^, with *m*/*z* = 687.0403 for [La(Sal)_4_]^−^ and *m*/*z*= 707.0661 for [Tb(Sal)_4_]^−^ (Figures S41–44, Supporting Information (SI)).

Albeit all compounds were obtained as white, crystalline solids, the crystallinity was too low to obtain crystals of sufficient quality for single crystal X-ray structure analysis. This most likely comes from the formation of polymeric complex anion structures which notoriously impedes the formation of single crystals for structure analysis. Similar behavior has been observed in the hydrogen-bonded MOFs of the lanthanide dicyanamides upon dehydration.^33^ Indications for the formation of a polymeric structure comes also from the structure analysis of the hemihydrate of [C_2_C_1_Im][RE(Sal)_4_] (RE = La or Tb) for which crystals of sufficient quality for single crystal X-ray structural analysis could be obtained *via* slow hydrolysis under ambient conditions.

**Figure 1:**
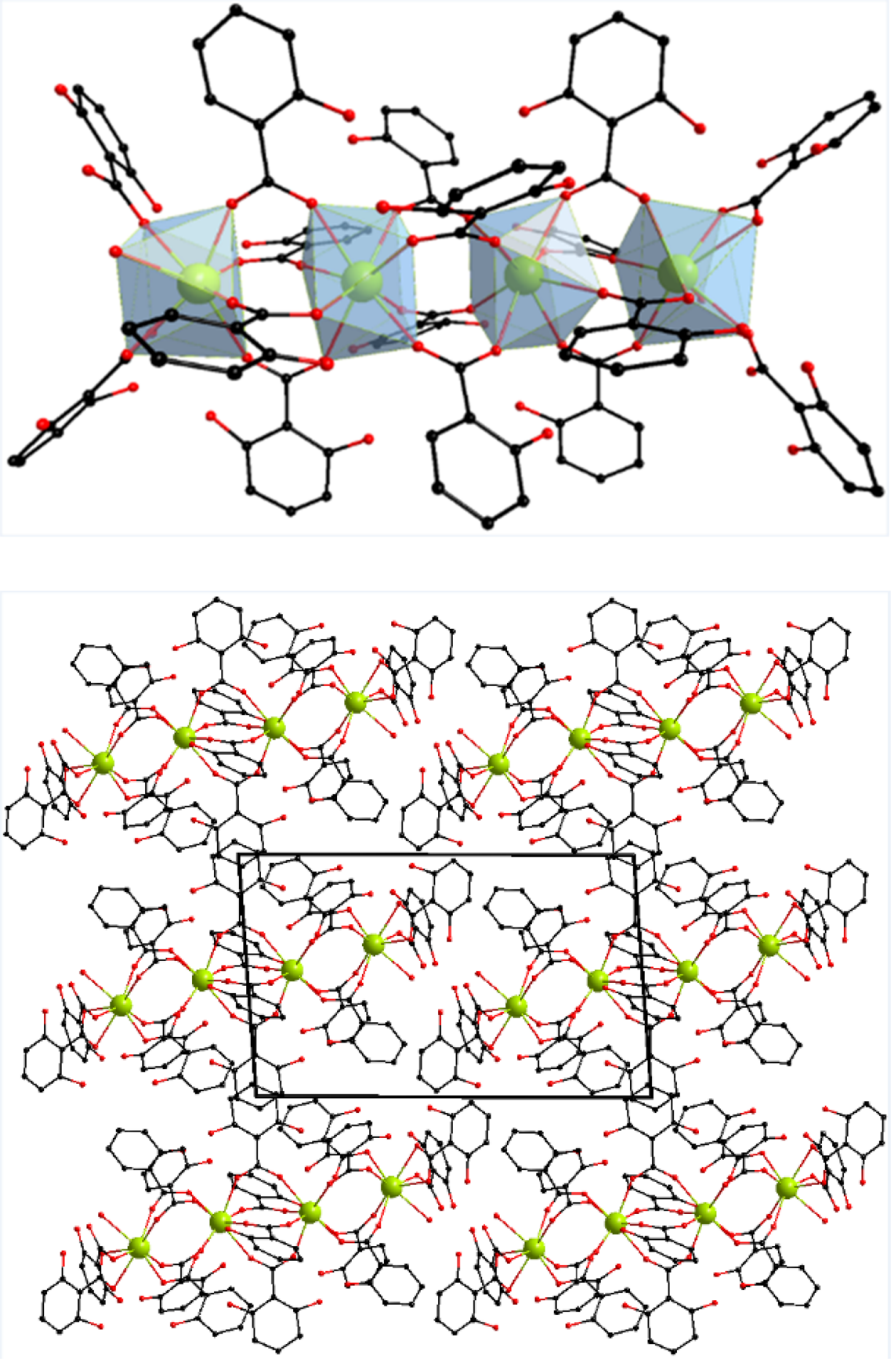
(Top) Anionic unit [La_4_(Sal)_16_(H_2_O)_2_]^4–^ as observed in [C_2_C_1_Im]_4_[RE_4_(Sal)_16_(H_2_O)_2_] (RE = La or Tb) (RE atoms are shown in green, O in red, and C in black). (Bottom) Packing diagram of [C_2_C_1_Im]_4_[La_4_(Sal)_16_(H_2_O)_2_]. View along the crystallographic *c* axis. C_2_C_1_Im^+^ and H atoms are omitted for clarity purposes. La atoms are shown in green, O in red, and C in black.

[C_2_C_1_Im]_4_[RE_4_(Sal)_16_(H_2_O)_2_] (RE = La or Tb) crystallize isotypic in the triclinic space group *P*1̅ (no. 2) with one formula unit in the unit cell. The most distinct structural feature is a linear tetrameric polyanion, [RE_4_(Sal)_16_(H_2_O)_2_]^4–^ (Figure 1, top), consisting of four RE cations connected by 16 salicylate anions. A center of inversion lies between the two middle RE cations. Both crystallographically independent RE ions are coordinated to eight oxygen atoms, forming coordination polyhedra which can be best described as a square antiprism distorted to a different extent. Examining the coordination sphere more closely, the central RE ions are coordinated solely by eight salicylate ligands in a μ_2_-bidentate fashion. The terminal RE positions are coordinated by four μ_2_-bidentate salicylate ligands complemented by one salicylate ligand in μ_1_-bidentate mode, one in monodentate mode, and one water molecule. It should, however, be noted that one of the bridges between the central and peripheral RE atoms is approaching a chelato-bridging mode, which definitely is the reason for a small deviation from linearity in the Ln_4_ chain. The anionic tetramers further link to the neighboring identical units *via* moderate H-bonded bridges, resulting in a polymeric chain along the *c* axis (Figure 1, bottom, and Figure S2 (SI)). These bridges are formed between two monodentate salicylate anions and two water molecules, resulting in an O_4_H_4_ rectangle (see Figure S1 (SI)).

The linear [La_4_(Sal)_16_(H_2_O)_2_]^4–^ tetrameric units are surrounded by the C_2_C_1_Im cations that are located between the neighboring anions along the *a* and *b* axes. Each polyanion exhibits short contacts to eight cations *via* moderate OH···π, CH···π, or π···π stacking between the imidazole and the salicylate aromatic rings (Figure S3 (SI)).

The structure of [C_2_C_1_Im]_4_[La_4_(Sal)_16_(H_2_O)_2_] = 4{[C_2_C_1_Im][La(Sal)_4_]·0.5H_2_O} illustrates that the salicylato ligand has a strong tendency to lead to the formation of polymeric structures. This could explain why the growth of single crystals for structure analysis turned out to be so difficult. For example, the structures of simple salts such as RE(OAc)_3_ (OAc: acetate), RE(OTf)_3_ (OTf: trifluoromethanesulfonate), and RE(NTf_2_)_3_ (NTf_2_: bis(trifluoromethanesulfonyl)amide) are still elusive. Water, as a Lewis base, is apparently able to depolymerize the ^1^_∞_[RE(Sal)_4_] chains, i.e., break them up into shorter [RE_4_(Sal)_16_(H_2_O)_2_]^4–^ fragments with H_2_O coordinating the terminal RE cations. The formation of [C_2_C_1_Im]_4_[La_4_(Sal)_16_(H_2_O)_2_] also clearly shows that the complexes are sensitive to water, which was also identified as a potential source for degradation of OLEDs based on neutral Tb-salicylato complexes.^39^

### Thermal Properties

To assess the thermal properties of the different compounds, thermogravimetric analysis was carried out (Table 1). All the complexes synthesized are anhydrous. Thermogravimetric analysis reveals them not to decompose below 200 °C, with decomposition temperatures ranging from 209 °C for [P_4444_][Tb(Sal)_4_] to 233 °C for [Chol][La(Sal)_4_]. Within error limits (± 5 °C), the corresponding La and Tb compounds have the same decomposition temperatures and follow a multistep decomposition. Except for the compounds with [P_4444_], all compounds decompose before melting. This behavior could be explained based on a polymeric anionic network that prevents melting as it is tightly bound. The decomposition is probably due to the highly Lewis acidic character of the RE^3+^ centers which, at elevated temperatures, leads to the decomposition of the salicylate ligand. This hypothesis is supported by the observation that no strong influence of the organic cation on the decomposition temperature could be made out. In addition, the decomposition point of the ternary salts is close to that of the binary lanthanum and terbium salicylates (*T*_onset_ = 221 °C for La(Sal)_3_ and 238 °C for Tb(Sal)_3_, respectively).

**Table 1:** Decomposition Temperatures and Water Content of the Prepared Samples Derived from Thermogravimetric Analysis^a^

entry	compound	decomposition temperature (*T*_5% onset_, °C)
1A	[C_2_C_1_Im][La(Sal)_4_]	219
1B	[C_2_C_1_Im][Tb(Sal)_4_]	220
2A	[C_4_C_1_Im][La(Sal)_4_]	212
2B	[C_4_C_1_Im][Tb(Sal)_4_]	210
3A	[C_2_Vim][La(Sal)_4_]	217
3B	[C_2_Vim][Tb(Sal)_4_]	220
4A	[DADMA][La(Sal)_4_]	227
4B	[DADMA][Tb(Sal)_4_]	222
5A	[Chol][La(Sal)_4_]	233
5B	[Chol][Tb(Sal)_4_]	228
6A	[P_4444_][La(Sal)_4_]	217
6B	[P_4444_][Tb(Sal)_4_]	209
7A	La(Sal)_3_·H_2_O	84 (-H_2_O)/221
7B	Tb(Sal)_3_·H_2_O	86 (-H_2_O)/238

a The water loss was calculated from the mass loss between 60 and 130 °C.

To put in context, we compared the 1-alkyl-3-methylimidazolium terbium compounds with some reported in the literature.^33,50^ The compounds [C_*n*_C_1_Im][Tb(NO_3_)_3_]^50^ (*n* = 2, 4, 6, or 8) present a better stability because they start their decomposition between 250 and 270 °C. They also show that the longer the chain length, the more stable the compound is, which is different compared to the salicylate equivalents. And in the case of [C_2_C_1_Im][Tb(DCA)_3_(H_2_O)_4_] (DCA = dicyanamide),^33^ the thermal decomposition starts very low (below 150 °C) which is described as the loss of water molecules coordinated to the metal center. Then the compound is reported as stable below 300 °C. This comparison tends to show that the polymeric structure of the salicylate anion has a role in the thermal behavior of the compound.

Differential scanning calorimetry for the phosphonium complex, [P_4444_][Tb(Sal)_4_], reveals, compared to the other representatives of the series of complex compounds, a more complex behavior. Upon heating the sample from room temperature, a solid-solid transition occurs at 46.3 °C; a higher order mesophase is adopted shortly before melting sets in at 162.4 °C. Upon cooling with the same thermal ramp, no reverse processes are observed and the materials stays a supercooled liquid even below room temperature (Figure S59 (SI)).

### Optical Properties

**Figure 2:**
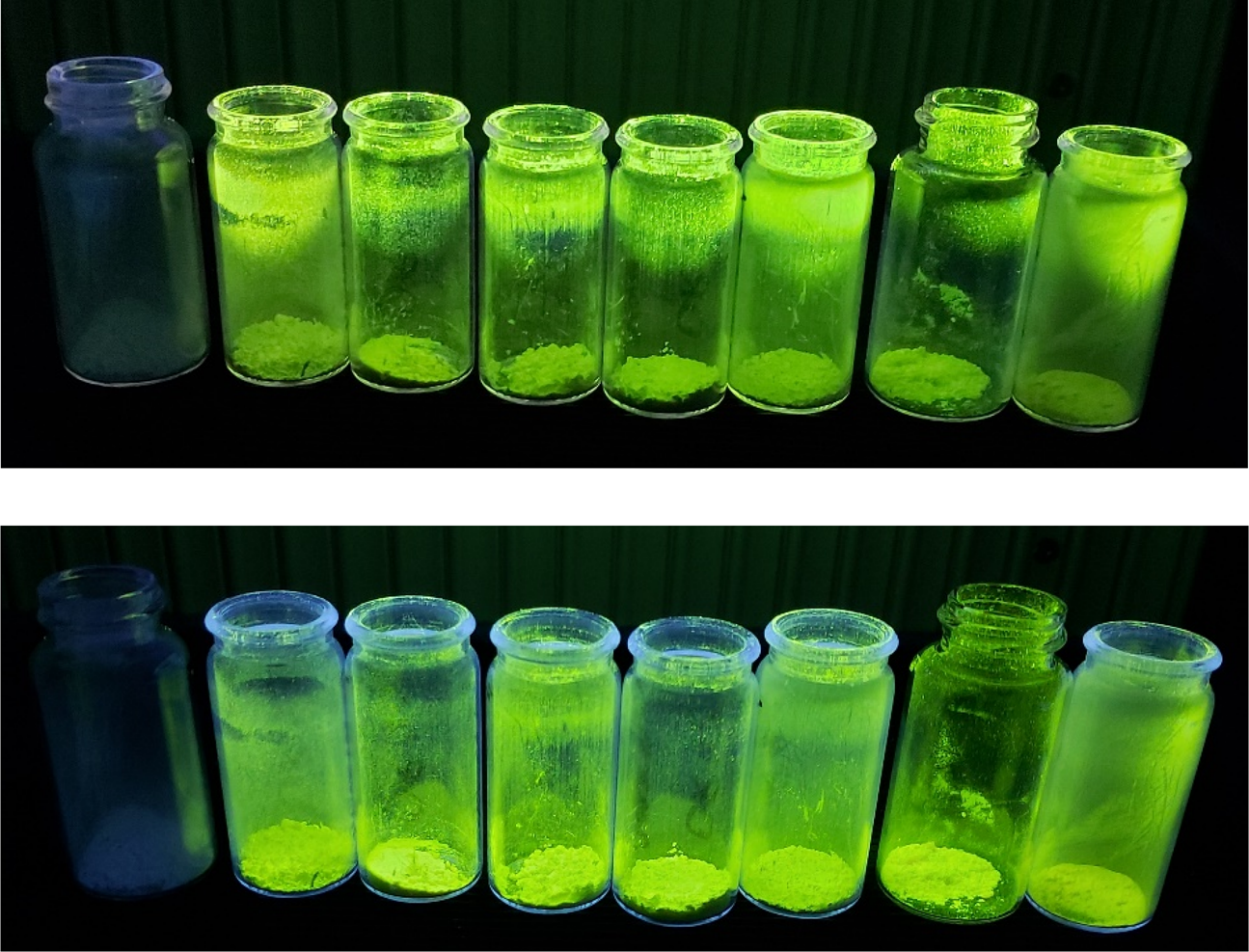
Salicylate compounds under UV lamp excitation at 254 nm (top) and 366 nm (bottom). Samples order in both pictures, from left to right: La(Sal)_3_·H_2_O, [C_2_C_1_Im][Tb(Sal)_4_], [C_4_C_1_Im][Tb(Sal)_4_], [C_2_Vim][Tb(Sal)_4_], [DADMA][Tb(Sal)_4_], [Chol][Tb(Sal)_4_], [P_4444_][Tb(Sal)_4_], Tb(Sal)_3_·H_2_O.

Already under illumination by a conventional hand-held UV lamp (λ_ex_ = 254 and/or 366 nm), the as-prepared terbium(III) compounds exhibit bright green fluorescence easily observable by the naked eye, while the lanthanum ones only show a weak luminescence attributable to the salicylate ligand (Figure 2).

A detailed photophysical characterization was performed in order to establish the optical properties of the studied compounds thoroughly. First, the optical properties of the lanthanum compounds were studied in order to obtain information on the optical properties of the salicylate ligand and, potentially, of the IL cation, in the ionic materials. Then the terbium salts were studied.

**Figure 3:**
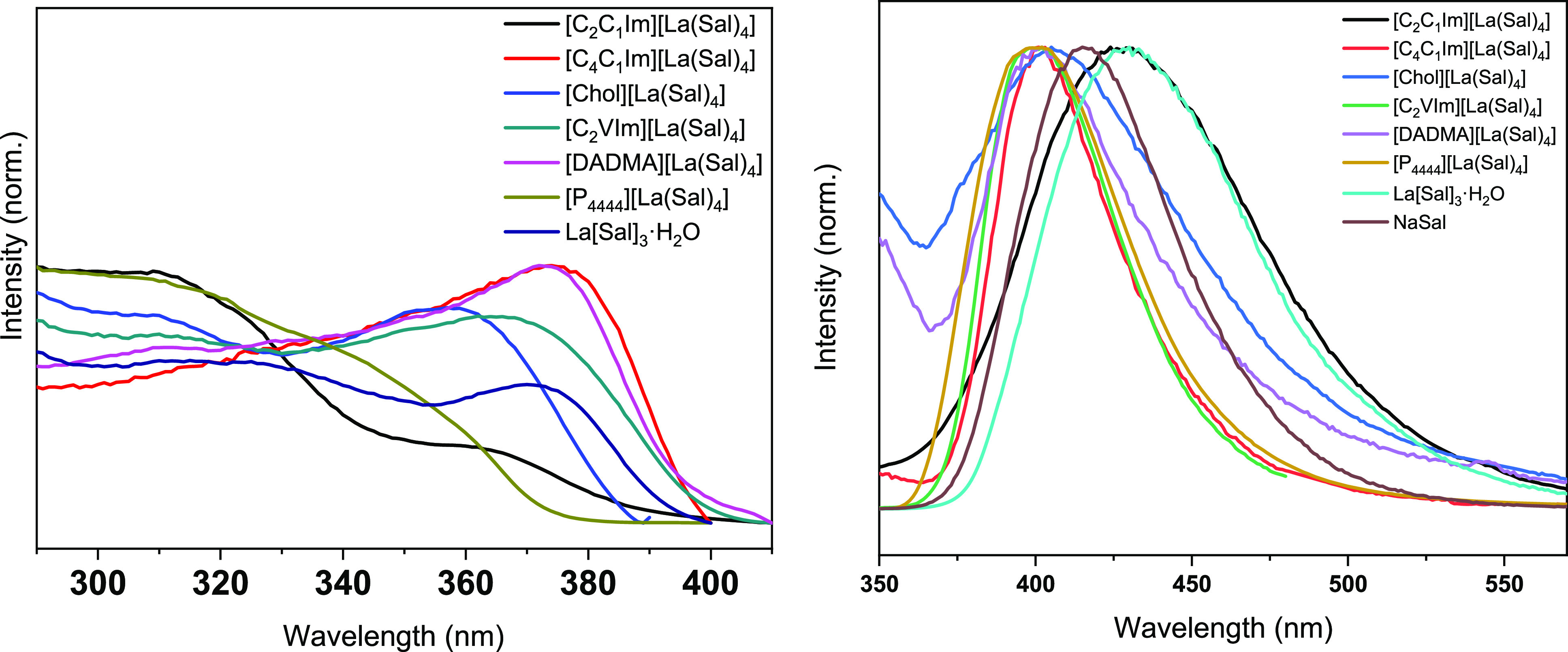
(Left) Normalized emission spectra of the lanthanum compounds, λ_ex_ = 310 nm. (Right) Normalized excitation spectra of the lanthanum compounds, λ_em_ = 430 nm for [C_4_C_1_Im][La(Sal)_4_] and for the other compounds, λ_em_ = 410 nm.

**Figure 4:**
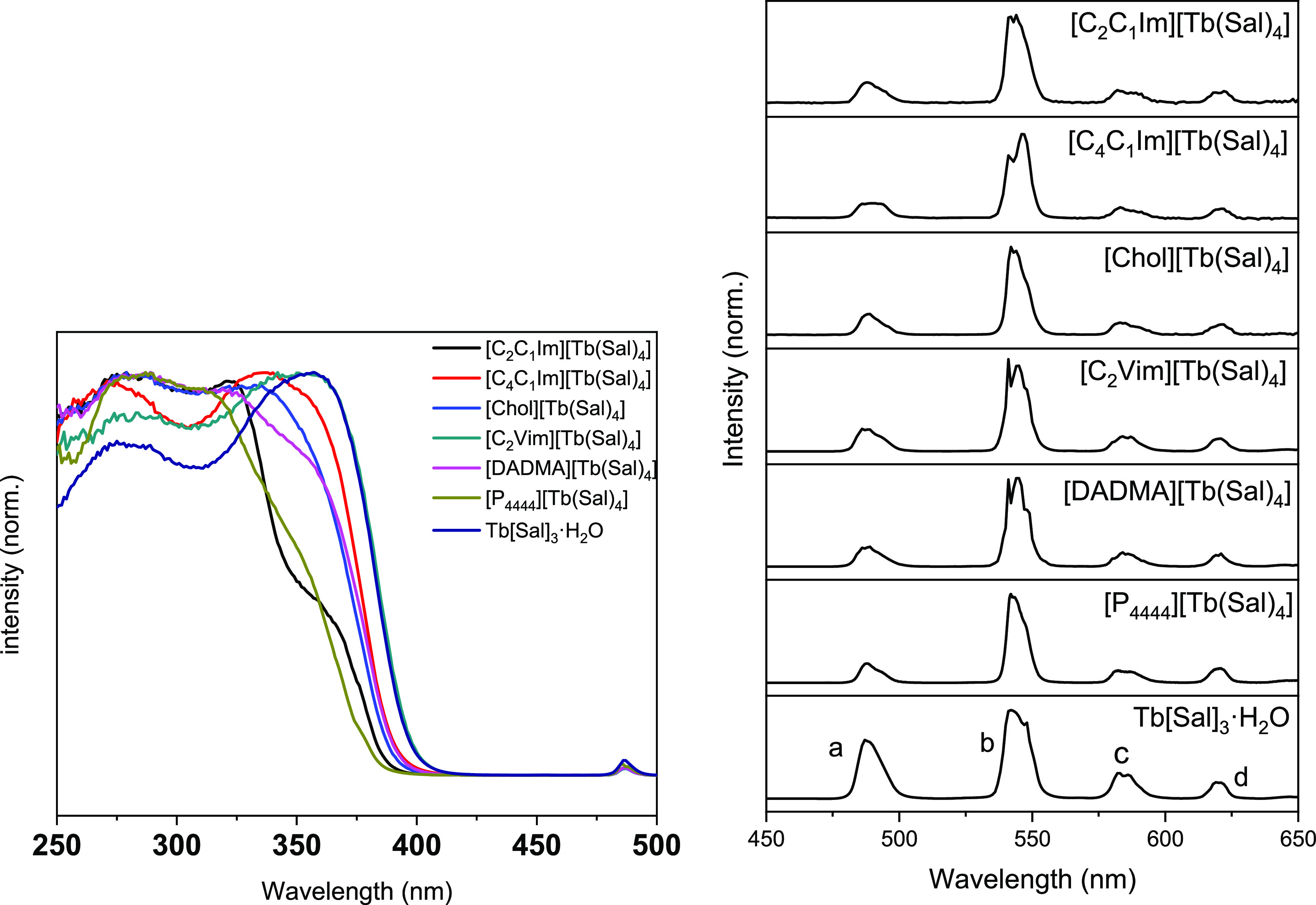
(Left) Normalized photoemission spectra of terbium compounds, λ_ex_ = 310 nm. (Right) Normalized excitation spectra of the terbium compounds, λ_em_ = 542 nm. The observed transitions are labeled in the bottom spectrum: (a) ^5^D_4_→^7^F_6_; (b) ^5^D_4_→^7^F_5_; (c) ^5^D_4_→^7^F_4_; (d) ^5^D_4_→^7^F_3_.

All lanthanum salts show a strong absorption in the region around 300–350 nm which can be ascribed to π-π transitions of the salicylate anion.^38^ In accordance with the absorption maximum derived from the UV–vis absorption spectra (see the example of the absorption spectrum of La(Sal)_3_·H_2_O and Tb(Sal)_3_·H_2_O in the SI, Figure S61), the excitation wavelength was set to the absorption band of the salicylate ligand (310 nm) to collect the emission spectra presented in Figure 3 (left). A broad emission with a maximum at 400 nm located in the range of 360–540 nm is observed for the lanthanum compounds. In general, the spectra display very similar features to the one of NaSal^38^ which is also shown in Figure 3 (left). This confirms that the origin of the broad band emission of the lanthanum compounds originates from π-π transitions within the salicylate anion, This phenomenon finds its roots in the. The C_2_C_1_Im compound shows a bathochromic shift of the emission band center to 430 nm. Despite that several factors are involved, especially in the presence of imidazolium moieties, this effect can be attributed to a different molecular packing,^39^ particularly when the proximity of ions results in a different conjugation and energy decrease of the emissive levels. Since the C_2_C_1_Im cation bears two short alkyl chains, it is probably characterized by a better packing in the crystal structure. This causes a closer proximity of the salicylate moieties compared to the other compounds, which increases the probability of π-π interactions that lead to an energy decrease, which is responsible for a red shift in luminescent materials in the solid state.^51^ The excitation spectra of the lanthanum compounds (Figure 3, right) were measured by monitoring the emission of the salicylate ligand present at 430 nm. This allowed us to establish the relative position of the salicylate states with respect to the terbium levels. These spectra show a broadband which can be attributed to the π→π* transitions, as also observed in the absorption spectra (Figure S60). The terbium complexes excitation spectra were recorded by detecting the maximum emission peak (542 nm), corresponding to the ^5^D_4_→^7^F_5_ transition of Tb^3+^, show a broadband extending throughout the measured region, corresponding to salicylate π→π* transitions (Figure 4, left). Of the typical narrow *f*-*f* excitation bands of Tb^3+^, only the ^7^F_6_→^5^D_4_ transition is detectable with low intensity at 487 nm, confirming a very efficient energy transfer between the salicylate ligand and the Tb^3+^ ion. When the terbium compounds are excited into the salicylate ligand at 310 nm (Figure 3, left), the complexes exhibit strong green emission with the typical ^5^D_4_→^7^F_6_, ^5^D_4_→^7^F_5_, ^5^D_4_→^7^F_4_, and ^5^D_4_→^7^F_3_*f*-*f* transitions (bands centered at 488, 542, 582, and 620 nm, respectively) (Figure 4, right). The absence of the features of the salicylate emission in the spectra of the terbium compounds indicates an efficient energy transfer from the ligand to the terbium cation, as also proven by the excitation spectra (Figure 4, left). As can be inferred from the excitation spectra, all the samples can be excited in the salicylate band. The light absorbed by the singlet state of the ligand (corresponding to energy levels in the region between 25 000 and 35 000 cm^–1^ as observed in the excitation spectrum at room temperature (RT), Figure 4, right) can be transferred to the triplet state through intersystem crossing, as frequently observed for similar systems.^52^ For the La(III) complexes, only the ligand-localized fluorescence of salicylate appears, due to the lack of metal-centered energy states.^53^ On the other hand, for the Tb(III) compounds, suitable energy levels are available and an intramolecular energy transfer occurs from either the singlet or the triplet excited states of the ligand to the metal.^54^ According to Latva’s rule,^52^ the feeding ligand level should be about 1850 cm^–1^ higher than the Tb^3+^ emissive level to avoid back transfer from the metal to the ligand. The triplet level of the salicylate ligand was determined by measuring the phosphorescence of the La(Sal)_3_·H_2_O complex at low temperature and was found to be around 457 nm (Figure S62, left (SI)), corresponding to 21 880 cm^–1^.^55,56^ This value is in line with the data reported by Yang et al.,^57^ which states that the triplet state of the salicylate ligand is at 23 300 cm^–1^. This band overlaps with the ^5^D_4_ levels of Tb(Sal)_3_·H_2_O (Figure S62, left (SI)). However, the energy differences between the triplet level of La(Sal)_3_·H_2_O and the ^5^D_4_ level of Tb(Sal)_3_·H_2_O (20 568 cm^–1^)^58^ is 1506 cm^–1^, too small to avoid back transfer.^56,57^ On the other hand, temperature-dependent effects responsible for back transfer were not detected. First, salicylate emission is not visible in the emission spectra neither at RT nor at a low temperature of Tb complexes (Figure 4, left, and Figure S62, left (SI)). Second, the presence of nonradiative transfer can also be excluded since the lifetime did not change at low *T* (Figure S62, right (SI)). As a consequence, the involvement of the triplet state in the energy transfer from the ligand to Tb^3+^ can be ruled out, or, if present, it is negligible compared to the spin-allowed transition from the singlet state.

The energy transfer efficiency is enhanced by a short distance between the activator species and the sensitizer. This is provided by the binding of the Tb cation to the salicylate ligand. This allows a good overlap between the absorption/excitation spectra of Tb(Sal)_3_·H_2_O with the emission spectrum of La(Sal)_3_·H_2_O (Figure S63 (SI)).

Radiative emission then occurs from this state into the ^7^F_6-3_ levels (Figure S65).

Quantum yields (QYs) were determined upon excitation into the salicylate band (Table 2). Note that it is not possible to selectively excite into Tb^3+^ levels and determine the sensitization quantum yields because of spectral overlap. In the case of the terbium complexes, quantum efficiencies in the range between 40% and 63% have been observed, significantly improved compared to the quantum yield of the simple Tb(Sal)_3_·H_2_O (25%). The improved quantum yield may be explained by looking at the environment of the activator ion. The crystal structure of Tb(Sal)_3_·H_2_O is unknown, and no crystal of sufficient quality for X-ray structural determination could be produced during this study. However, PXRD reveals Tb(Sal)_3_·H_2_O to crystallize isotypic with the analogous samarium compound, Sm(Sal)_3_·H_2_O, whose crystal structure has been reported.^59^ In Sm(Sal)_3_·H_2_O, the coordination sphere of each Sm(III) ion contains one water molecule. Vibrational quenching of luminescence by water is well-known and caused by OH stretching vibrations. Therefore, the absence of water in the anhydrous compounds is reflected in their superior QYs compared to the hydrated Tb(Sal)_3_·H_2_O species. Moreover, Tb(Sal)_3_·H_2_O has also the shortest lifetime. The observed lifetimes correlate well with the QYs as a decreased quenching leads to an increase in lifetimes. The highest lifetimes have been observed for [DADMA][Tb(Sal)_4_] (1.43 ms) and [Chol][Tb(Sal)_4_] (1.50 ms); the former is also characterized by the highest QY values of 63%. In these two samples, the cation does not bear an aromatic moiety. [P_4444_][Tb(Sal)_4_] exhibits the lowest quantum yields and contains a bulky cation with highly flexible alkyl side chains which should make vibronic deactivation increasingly likely. Thus, the water in the first coordination sphere of the RE(III) ion, but also to the cation in the second coordination sphere, and differences in crystal packing all have a direct effect on the photophysical properties of the different compounds. Bulky effects and the distribution of polar and nonpolar domains imposed by the different cations employed all influence the properties. As previously, the salicylate compounds have been compared to the same molecules as in the thermal description. The lifetime with the salicylate ligand is twice the value compared to the [C_2_C_1_Im][Tb(DCA)_3_(H_2_O)_4_]^33^ (1.40 ms vs 0.60 ms) which indicates that the antenna effect and the absence of water have a direct influence. Nevertheless, in the case of [C*_n_*C_1_Im][Tb(NO_3_)_3_]^50^ (*n* = 2, 4, 6 or 8), the values for the lifetimes are very similar when the Tb is directly excited (λ_ex_ = 541 nm).

**Table 2:** Lifetimes and Quantum Yields of the Terbium Compounds

compound	quantum yield (%)	lifetime (ms)
[C_2_C_1_Im][Tb(Sal)_4_]	58 ± 3	1.40
[C_4_C_1_Im][Tb(Sal)_4_]	50 ± 6	1.25
[C_2_Vim][Tb(Sal)_4_]	40 ± 2	1.14
[Chol][Tb(Sal)_4_]	58 ± 2	1.50
[DADMA][Tb(Sal)_4_]	63 ± 6	1.43
[P_4444_][Tb(Sal)_4_]	40 ± 3	1.25
Tb(Sal)_3_·H_2_O	25 ± 1	1.03

**Figure 5:**
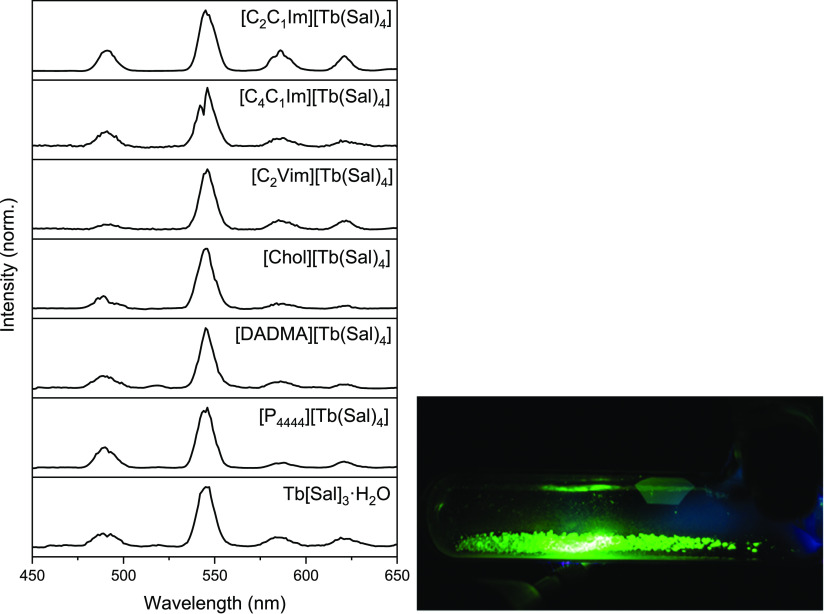
Electroluminescence spectra of Tb compounds. (Left) Emission spectra of all Tb compounds upon excitation with a Tesla generator. (Right) Picture of [C_2_C_1_Im][Tb(Sal)_4_] in a Schlenk flask under vacuum exposed to the high frequency generator.

Finally, in addition to photoexcitation, the terbium samples were excited with an HF Tesla generator. All terbium compounds show an intense green electroluminescence (Figure 5). The emission spectra show the same features as those obtained from photoexcitation.

**Figure 6:**
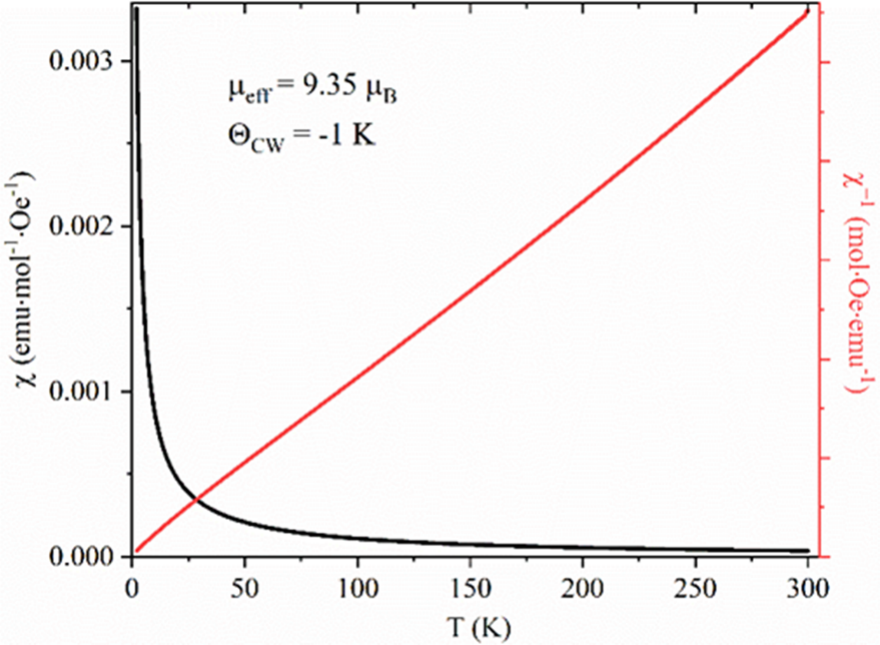
Temperature dependent susceptibility and inverse susceptibility of [C_2_C_1_Im][Tb(Sal)_4_] measured at 0.1 T from 2 to 300 K.

The magnetic properties of [C_2_C_1_Im][Tb(Sal)_4_] as a representative of the series were screened in the temperature range from 2 to 300 K and in an applied magnetic field of 1000 Oe (Figure 6). The linear temperature dependence of the inverse susceptibility indicates that the Curie–Weiss law is followed in the entire temperature range. No magnetic ordering could be observed down to 2 K. The linear fit of χ^–1^(*T*) yields the Weiss temperature Θ_CW_ = −1 K, indicating negligible interaction between the metal ions in the complex. The room temperature χ*T* value of the compound is 10.9 cm^3^·K·mol^–1^, in fairly good agreement with the predicted value for non-interacting Tb^3+^ ions.^60^ This value remains practically constant in the range 300–100 K and decreases rapidly below 25 K to 25.4 cm^3^·K·mol^–1^ at 2 K. The effective paramagnetic moment per formula unit at room temperature was calculated according to the formula μ_eff_ = √(8χ_M_*T*) and is equal to 9.35 μ_B_.

## Conclusion

Salicylate ionic liquids were used with lanthanum and terbium cations to form two sets of complexes. Their identity and purities have been confirmed by using ^1^H NMR, IR, mass spectrometry, and optical spectroscopy. In the lanthanum complexes, only the emission originating from the ligand could be detected. On the other hand, terbium complexes exhibited intense green photoluminescence. This emission can be observed upon excitation in the ligand’s absorption region. This phenomenon is caused by a highly efficient energy transfer from the ligand to the metal center. The absence of water in the terbium materials contributes to a lower level of vibrational quenching, compared to the simple Tb(Sal)_3_·H_2_O, which allows them to reach high quantum yields, up to 63%. Strong electroluminescence could be observed for all of the terbium-containing materials, making them good candidates for emitter materials in signaling and lighting applications. An advantage is the good thermal stability as the Tb compounds show a strong response to applied external fields, but no signs of cooperative magnetic effects or magnetic ordering.

## Supporting Information Available

^1^H NMR, ^31^P NMR, FT-IR spectra, ESI-MS, TGA, DSC, POM and optical spectra, as well as details of X-ray diffraction experiments (PDF)

## Accession Codes

CCDC 2065152 and 2065153 contain the supplementary crystallographic data for this paper. These data can be obtained free of charge via www.ccdc.cam.ac.uk/data_request/cif, or by emailing data_request@ccdc.cam.ac.uk, or by contacting The Cambridge Crystallographic Data Centre, 12 Union Road, Cambridge CB2 1EZ, UK; fax: +44 1223 336033.
