# Supplementary material for: Magnetic, Photo- and Electroluminescent: Multifunctional Ionic Tb Complexes
Source: Inorg Chem. 2021 Nov 16;60(23):17487–97. doi: 10.1021/acs.inorgchem.1c01875 (PMC8653220; doi:10.1021/acs.inorgchem.1c01875)
Supplement: Supplementary file 1 — ic1c01875_si_001.pdf [file ic1c01875_si_001.pdf]

Supporting Information

Magnetic, photo- and electroluminescent: Multifunctional ionic Tb-complexes

Guillaume Bousrez, Olivier Renier, Veronica Paterlini, Volodymyr Smetana, and Anja-Verena Mudring*

Department of Materials and Environmental Chemistry, Stockholm University, Svante Arrhenius väg 16 C, 10691 Stockholm, Email: anja-verena.mudring@mmk.su.se

Content on 38 pages: 66 Figures and 2 Tables

Table of Contents

I.	Structure information	SI-2
II.	Powder X-Ray diffraction patterns	SI-5
III.	^{1}H-NMR spectra	SI-7
IV.	Infrared spectra	SI-14
V.	Mass spectrometry spectra (ESI-MS)	SI-24
VI.	Thermal analyses	SI-26
VII.	Polarized optical microscopy (POM) micrographs	SI-34
VIII.	UV-Vis spectra	SI-35
IX.	References	SI-38

I. Structure information

Table S1. Basic crystallographic data for $[C_2C_1Im]_4[RE_4(Sal)_{16}(H_2O)_2]$, RE = La, Tb.

	$(C_2C_1im)_4[La_4(Sal)_{16}(H_2O)_2]$	$(C_2C_1im)_4[Tb_4(Sal)_{16}(H_2O)_2]$
CCDC	2065152	2065153
Empirical formula	$La_4C_{136}H_{120}N_8O_{50}$	$Tb_4C_{136}H_{120}N_8O_{50}$
Z	1	1
Molecular mass (g/mol)	3222.05	3302.13
Crystal system	triclinic	triclinic
Space group	$P\bar{1}$ (no. 2)	$P\bar{1}$ (no. 2)
Temperature (K)	170(2)	170(2)
a (Å)	13.180(2)	13.136(2)
b (Å)	13.250(2)	13.253(2)
c (Å)	20.834(3)	20.636(2)
α (°)	82.31(2)	82.72(2)
β (°)	78.58(2)	78.22(2)
γ (°)	71.50(2)	72.15(2)
Volume	3372.3(9)	3339.8(9)
Density (calculated, g/cm³)	1.59	1.63

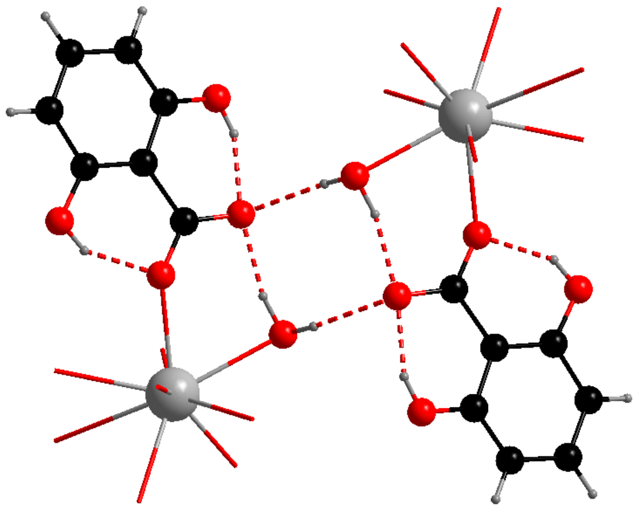

Figure S1. Connectivity scheme between two tetrameric units. (RE atoms are shown in grey, O – red, C – black and H – white).

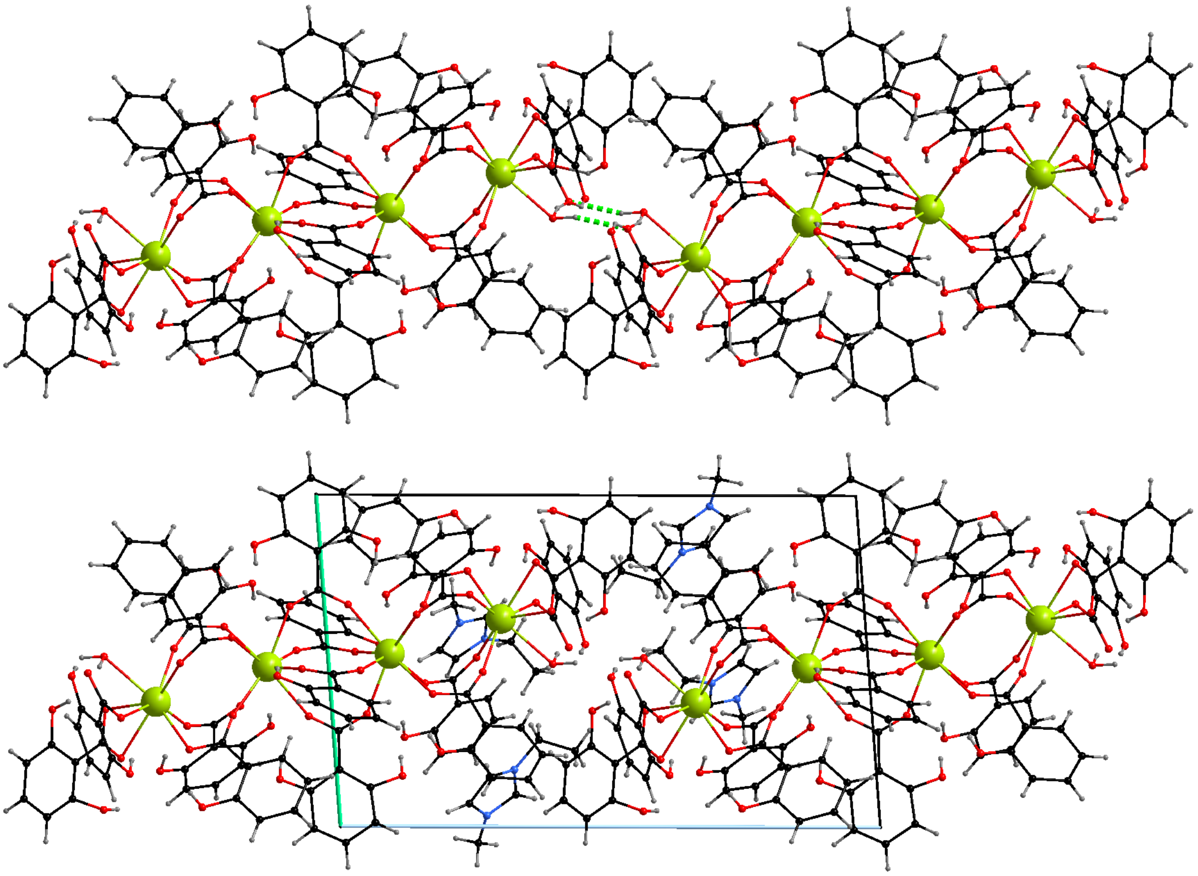

Figure S2. Connectivity between the polyanions along the *c* axis. OH···O contacts have been shown as dashed green lines (*top*) and positions of the cations in the crystal structure (*bottom*). (RE atoms are shown in green, O – red, N – blue and C – black).

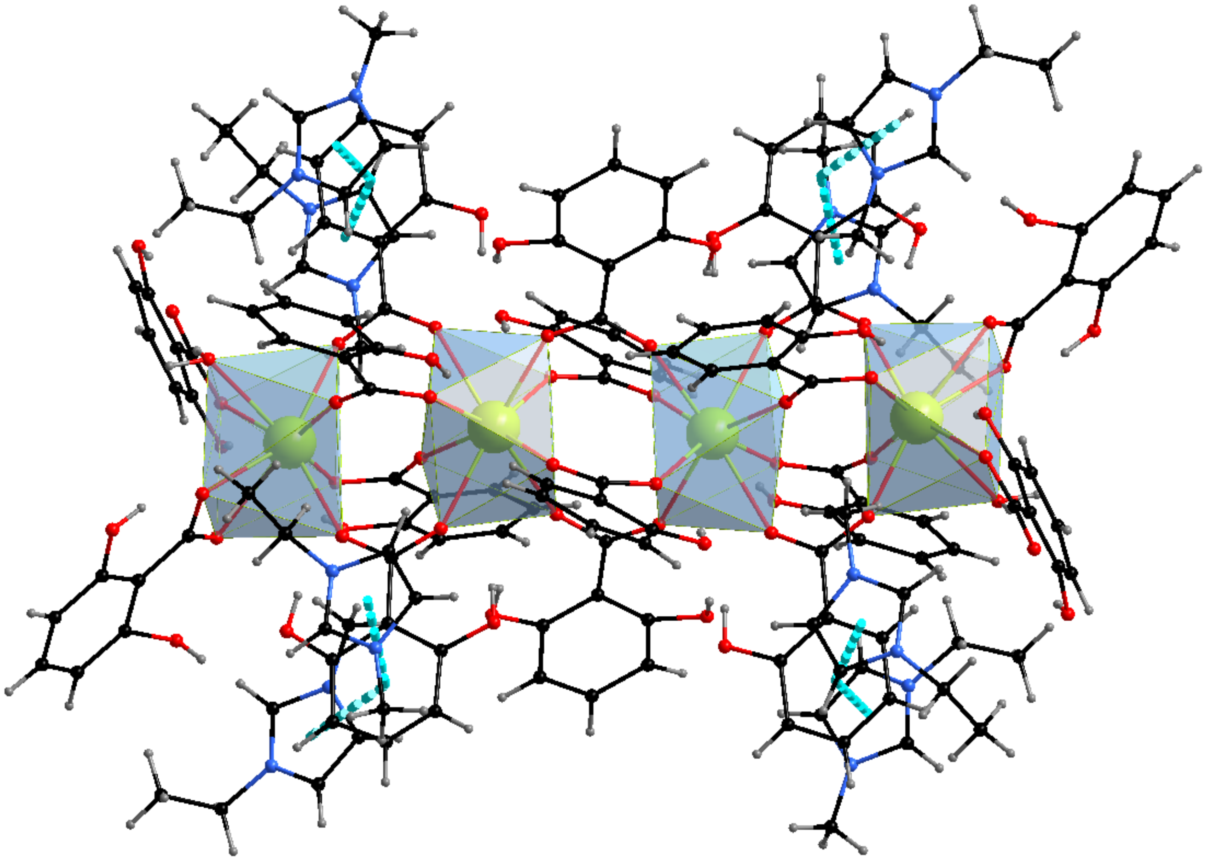

Figure S3. Short contact environment around the RE polyanion. C_g-C_g contacts have been indicated with dashed blue lines. (RE atoms are shown in green, O – red, N – blue and C – black).

II. Powder X-ray diffraction patterns

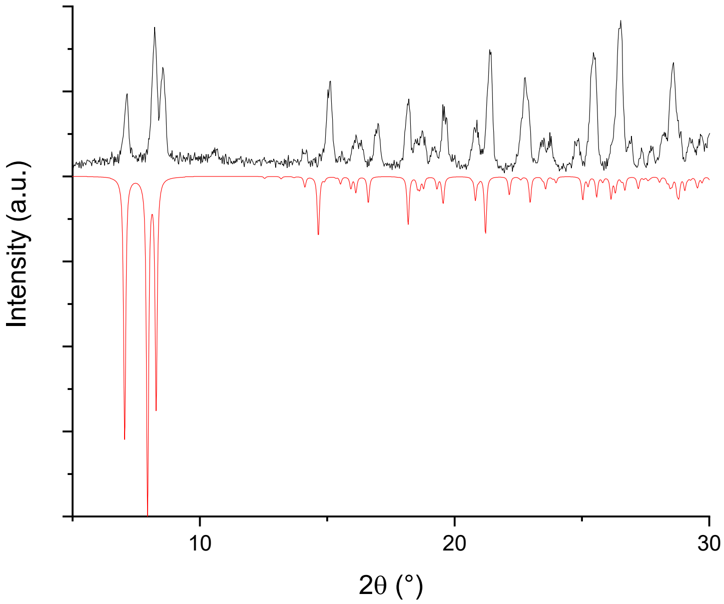

Figure S4. Experimental PXRD pattern of lanthanum salicylate monohydrate ($La(Sal)_3 \cdot H_2O$) (black) *vs* theoretical pattern simulated from the SCXRD of $Sm(Sal)_3 \cdot H_2O$[1] (red).

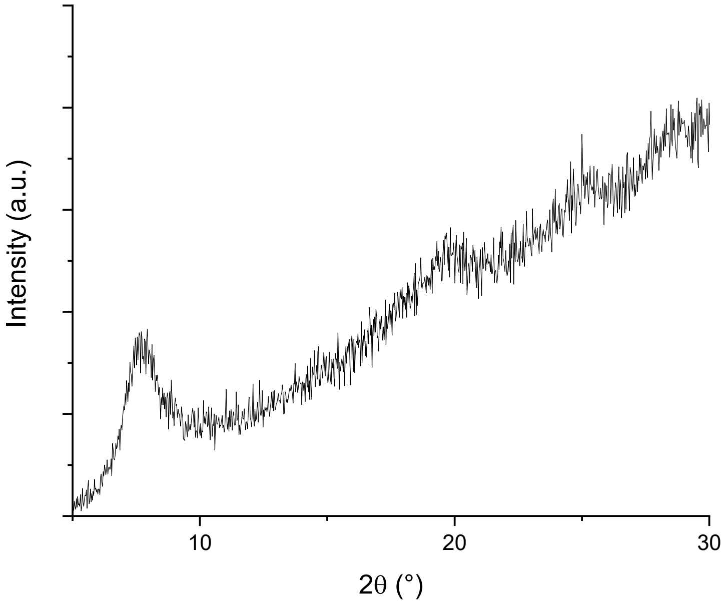

Figure S5. Powder patterns of terbium salicylate monohydrate ($Tb(Sal)_3 \cdot H_2O$).

.

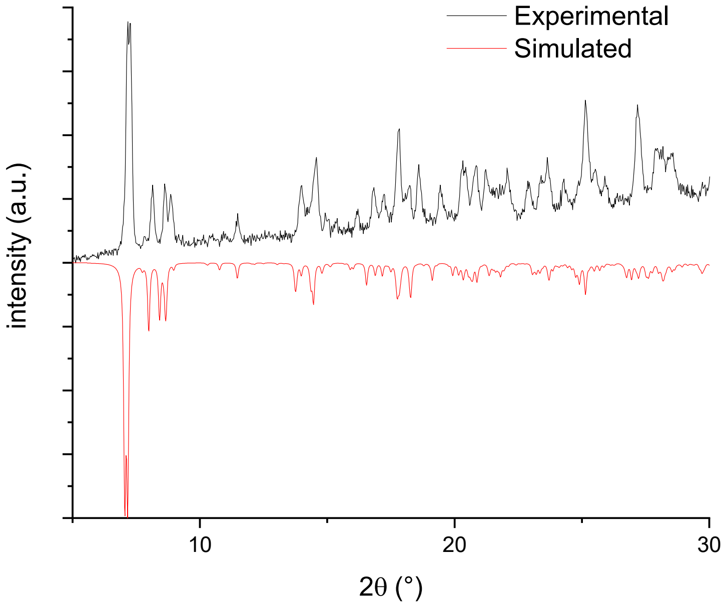

Figure S6. Experimental PXRD pattern of $[C_2C_1Im]_4[La_4(Sal)_{16}(H_2O)_2]$ (black) *vs.* theoretical pattern simulated from the SCXRD of $[C_2C_1Im]_4[La_4(Sal)_{16}(H_2O)_2]$ (red).

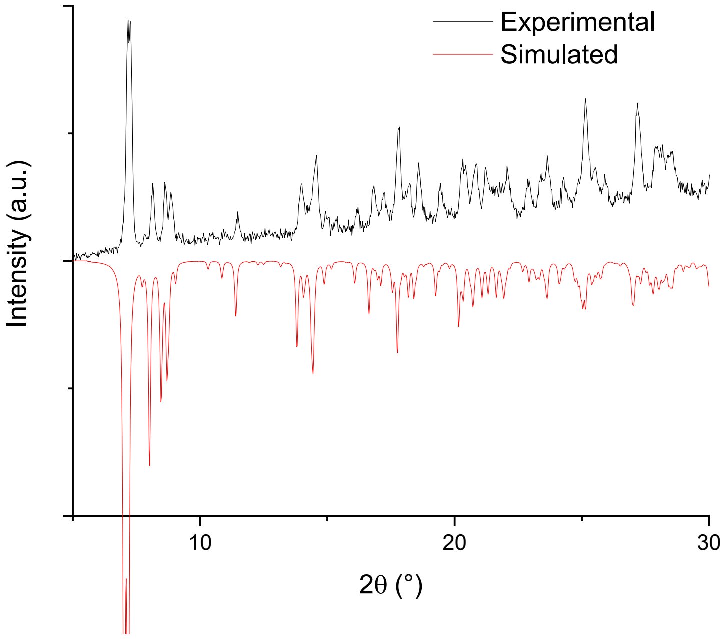

Figure S7. Experimental PXRD pattern of $[C_2C_1Im]_4[Tb_4(Sal)_{16}(H_2O)_2]$ (black) *vs.* theoretical pattern simulated from the SCXRD of $[C_2C_1Im]_4[Tb_4(Sal)_{16}(H_2O)_2]$ (red).

III. ^{1}H-NMR spectra

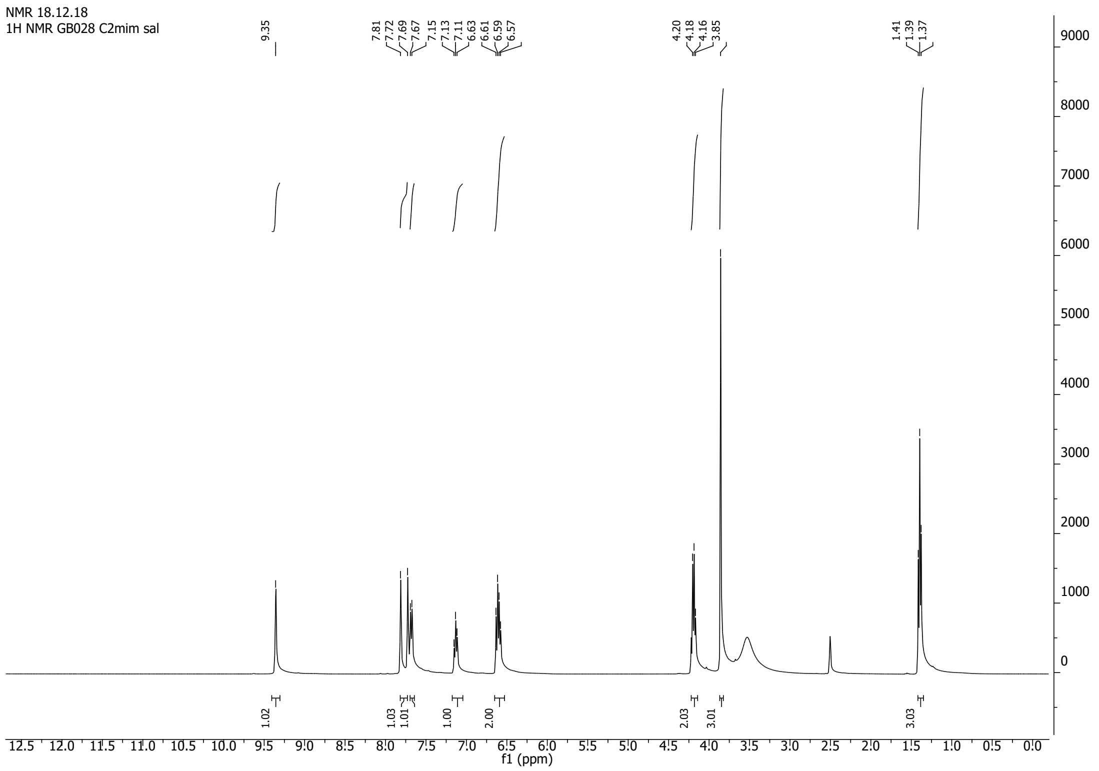

Figure S8. ^{1}H-NMR Spectrum of [C_2C_1Im][Sal] (400 MHz, DMSO).

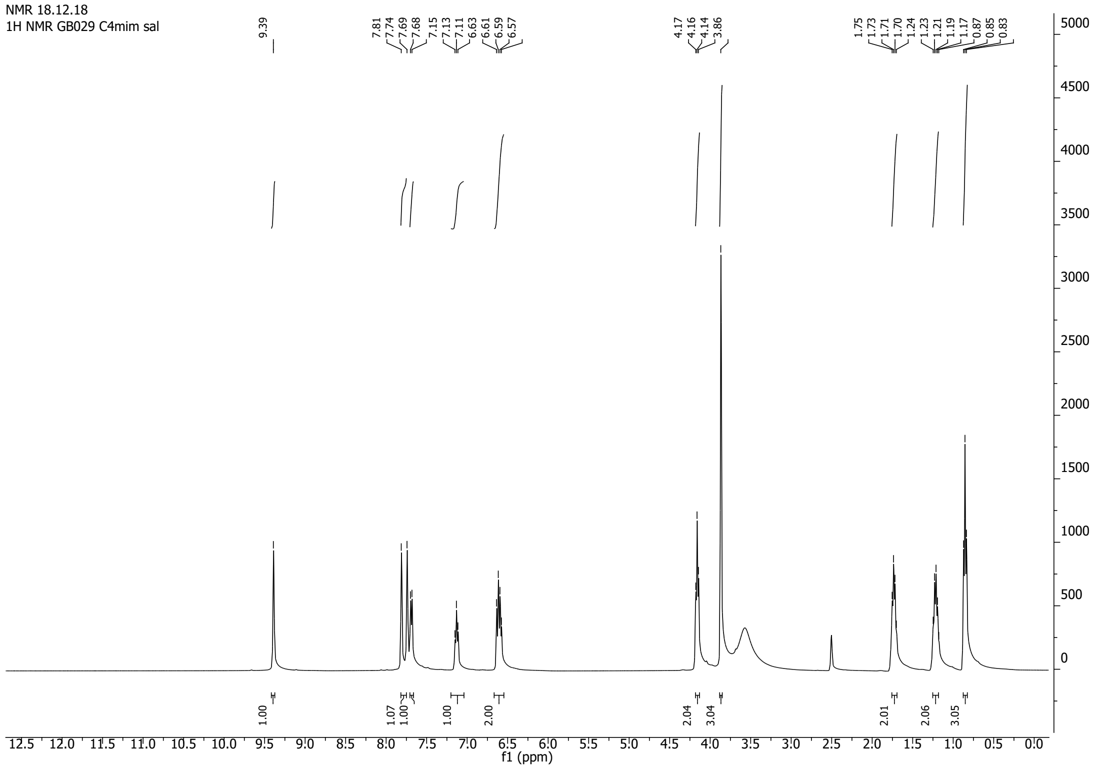

Figure S9. ^{1}H-NMR Spectrum of [C_4C_1Im][Sal] (400 MHz, DMSO).

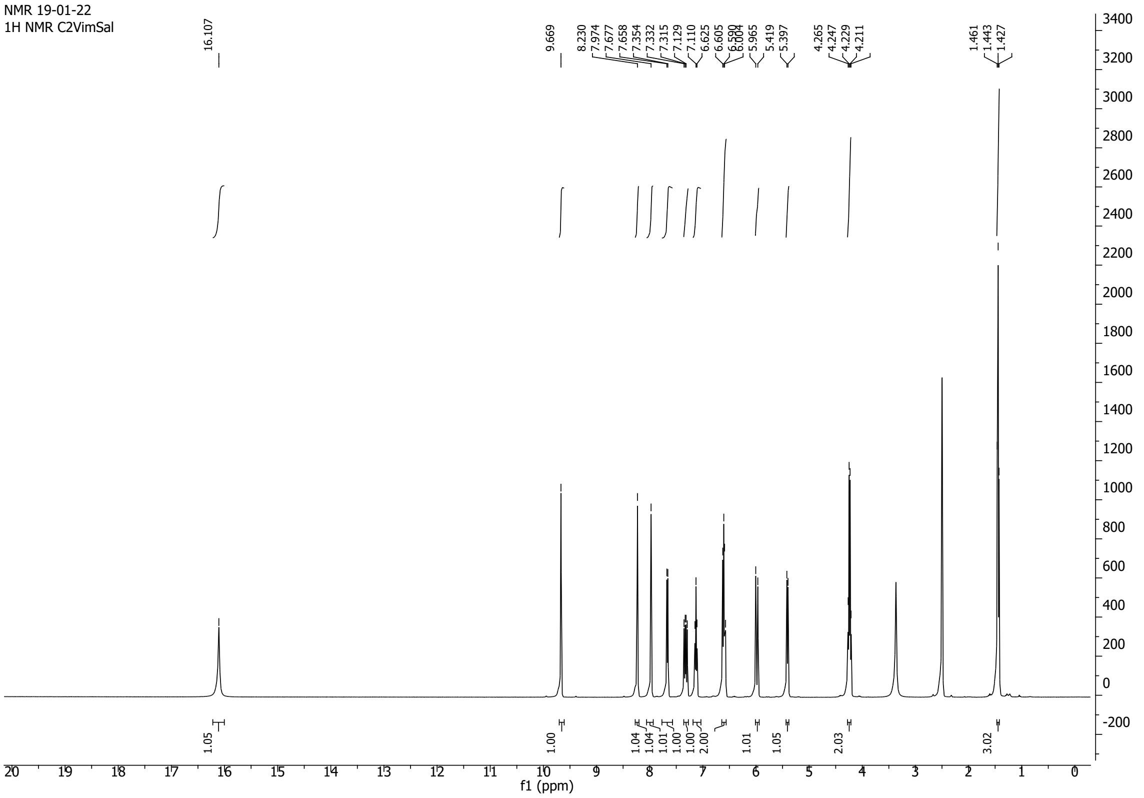

Figure S10. ^{1}H-NMR Spectrum of $[C_2Vim][Sal]$ (400 MHz, DMSO).

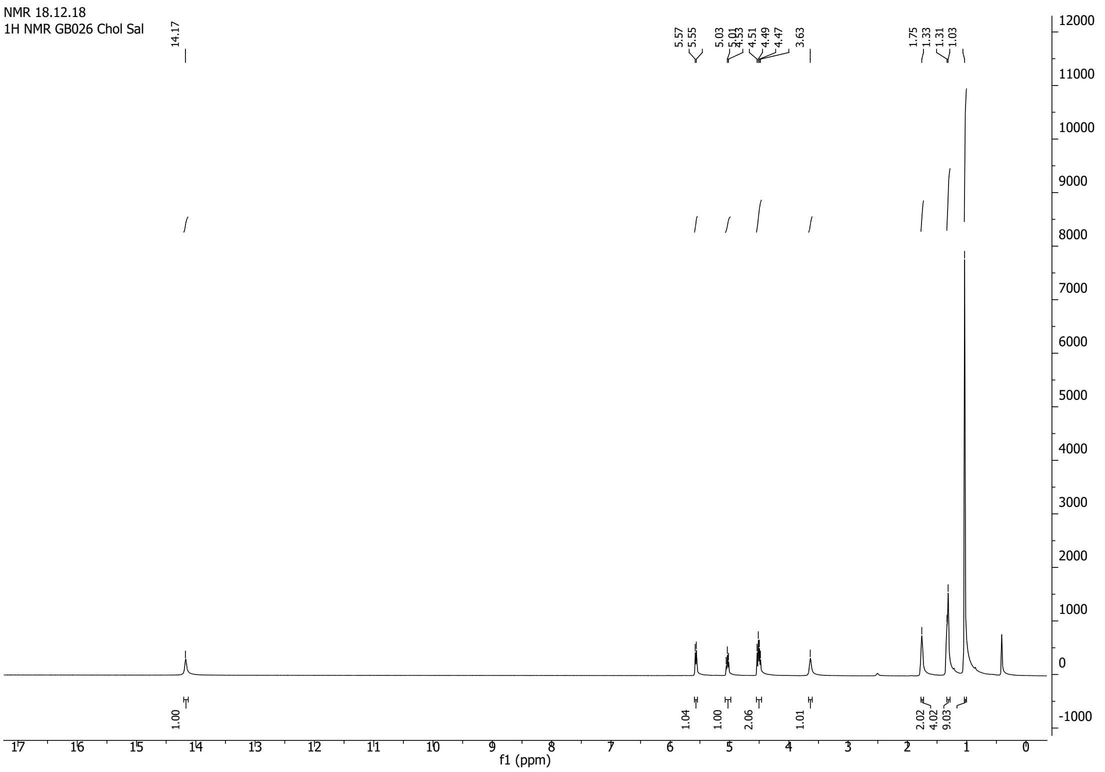

Figure S11. ^{1}H-NMR Spectrum of [Chol][Sal] (400 MHz, DMSO).

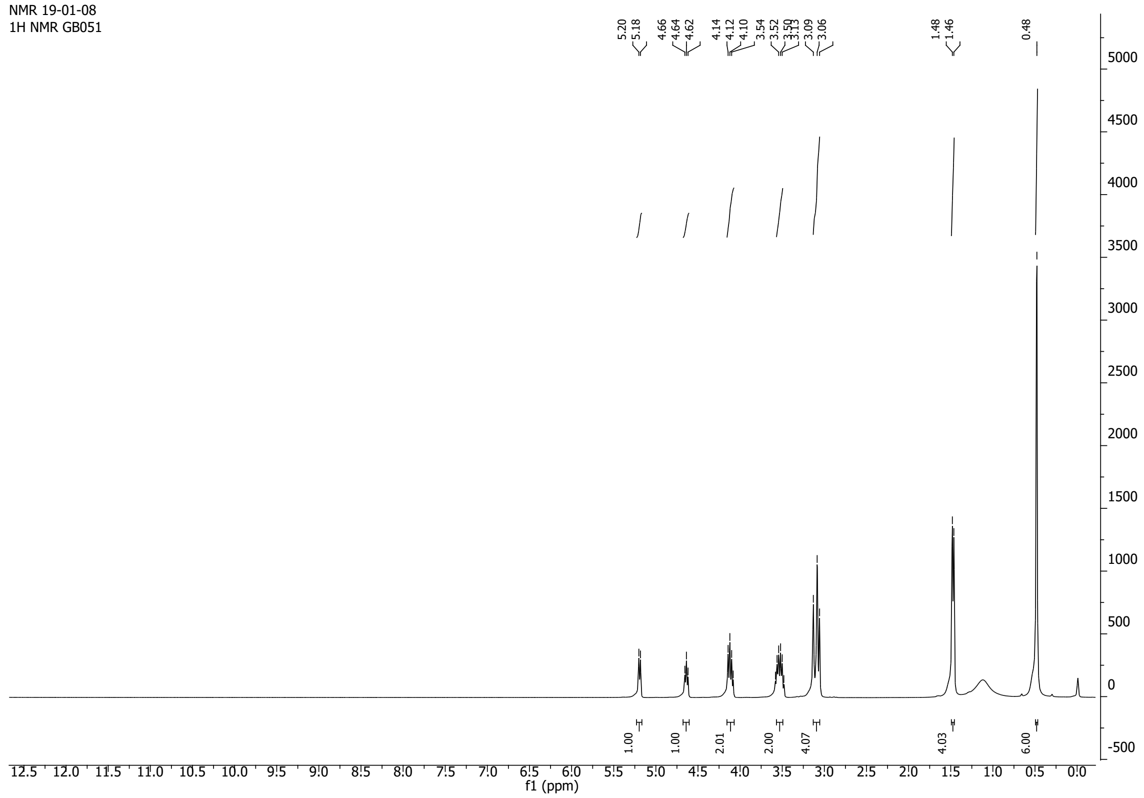

Figure S12. ^{1}H-NMR Spectrum of [DADMA][Sal] (400 MHz, DMSO).

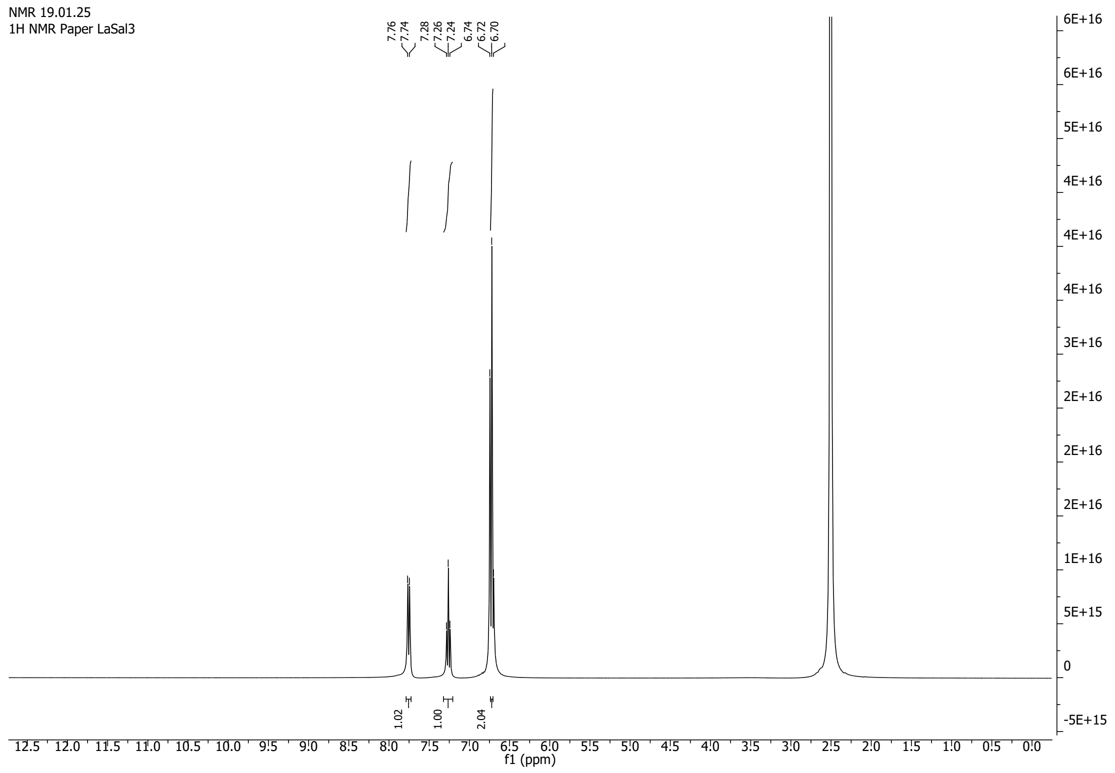

Figure S13. ^{1}H-NMR Spectrum of $La(Sal)_3$•H_2O (400 MHz, DMSO).

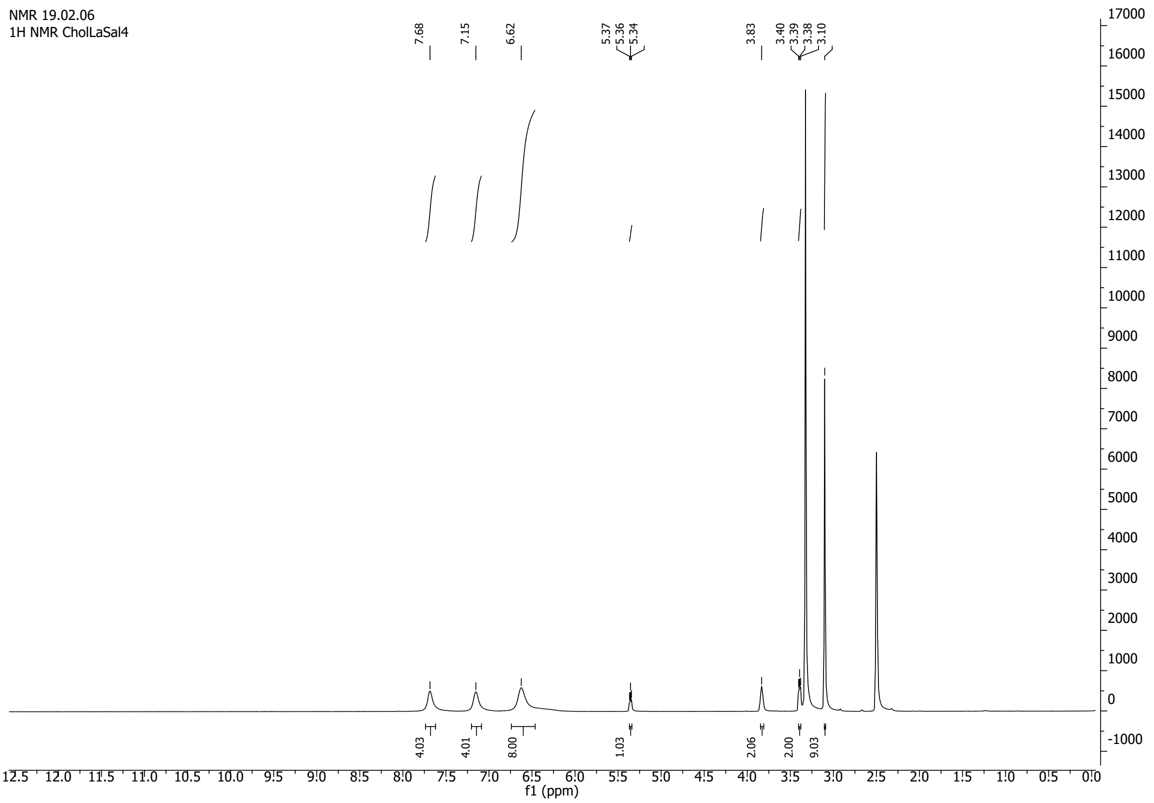

Figure S14. ^{1}H-NMR Spectrum of (Chol)[La(Sal)$_4$] (400 MHz, DMSO).

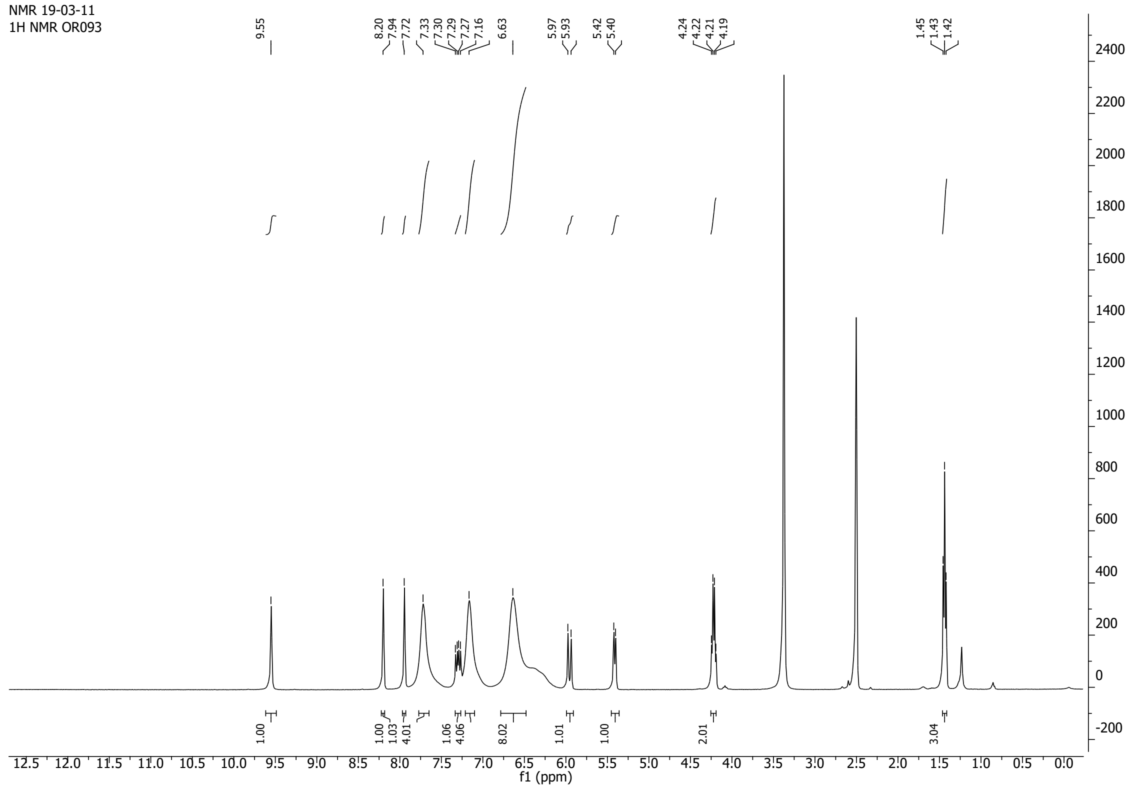

Figure S15. ^{1}H-NMR Spectrum of [C_2Vim][La(Sal)$_4$] (400 MHz, DMSO).

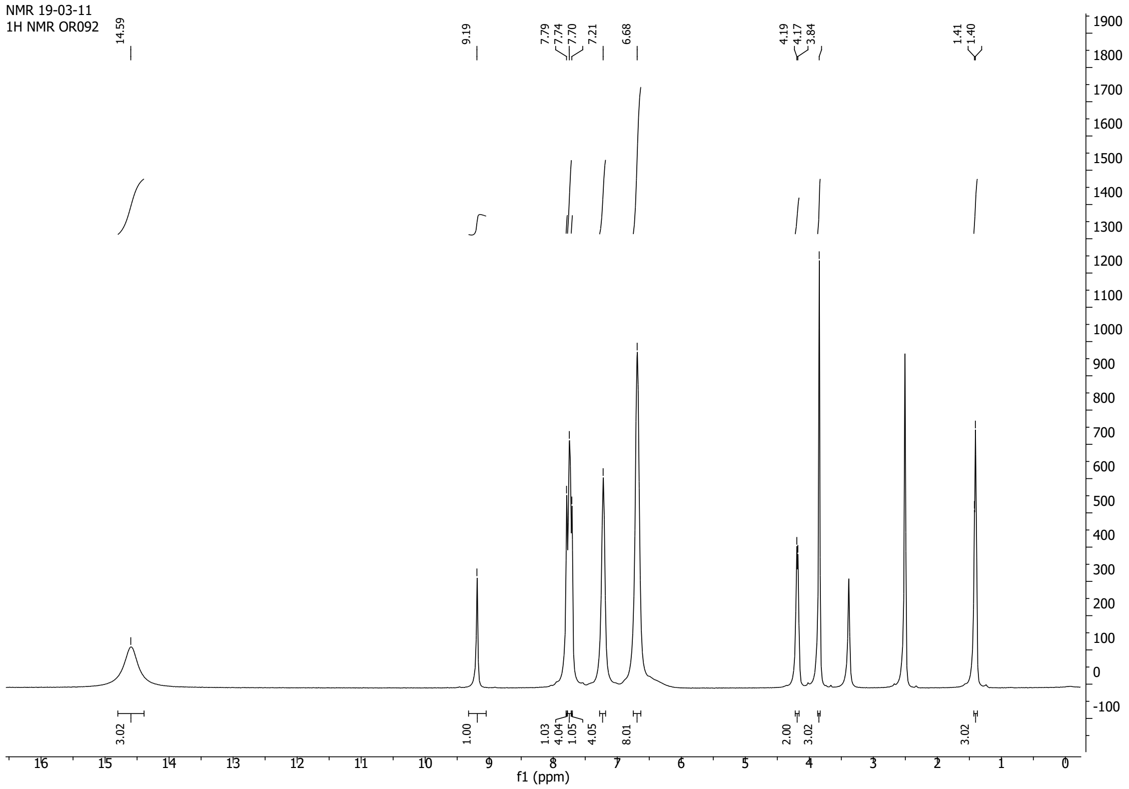

Figure S16. ^{1}H-NMR Spectrum of $[C_2C_1Im][La(Sal)_4]$ (400 MHz, DMSO).

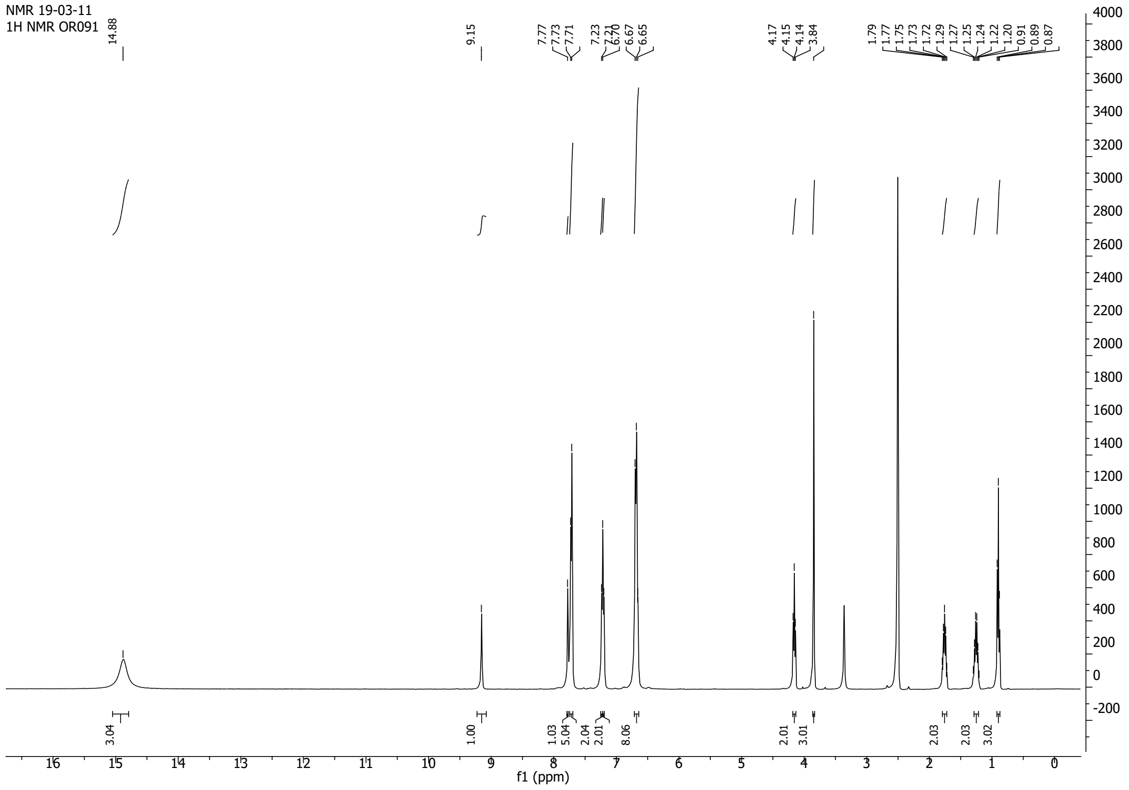

Figure S17. ^{1}H-NMR Spectrum of $[C_4C_1Im][La(Sal)_4]$ (400 MHz, DMSO).

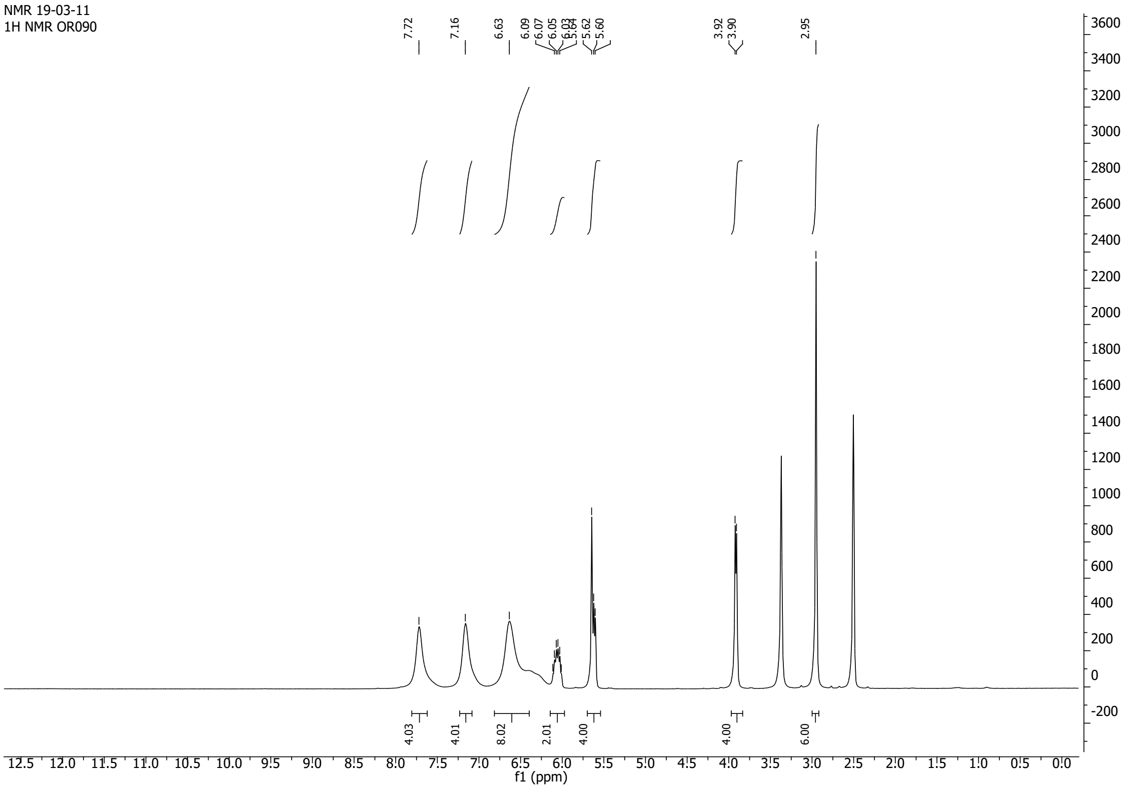

Figure S18. ^{1}H-NMR Spectrum of [DADMA][La(Sal)$_4$] (400 MHz, DMSO).

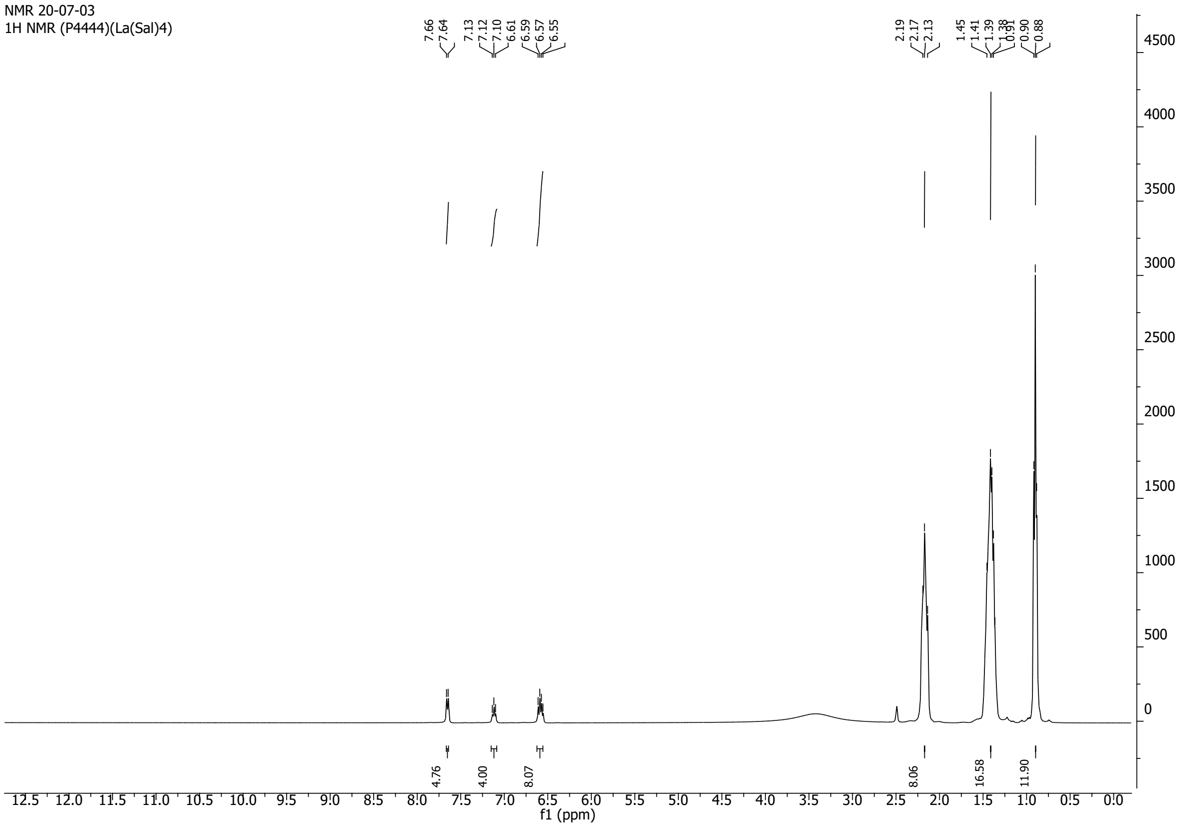

Figure S19. ^{1}H-NMR Spectrum of [P_{4444}][La(Sal)$_4$] (400 MHz, DMSO).

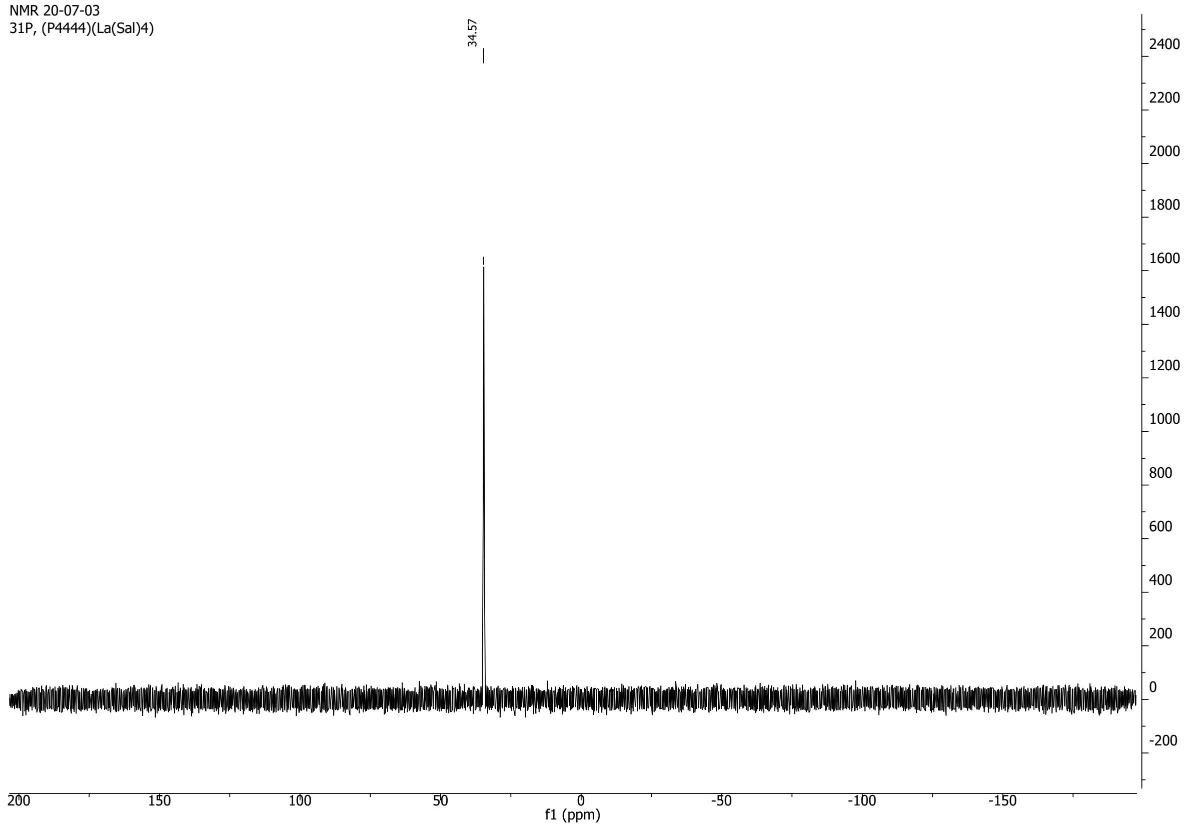

Figure S20. ^{31}P-NMR Spectrum of [P_{4444}][La(Sal)$_4$] (162 MHz, DMSO).

IV. Infrared spectra

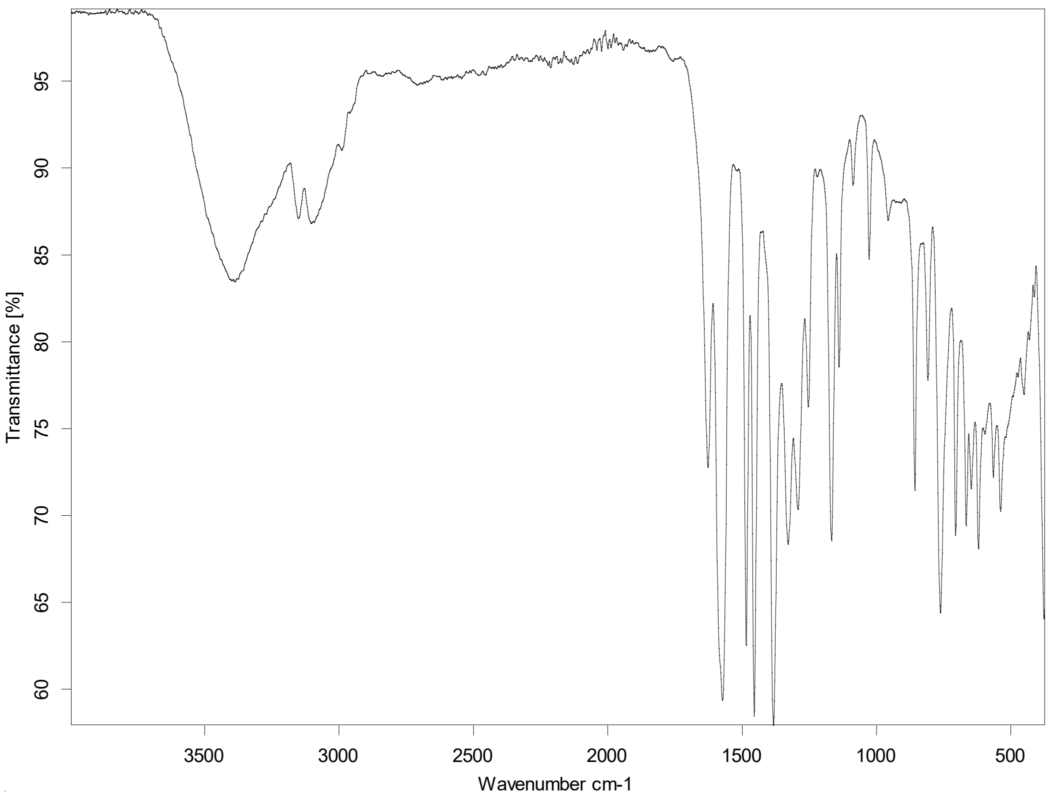

Figure S21. Infrared spectrum of [C_2C_1Im][Sal].

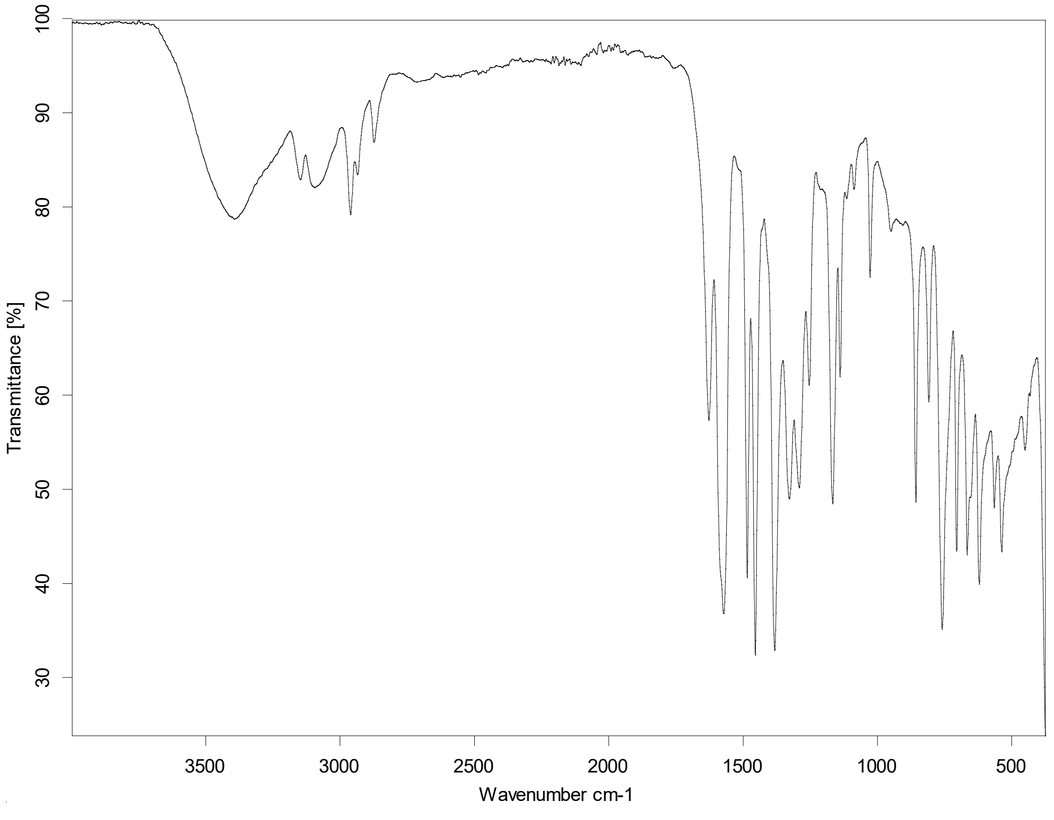

Figure S22. Infrared spectrum of [C_4C_1Im][Sal].

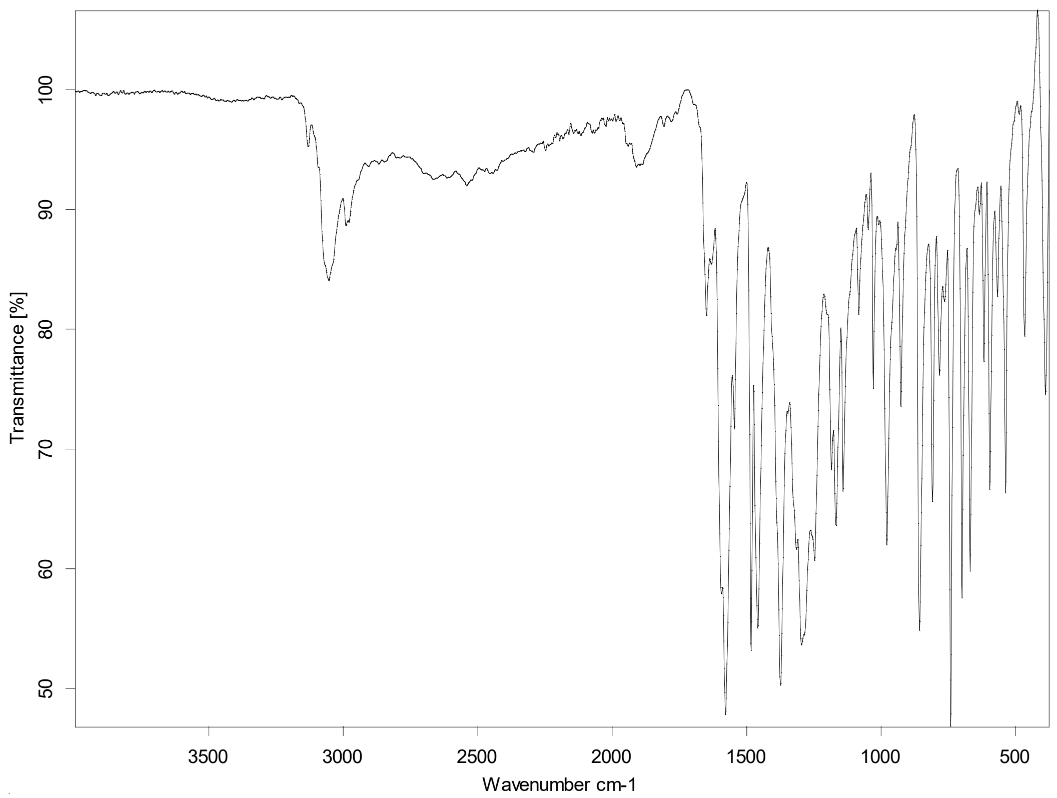

Figure S23. Infrared spectrum of [C_2VIm][Sal].

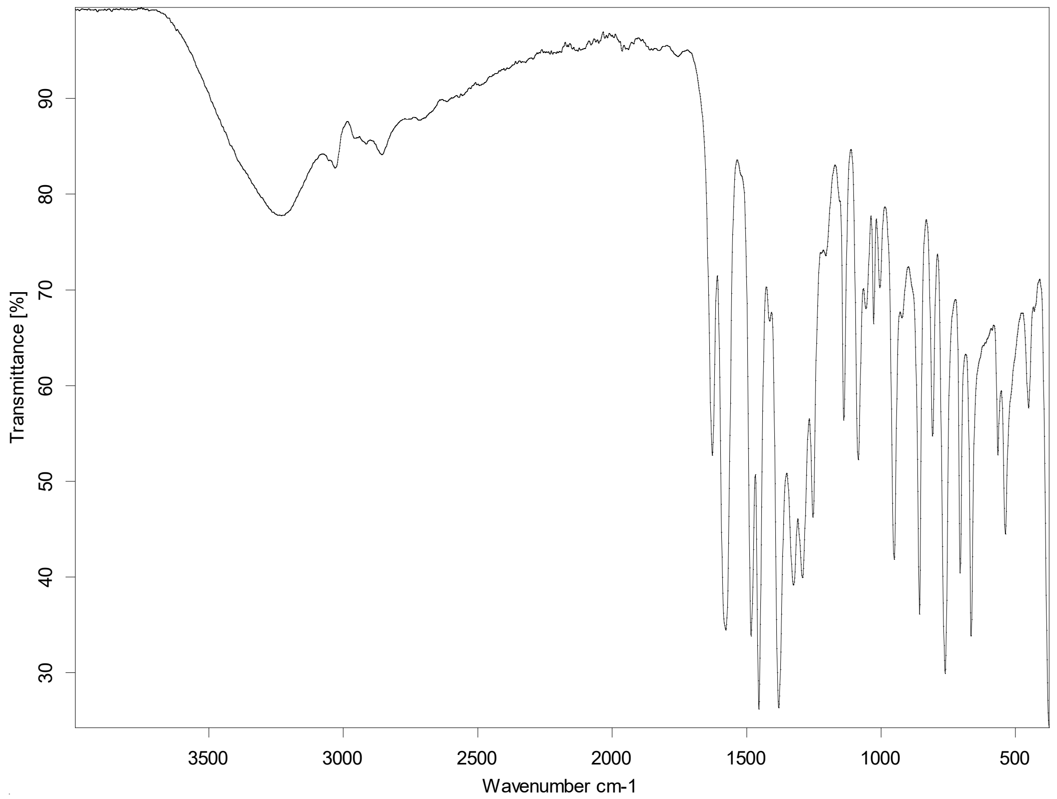

Figure S24. Infrared spectrum of [Chol][Sal].

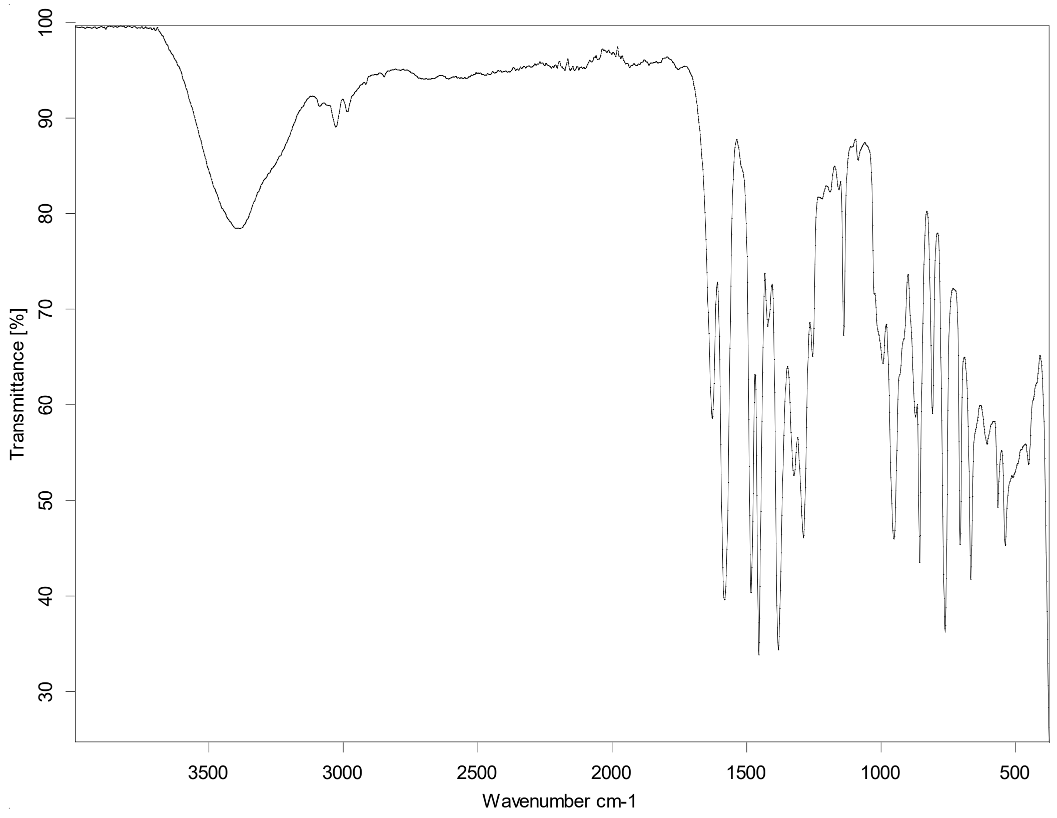

Figure S25. Infrared spectrum of [DADMA][Sal].

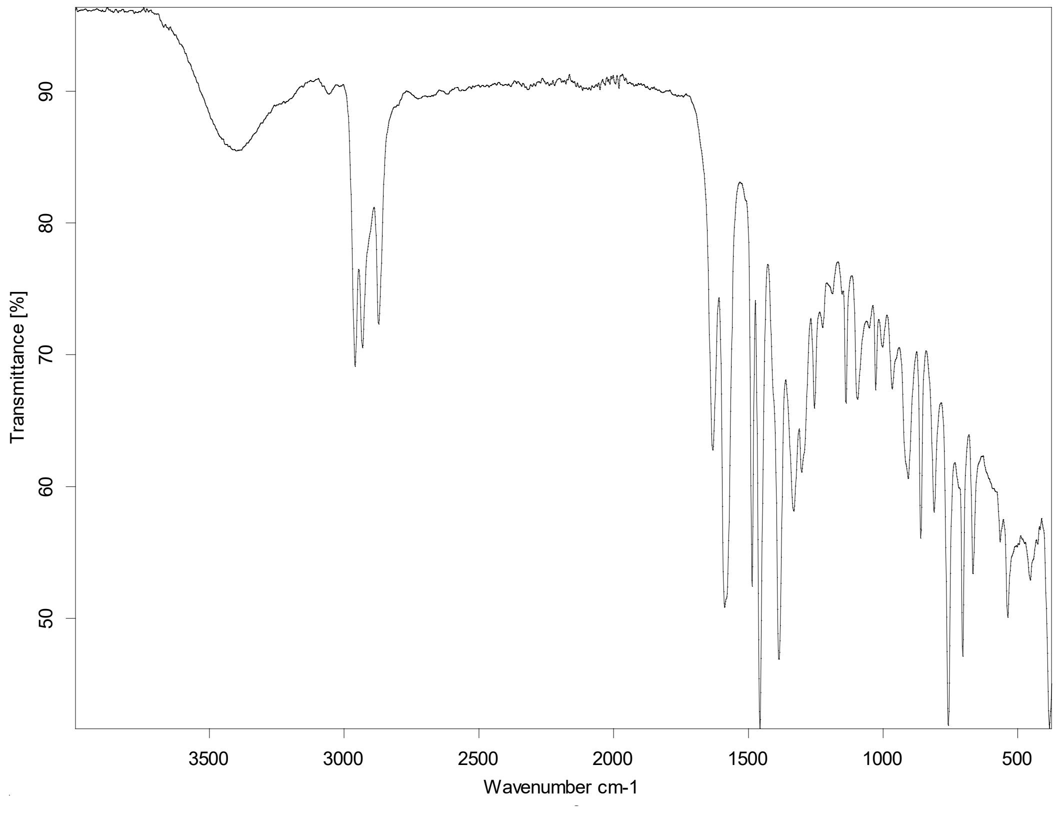

Figure S26. Infrared spectrum of [P_{4444}][Sal].

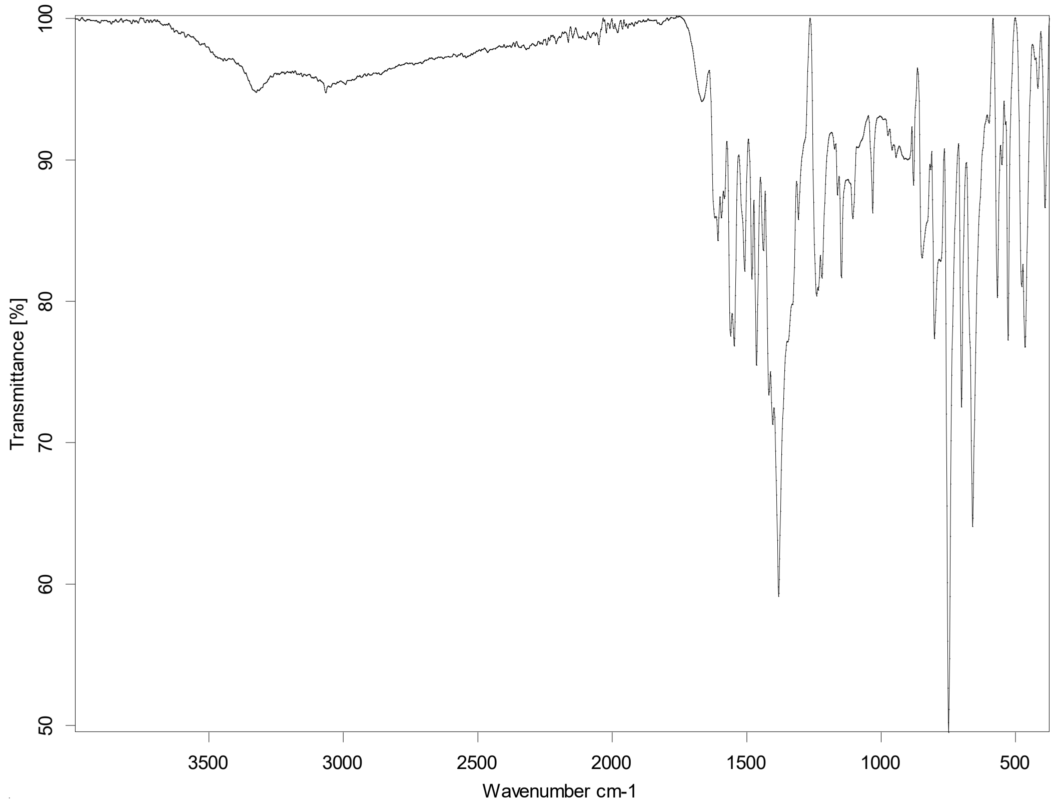

Figure S27. Infrared spectrum of $La(Sal)_3{\bullet}H_2O$.

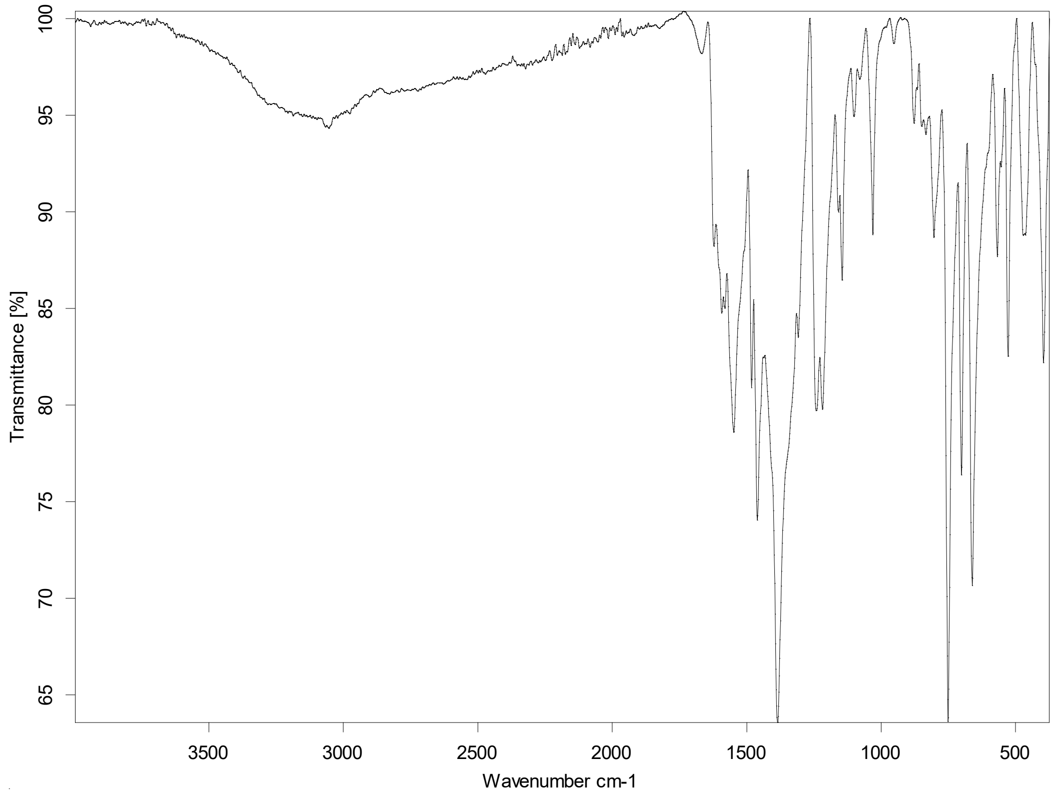

Figure S28. Infrared spectrum of $Tb(Sal)_3{\bullet}H_2O$.

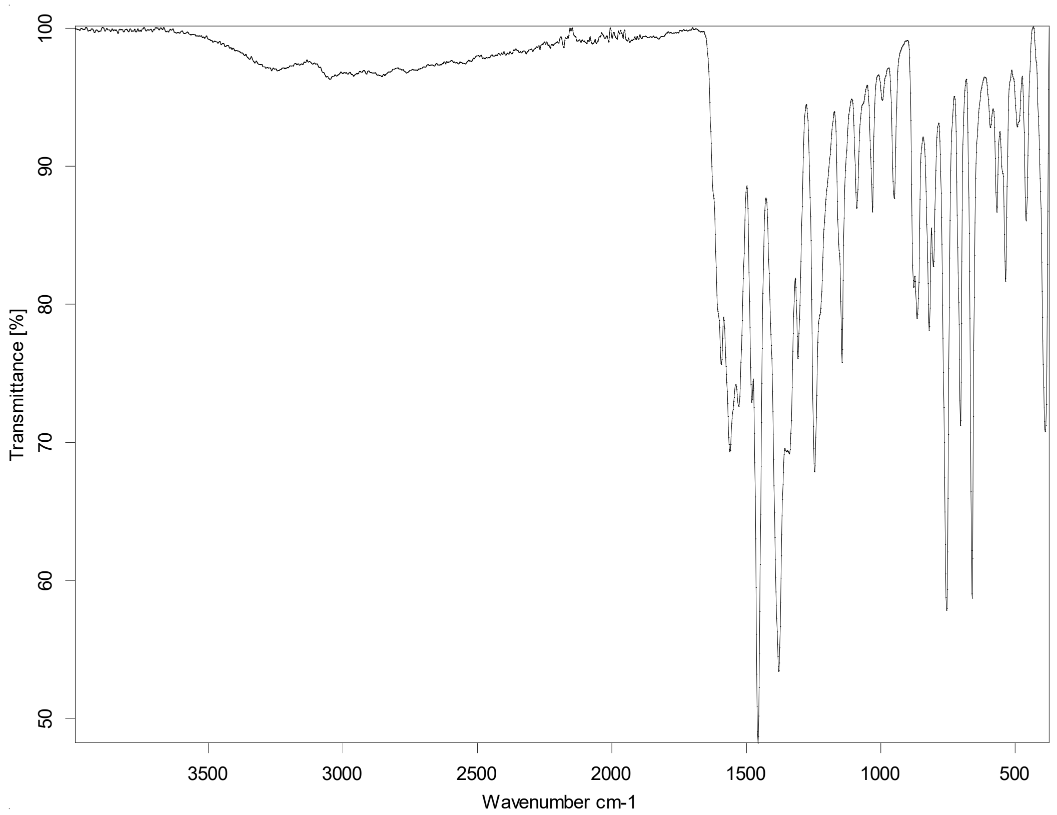

Figure S29. Infrared spectrum of [Chol][La(Sal)$_4$].

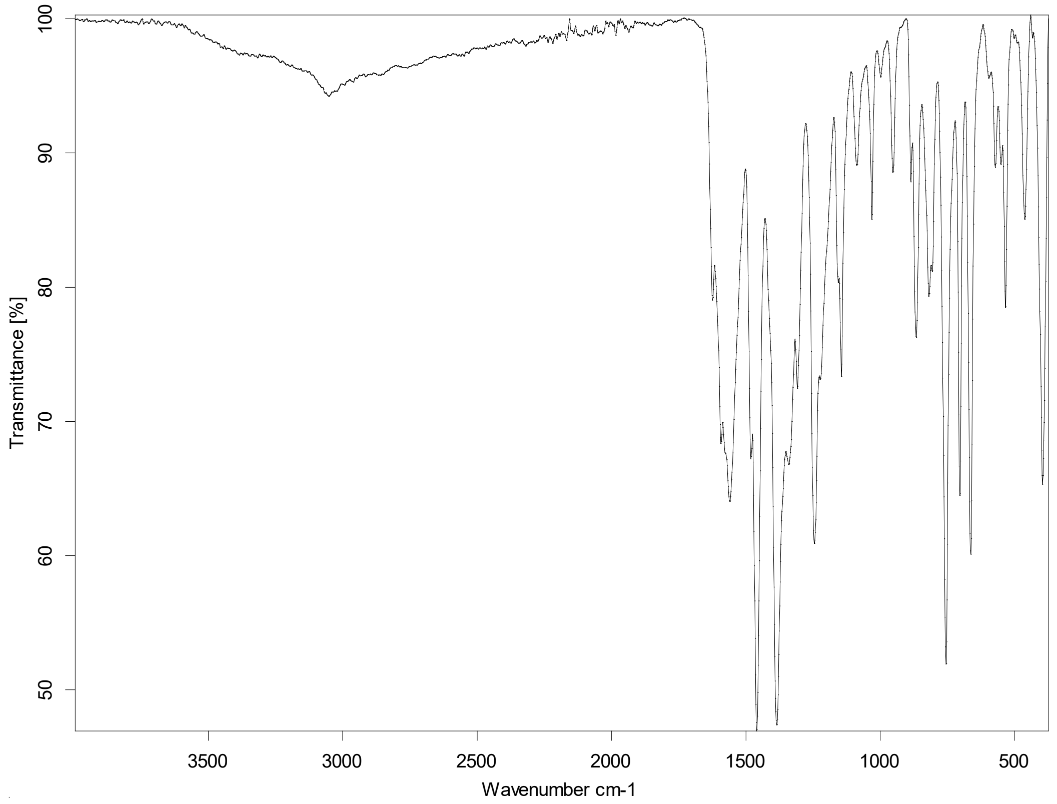

Figure S30. Infrared spectrum of [Chol][Tb(Sal)$_4$].

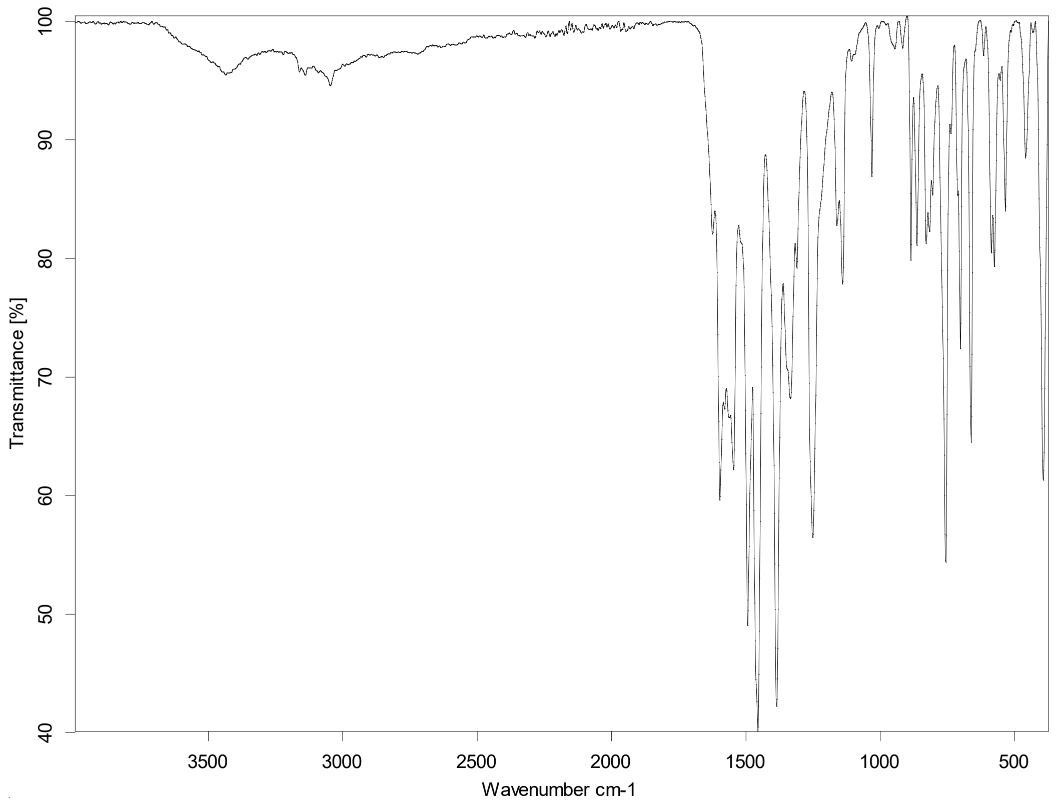

Figure S31. Infrared spectrum of $[C_2Vim][La(Sal)_4]$.

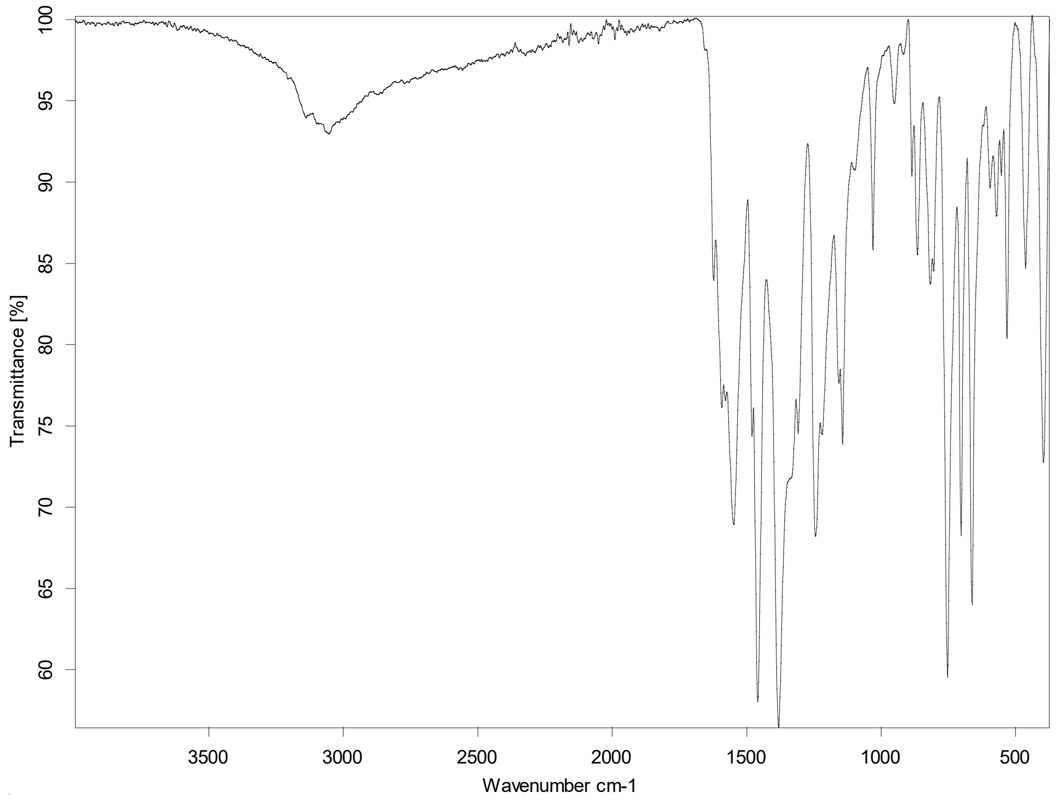

Figure S32. Infrared spectrum of $[C_2Vim][Tb(Sal)_4]$.

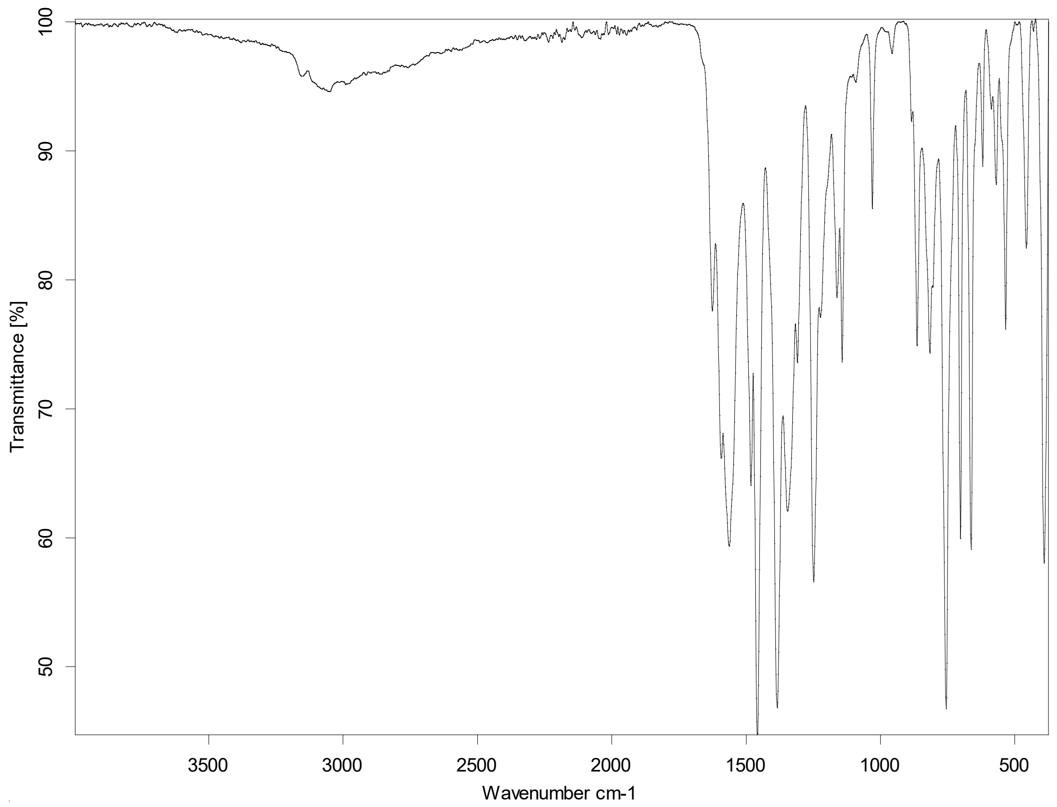

Figure S33. Infrared spectrum of $[C_2C_1im][La(Sal)_4]$.

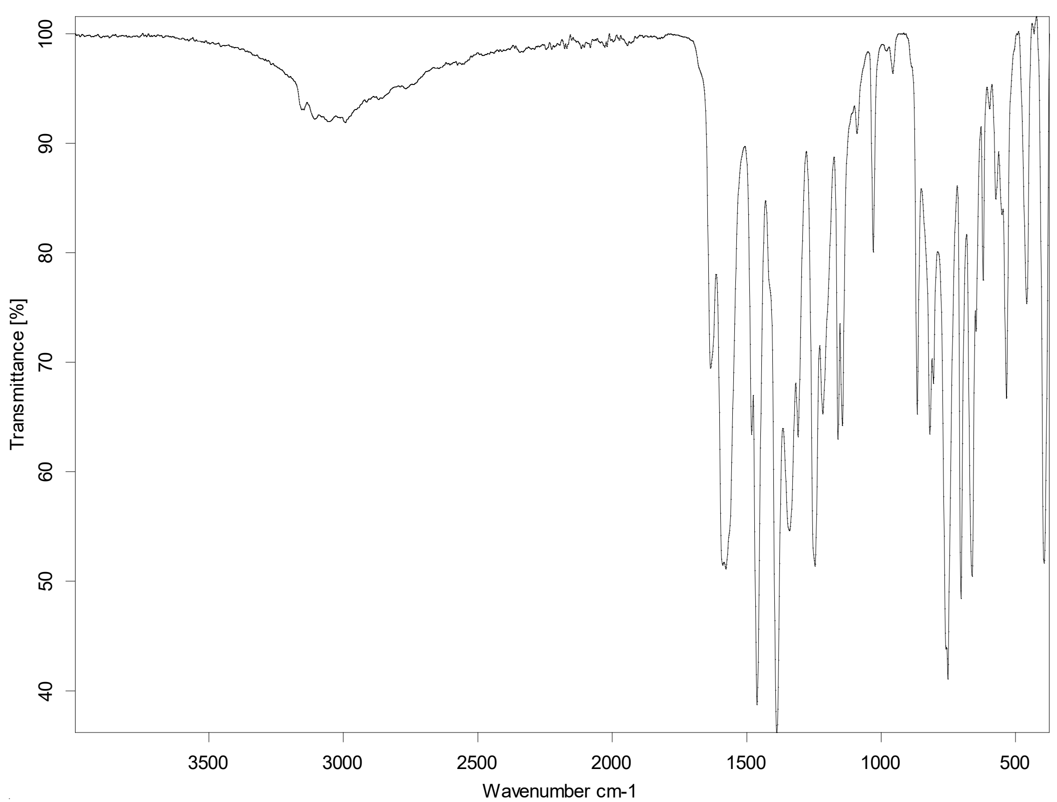

Figure S34. Infrared spectrum of $[C_2C_1im][Tb(Sal)_4]$.

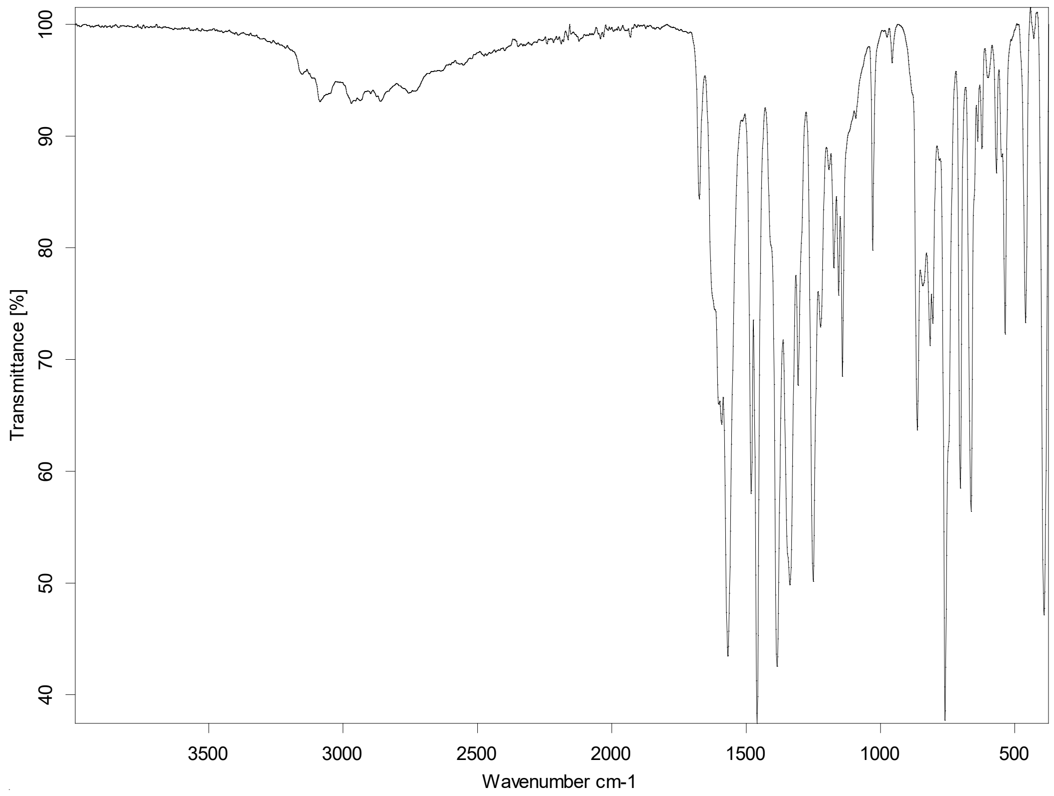

Figure S35. Infrared spectrum of $[C_4C_1im][La(Sal)_4]$.

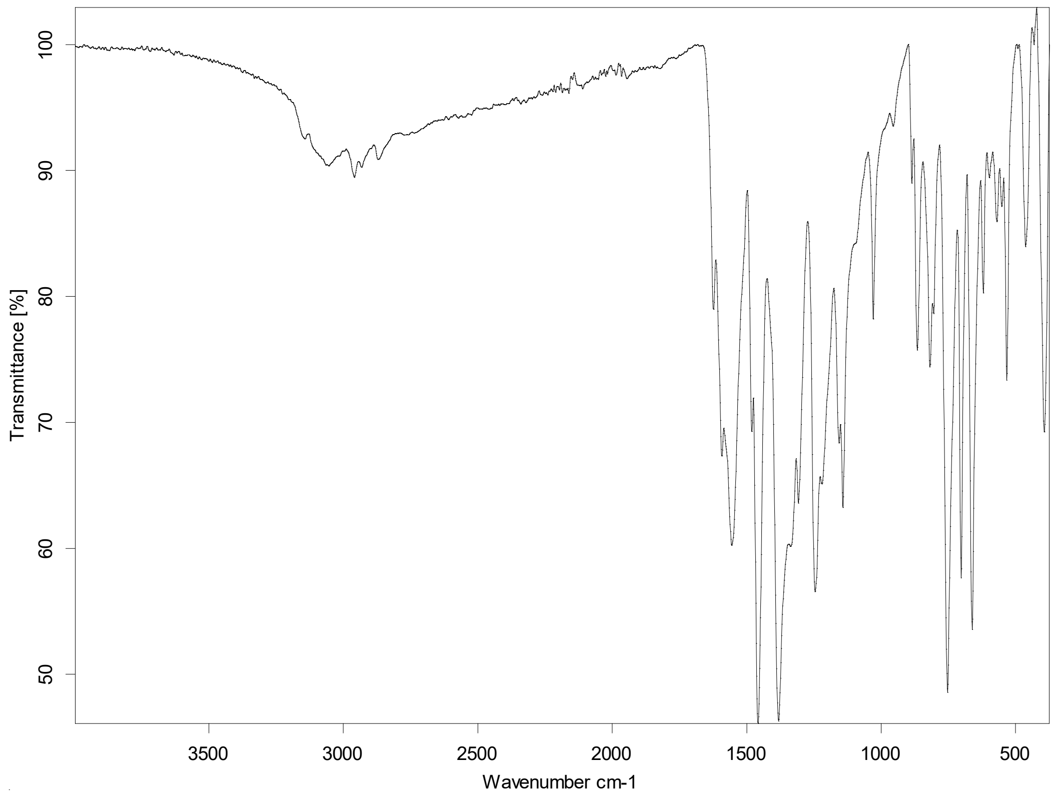

Figure S36. Infrared spectrum of $[C_4C_1im][Tb(Sal)_4]$.

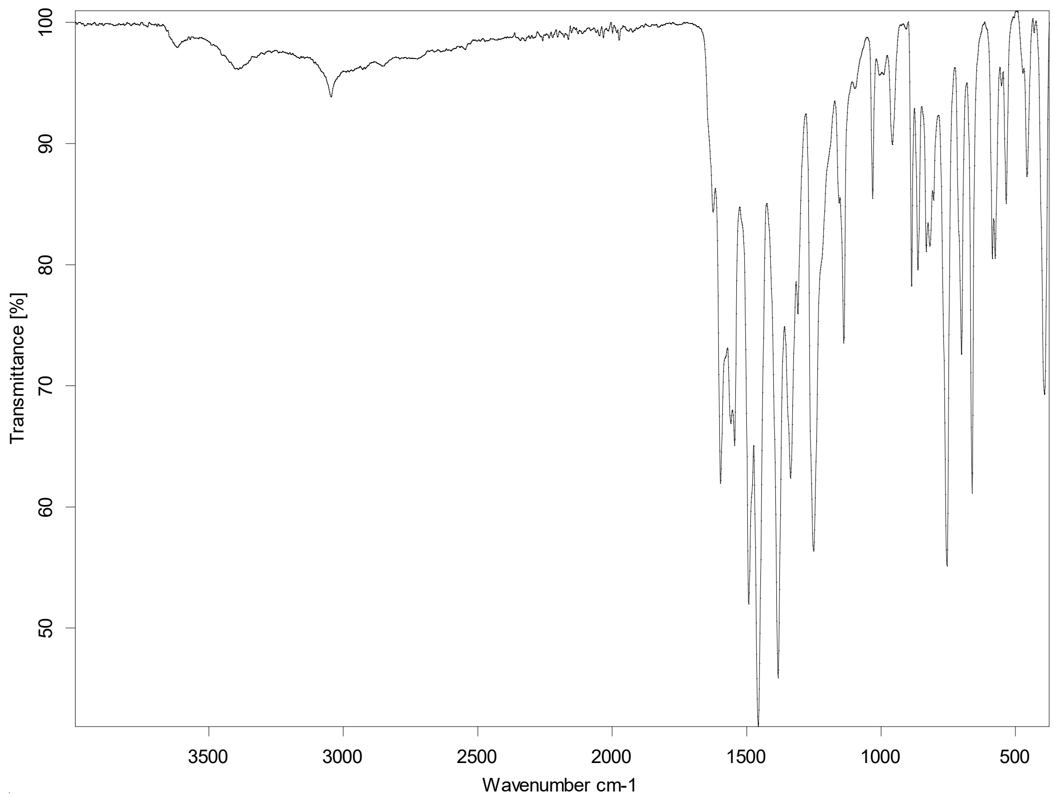

Figure S37. Infrared spectrum of [DADMA][La(Sal)$_4$].

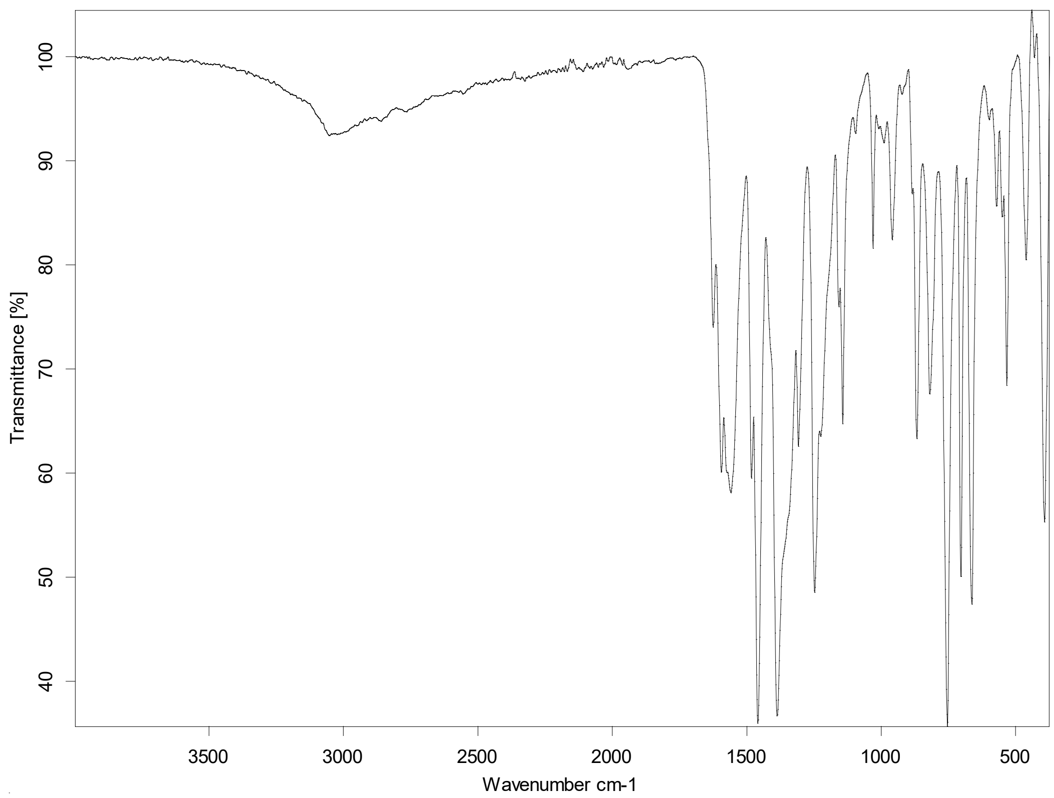

Figure S38. Infrared spectrum of [DADMA][Tb(Sal)$_4$].

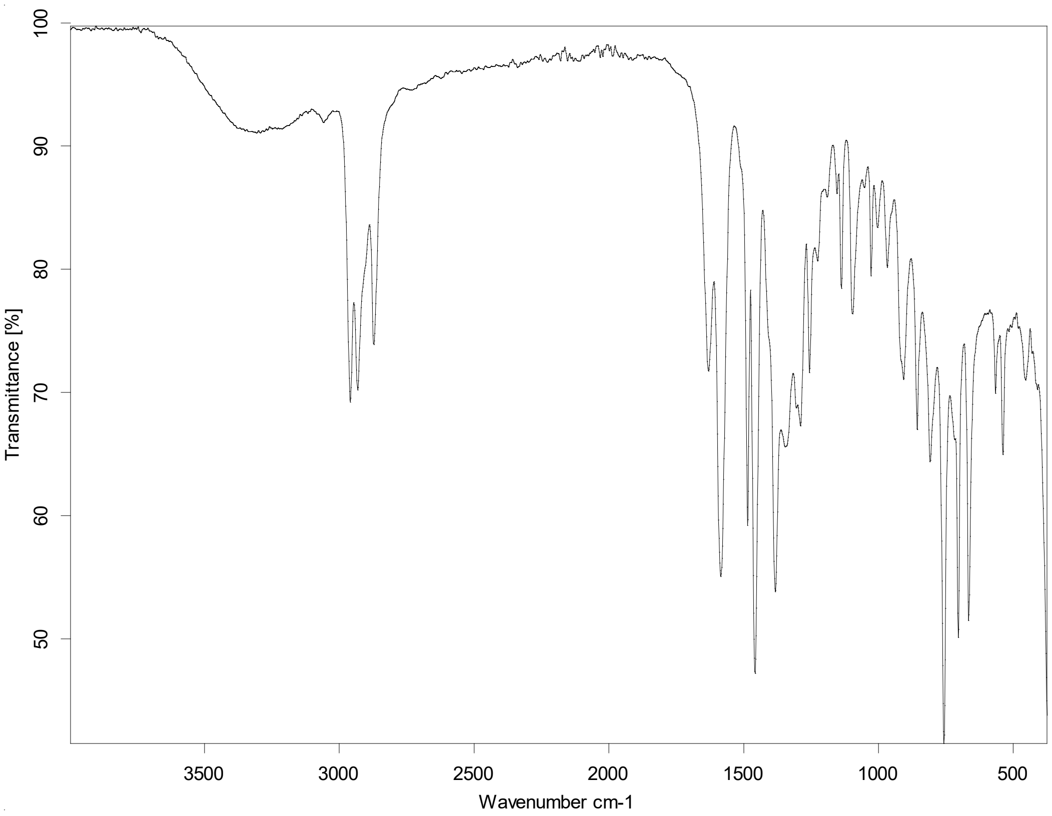

Figure S39. Infrared spectrum of $[P_{4444}][La(Sal)_4]$.

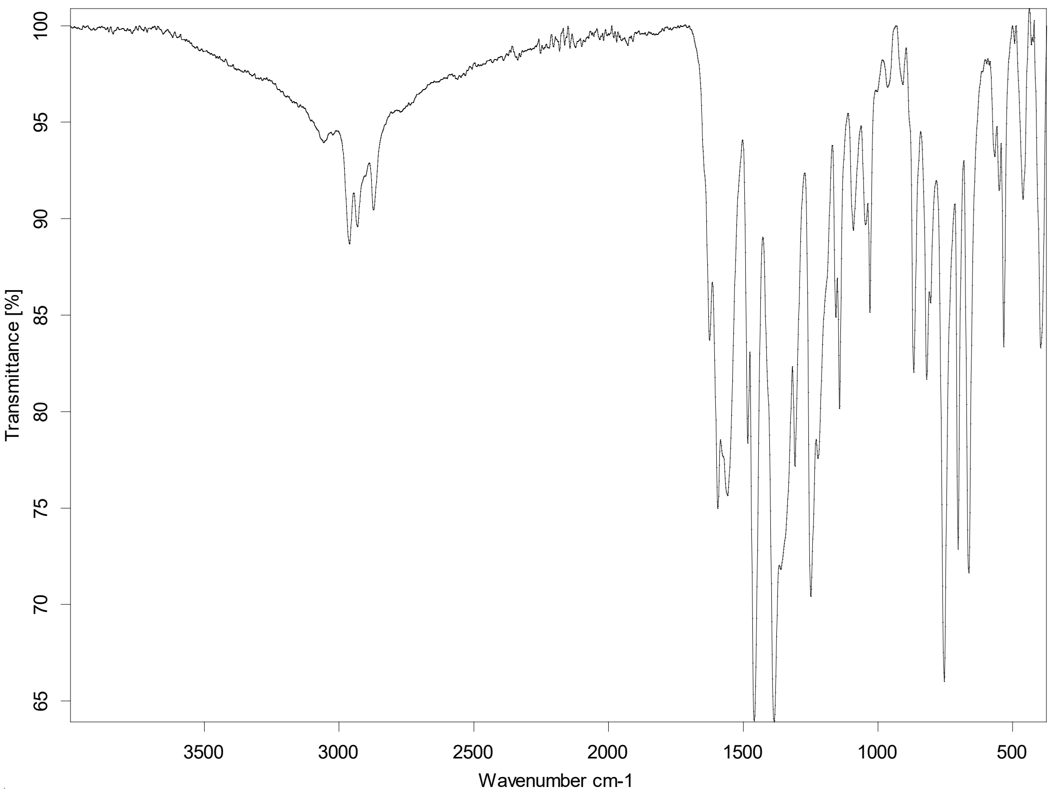

Figure S40. Infrared spectrum of $[P_{4444}][Tb(Sal)_4]$.

V. Mass spectrometry spectra (ESI-MS)

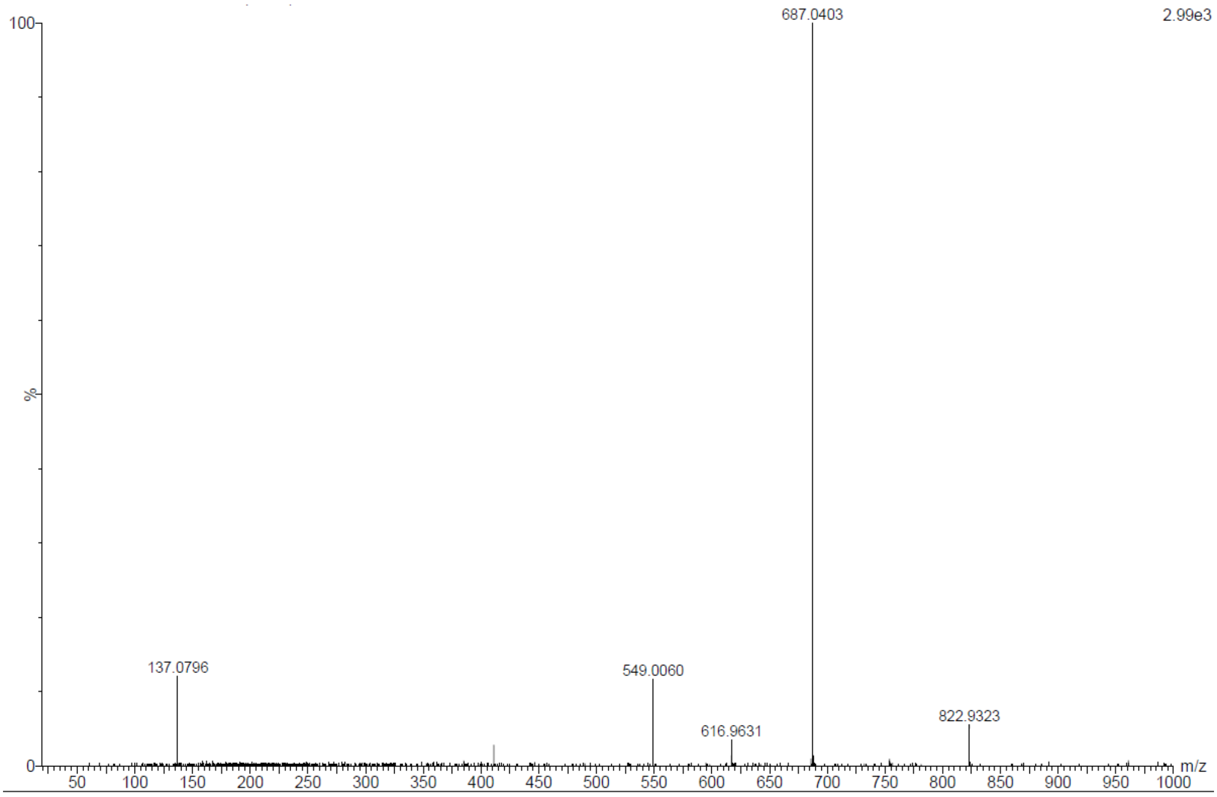

Figure S41. ESI-MS spectrum (negative mode) of $[C_2C_1Im][La(Sal)_4]$.

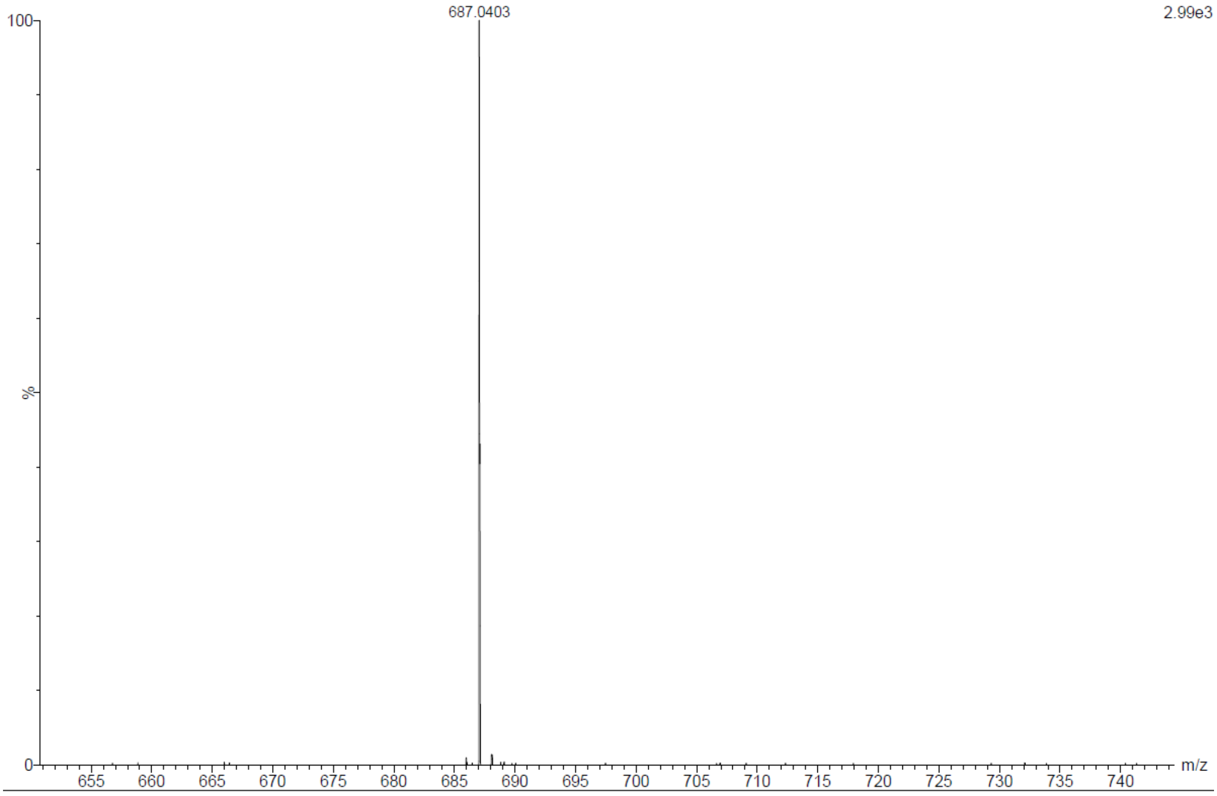

Figure S42. ESI-MS spectrum (negative mode) of $[C_2C_1Im][La(Sal)_4]$ (magnified section).

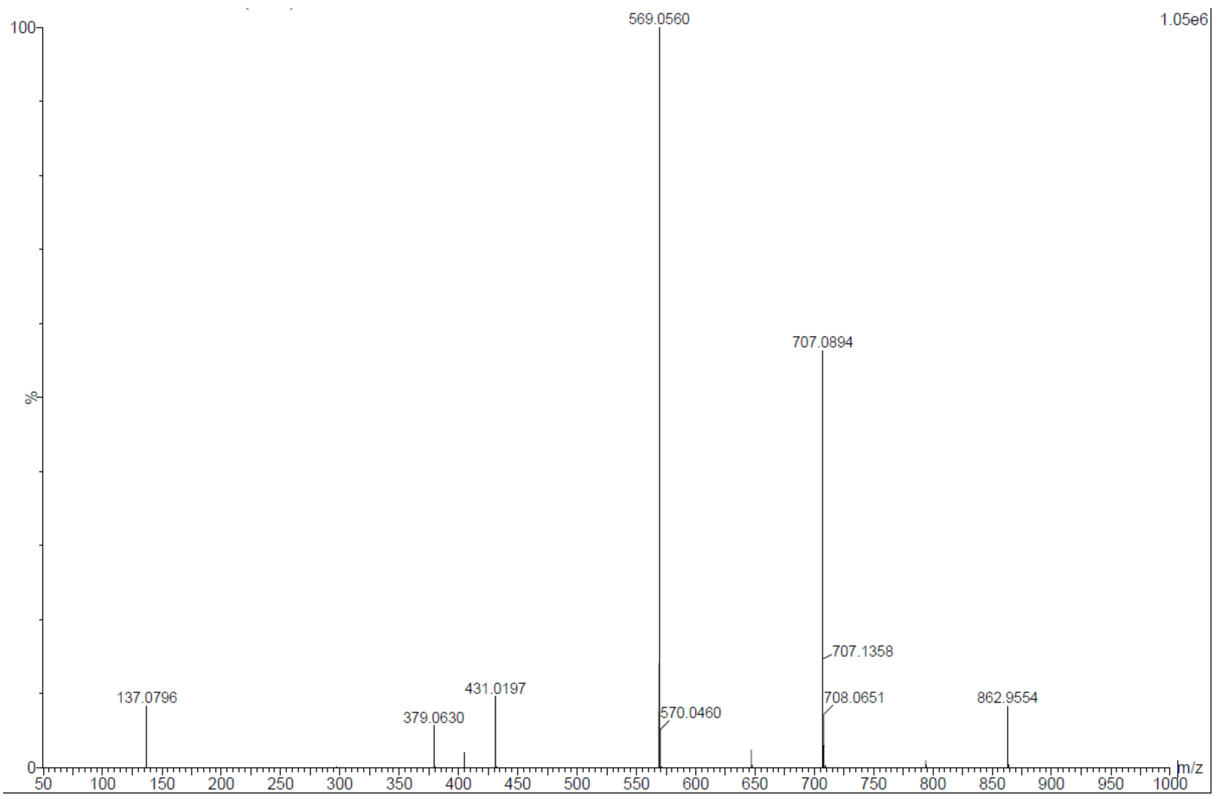

Figure S43. ESI-MS spectrum (negative mode) of [C_2C_1Im][Tb(Sal)$_4$].

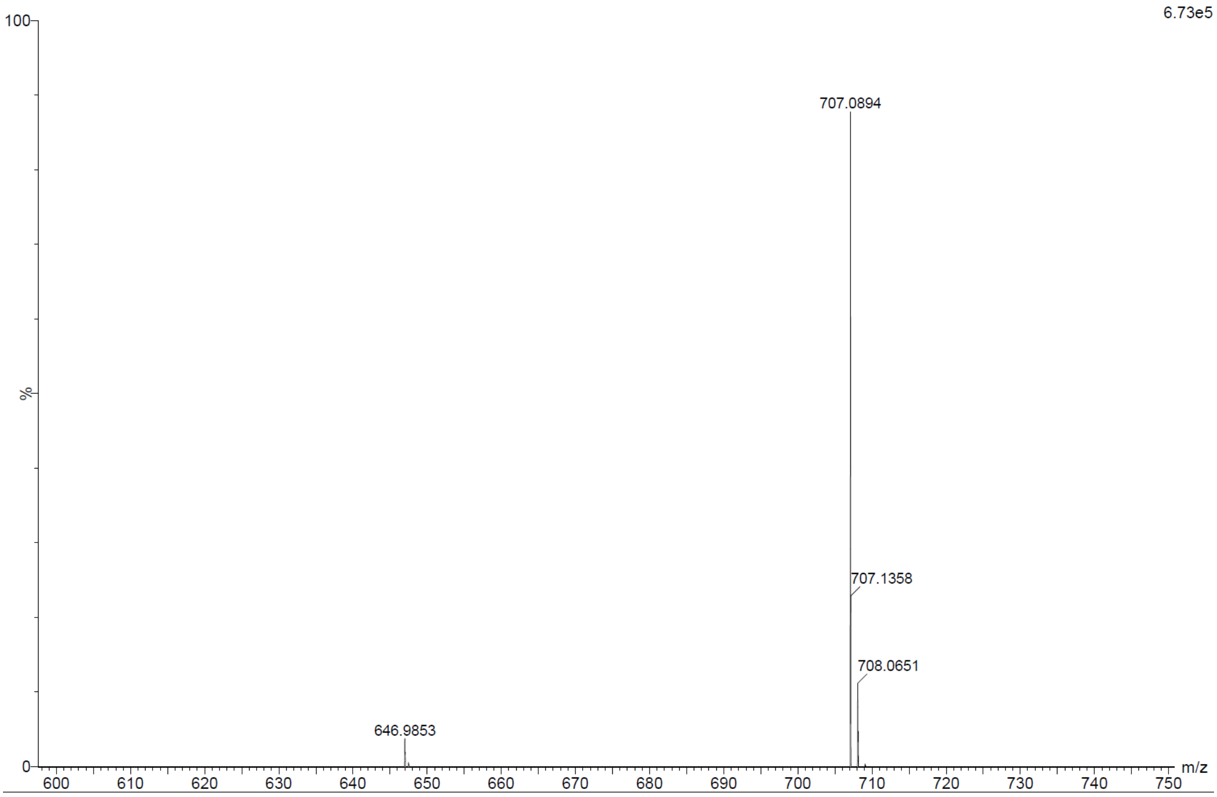

Figure S44. ESI-MS spectrum (negative mode) of [C_2C_1Im][Tb(Sal)$_4$] (magnified section) .

VI. Thermal analyses

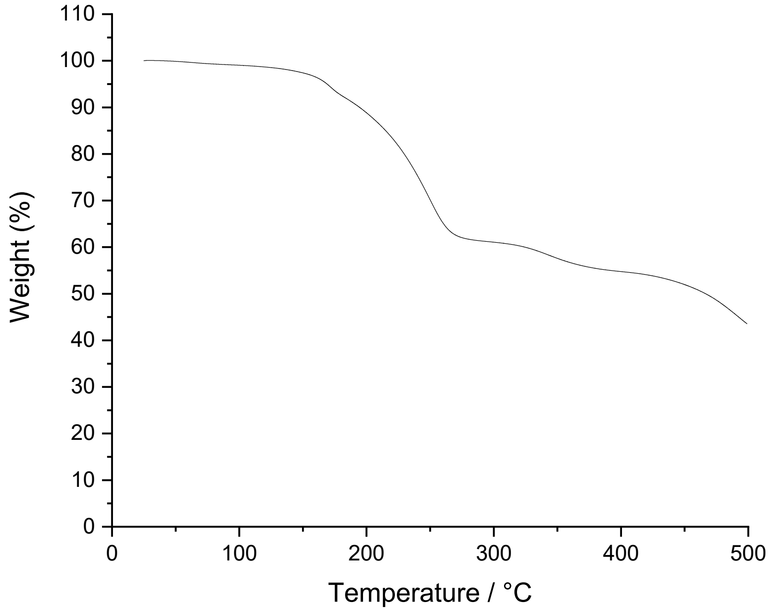

Figure S45. TGA thermogram of La(Sal)$_3$•H_2O.

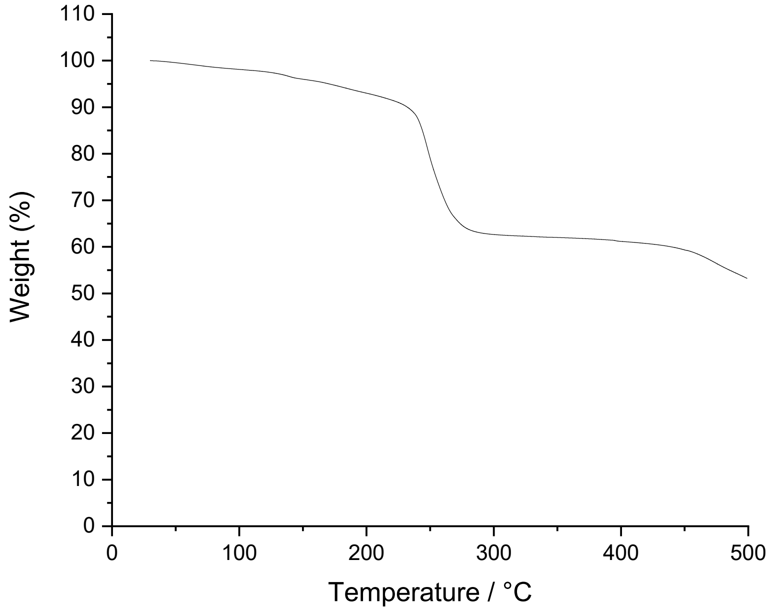

Figure S46. TGA thermogram of Tb(Sal)$_3$•H_2O.

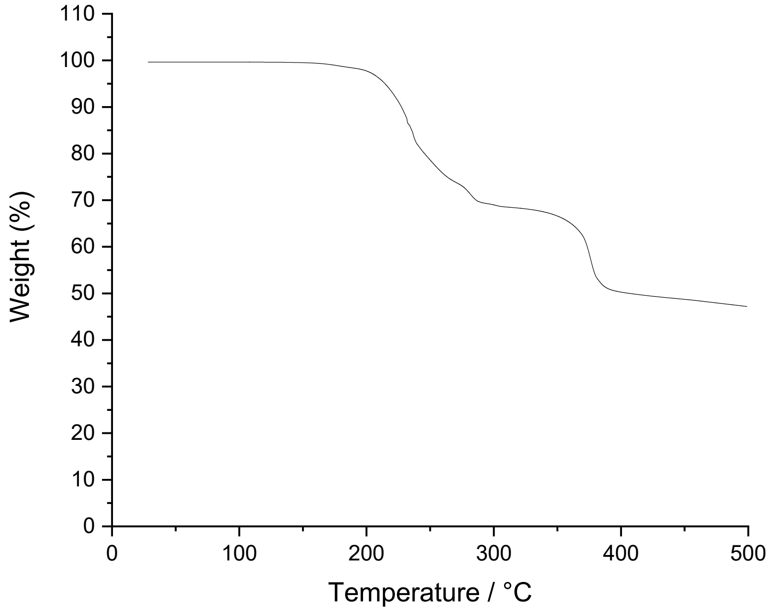

Figure S47. TGA thermogram of $[C_2C_1Im][La(Sal)_4]$.

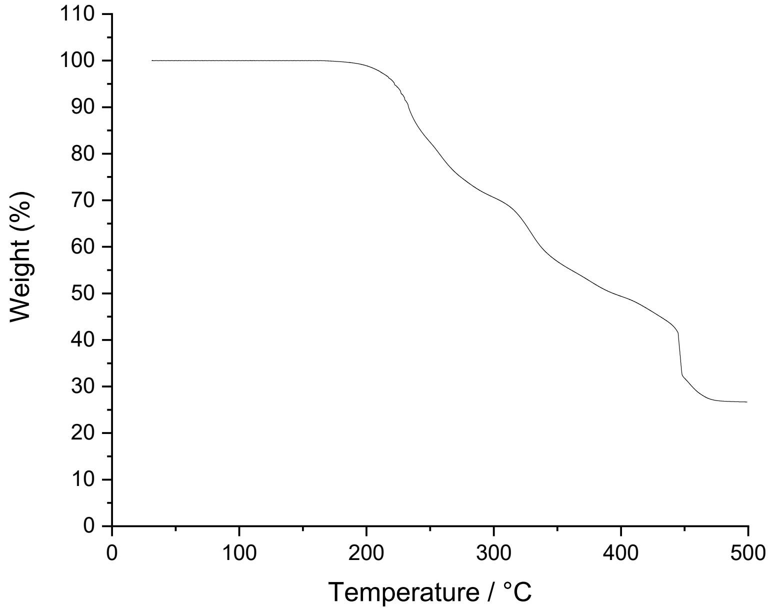

Figure S48. TGA thermogram of $[C_2C_1Im][Tb(Sal)_4]$.

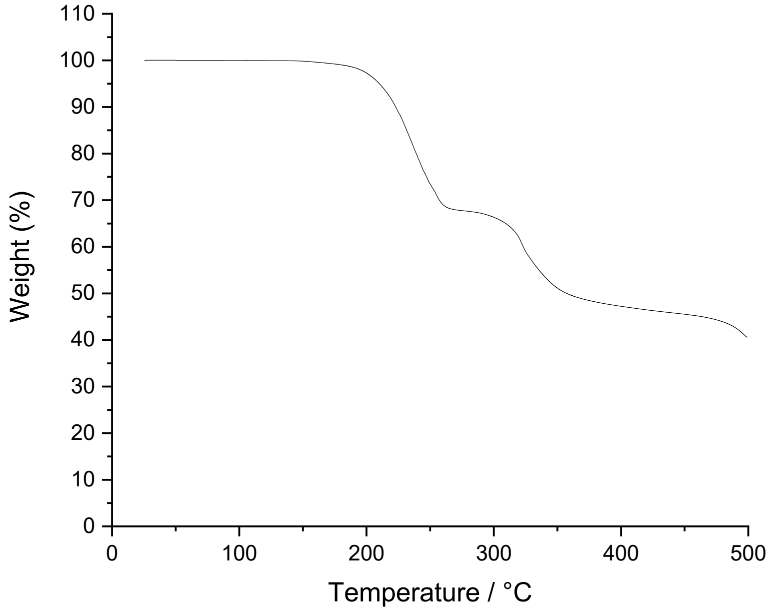

Figure S49. TGA thermogram of $[C_4C_1Im][La(Sal)_4]$.

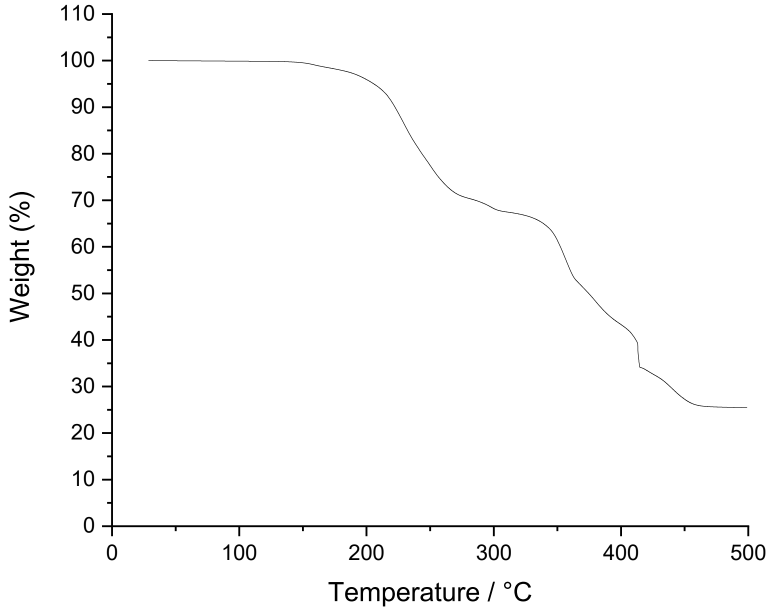

Figure S50. TGA thermogram of compound $[C_4C_1Im][Tb(Sal)_4]$.

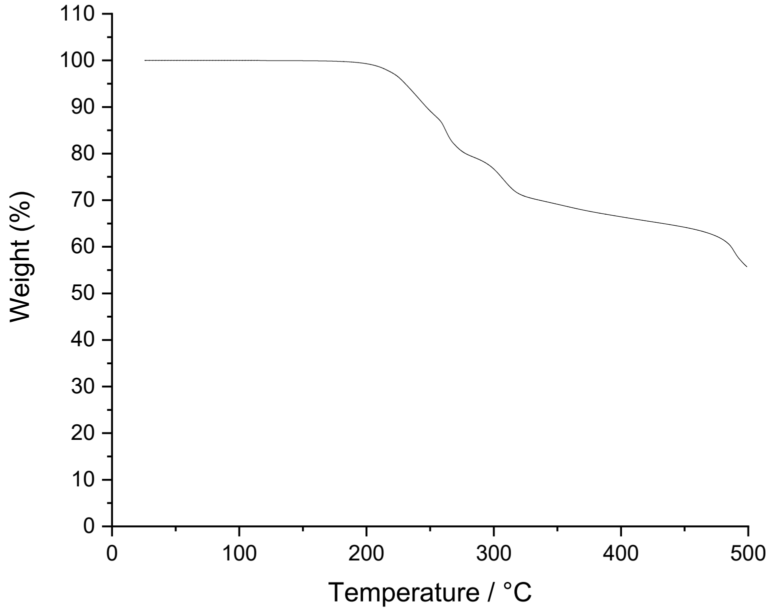

Figure S51. TGA thermogram of $[C_2Vim][La(Sal)_4]$.

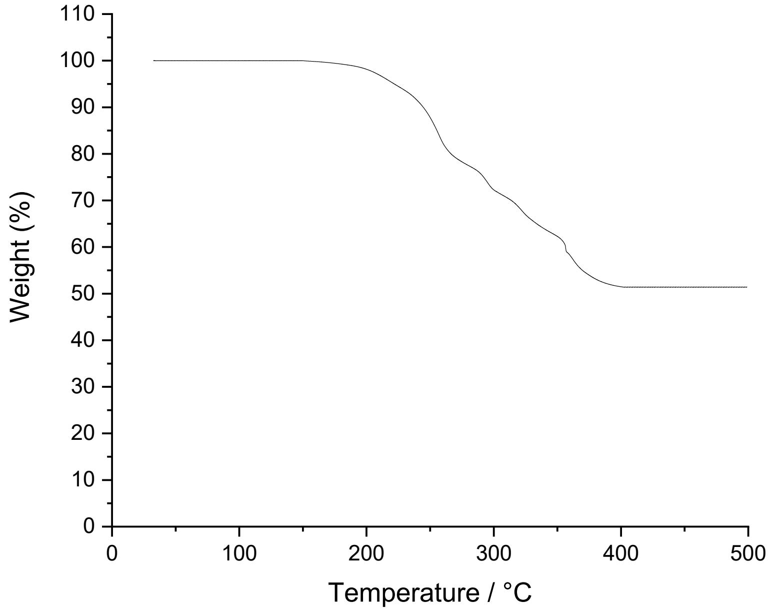

Figure S52. TGA thermogram of $[C_2Vim][Tb(Sal)_4]$.

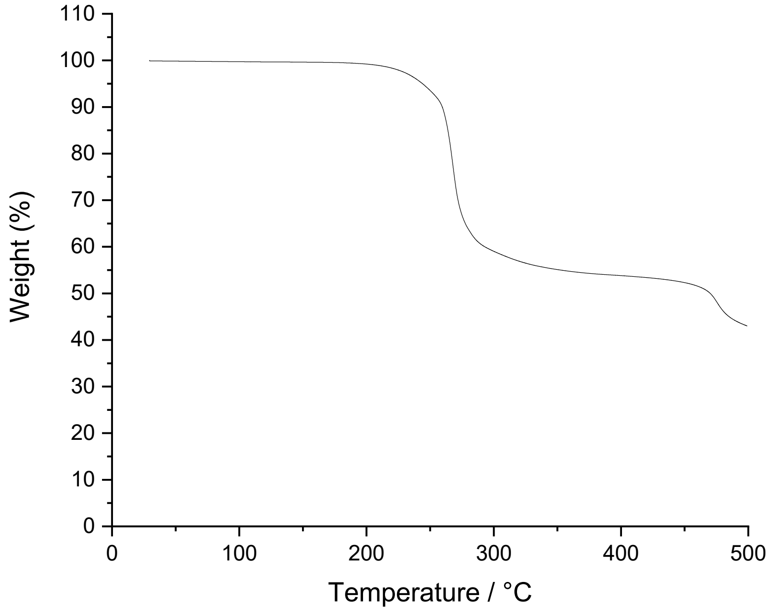

Figure S53. TGA thermogram of [Chol][La(Sal)$_4$].

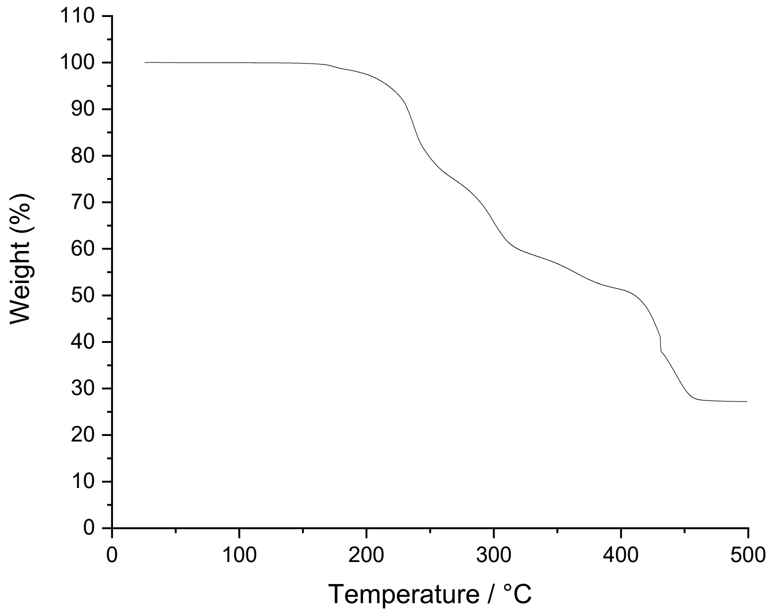

Figure S54. TGA thermogram of [Chol][Tb(Sal)$_4$].

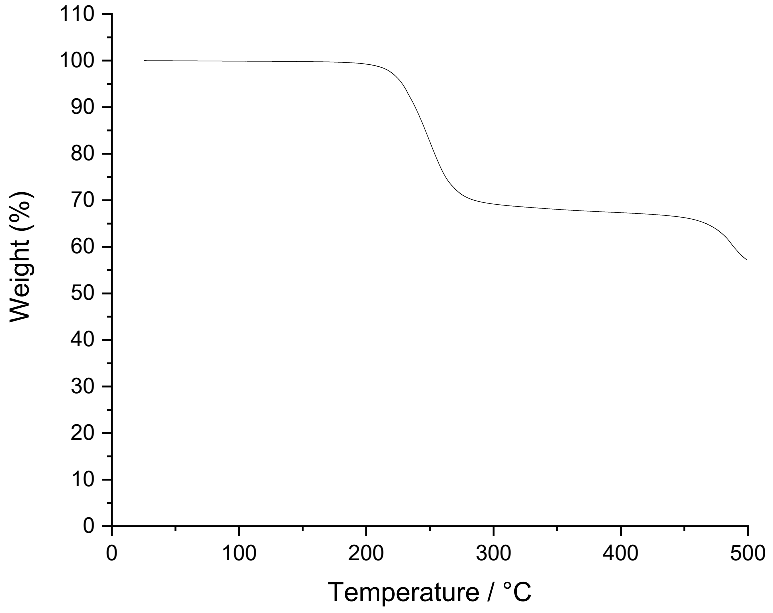

Figure S55. TGA thermogram of [DADMA][La(Sal)$_4$].

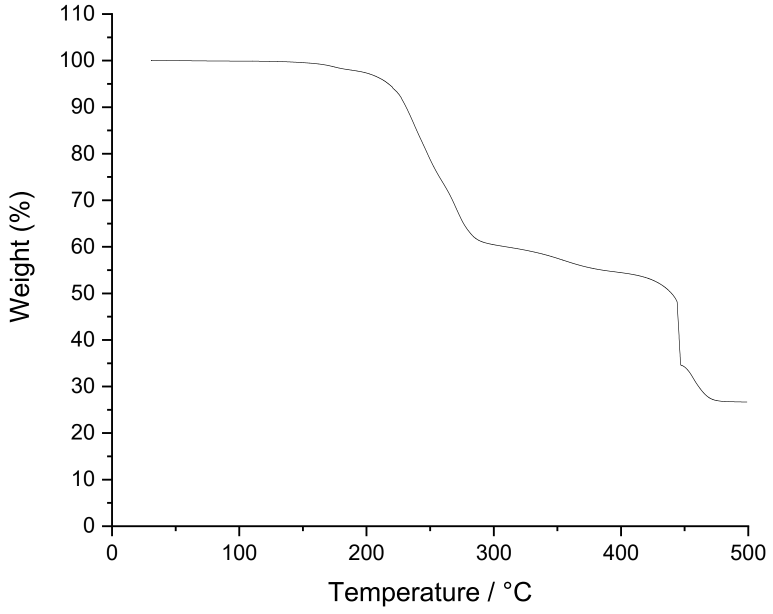

Figure S56. TGA thermogram of [DADMA][Tb(Sal)$_4$].

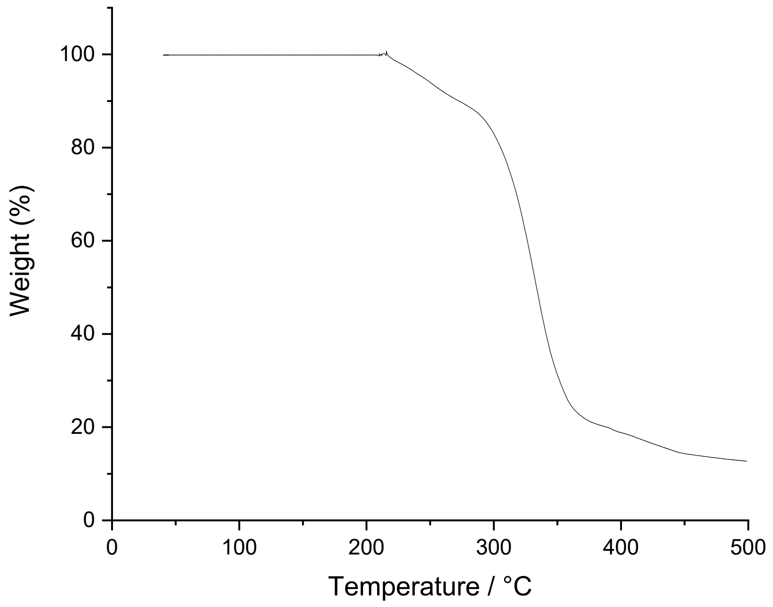

Figure S57. TGA thermogram of $[P_{4444}][La(Sal)_4]$.

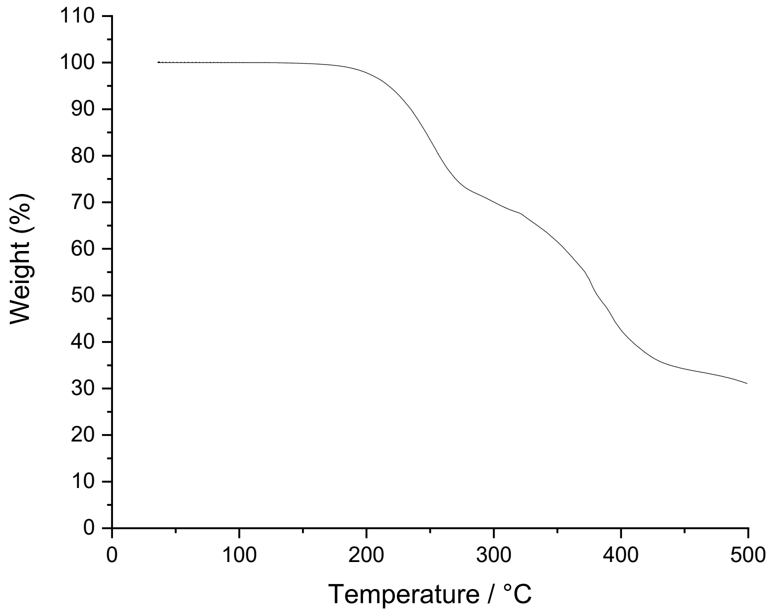

Figure S58. TGA thermogram of $[P_{4444}][Tb(Sal)_4]$.

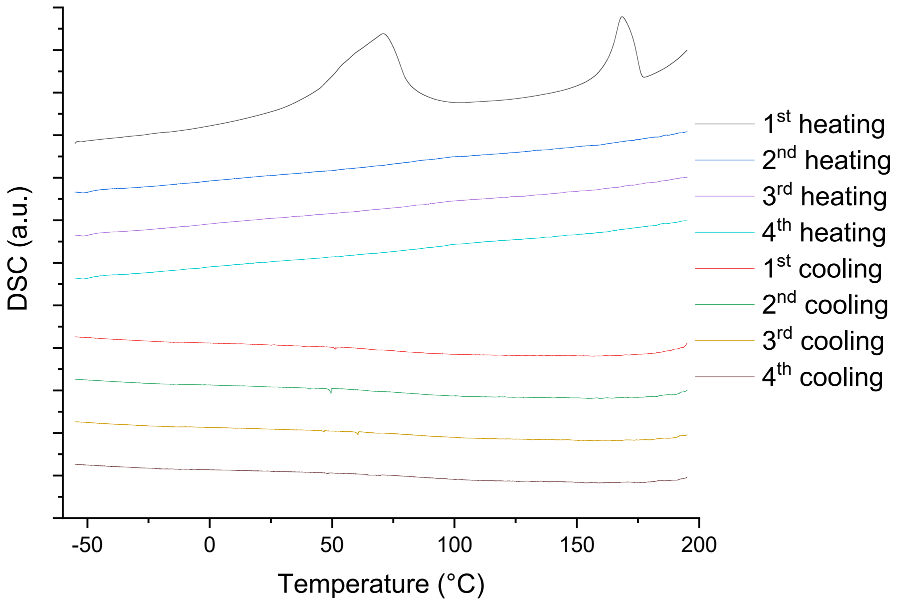

Figure S59. DSC traces of [P_{4444}][Tb(Sal)$_4$] (5 K/min).

VII. Polarized Optical Microscopy (POM) micrographs

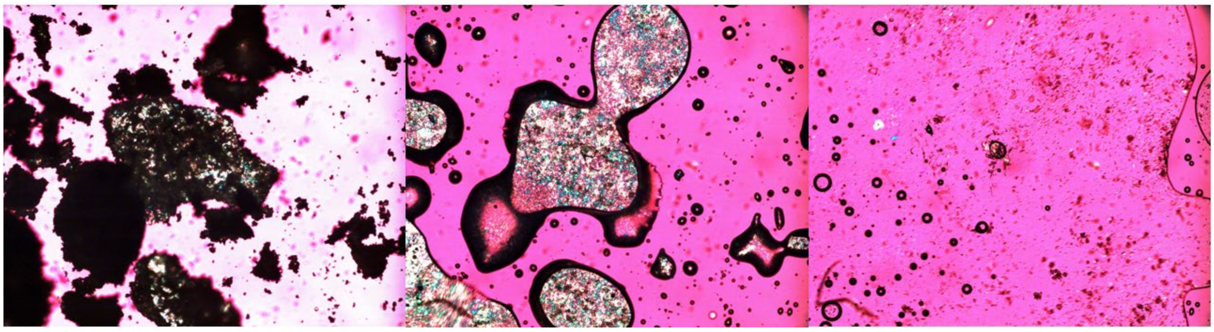

(a) (b) (c)

Figure S60. POM micrograph of $[P_{4444}][Tb(Sal)_4]$ (a): 100.5 °C, (b): 168.0 °C, (c) 170.5 °C.

VIII. UV-Vis spectroscopy

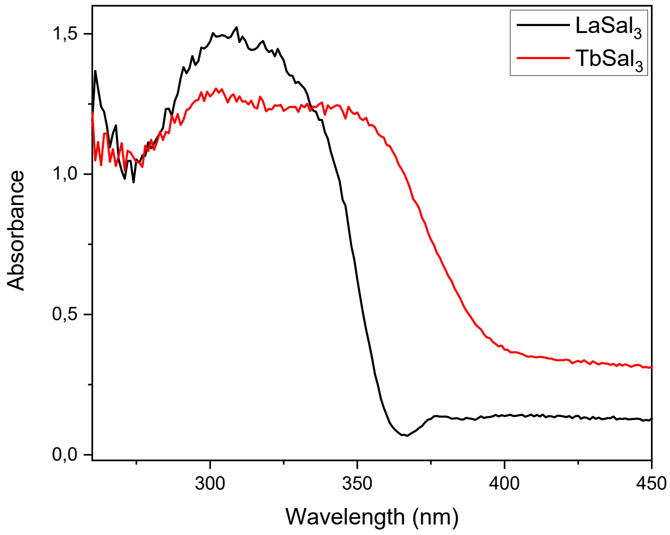

Figure S61. Absorption spectrum of La(Sal)$_3$•H_2O and Tb(Sal)$_3$•H_2O.

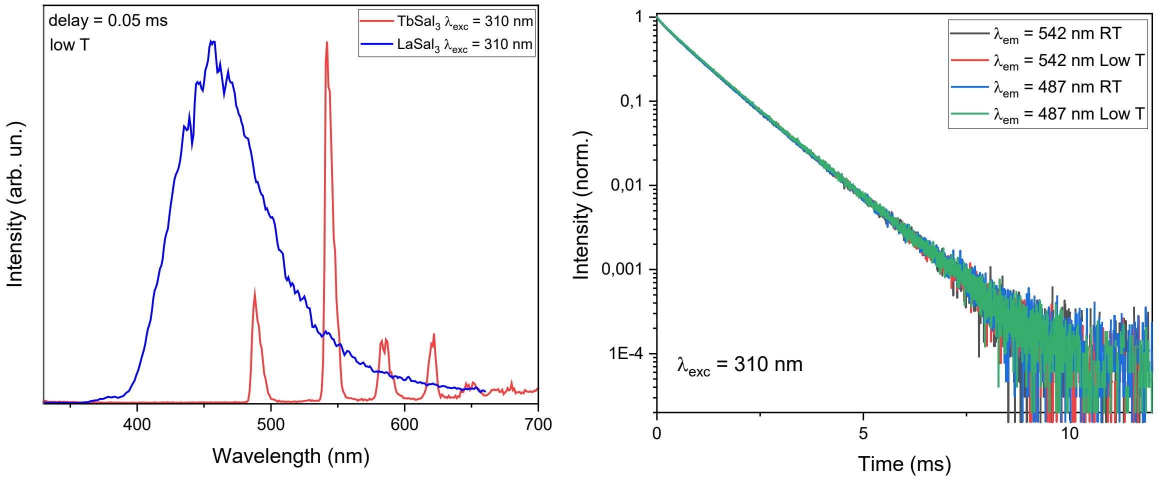

Figure S62. (*Left*) phosphorescence spectrum of LaSal$_3$ (blue line) and of TbSal$_3$ (red line). The spectra were acquired at low T (N_2) with a delay of 0.05 ms. (*Right*) decays corresponding to the $^5D_4 \rightarrow {}^7F_6$ and $^5D_4 \rightarrow {}^7F_5$ transitions of TbSal$_3$ upon excitation in ligand level at 310 nm, both at RT and low T.

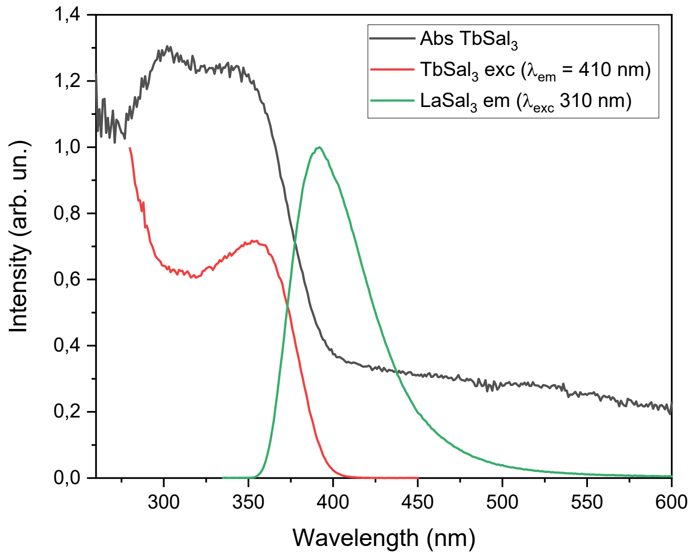

Figure S63. Overlay of absorption and excitation spectra of $Tb(Sal)_3•H_2O$ and emission spectrum of $La(Sal)_3•H_2O$ at RT.

Table S2. CIE coordinates for La-compounds (*top*) and Tb-compounds (*bottom*) calculated from emission spectra collected under excitation with λ_{exc} = 310 nm.

	x	*y*
$La(Sal)_3•H_2O$	0.15	0.10
$[C_2C_1Im][La(Sal)_4]$	0.16	0.06
$[C_4C_1Im][La(Sal)_4]$	0.15	0.12
$[C_2Vim][La(Sal)_4]$	0.16	0.06
$[DADMA][La(Sal)_4]$	0.17	0.13
$[Chol][La(Sal)_4]$	0.17	0.13
$[P_{4444}][La(Sal)_4]$	0.16	0.06

	x	*y*
$Tb(Sal)_3•H_2O$	0.33	0.56
$[C_2C_1Im][Tb(Sal)_4]$	0.35	0.57
$[C_4C_1Im][Tb(Sal)_4]$	0.34	0.56
$[C_2Vim][Tb(Sal)_4]$	0.35	0.58
$[DADMA][Tb(Sal)_4]$	0.35	0.58
$[Chol][Tb(Sal)_4]$	0.35	0.57
$[P_{4444}][Tb(Sal)_4]$	0.37	0.57

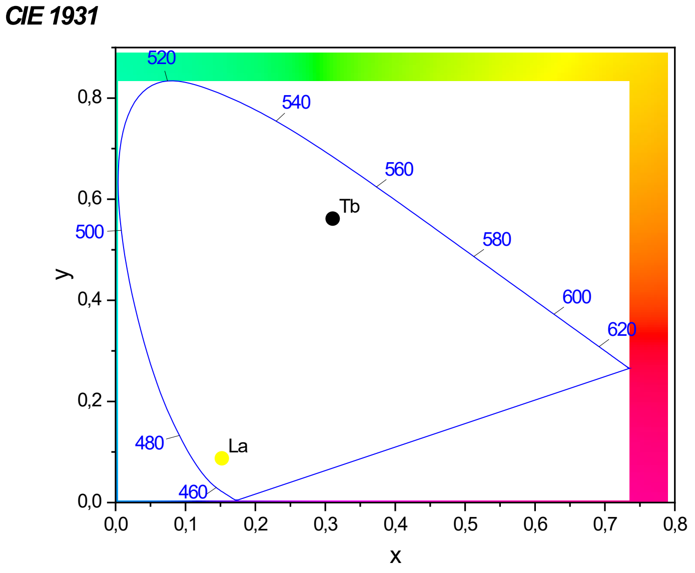

Figure S64. CIE coordinate areas for La-compounds (yellow circle) and for Tb-compounds (black circle) obtained from emission spectra at 310 nm excitation.

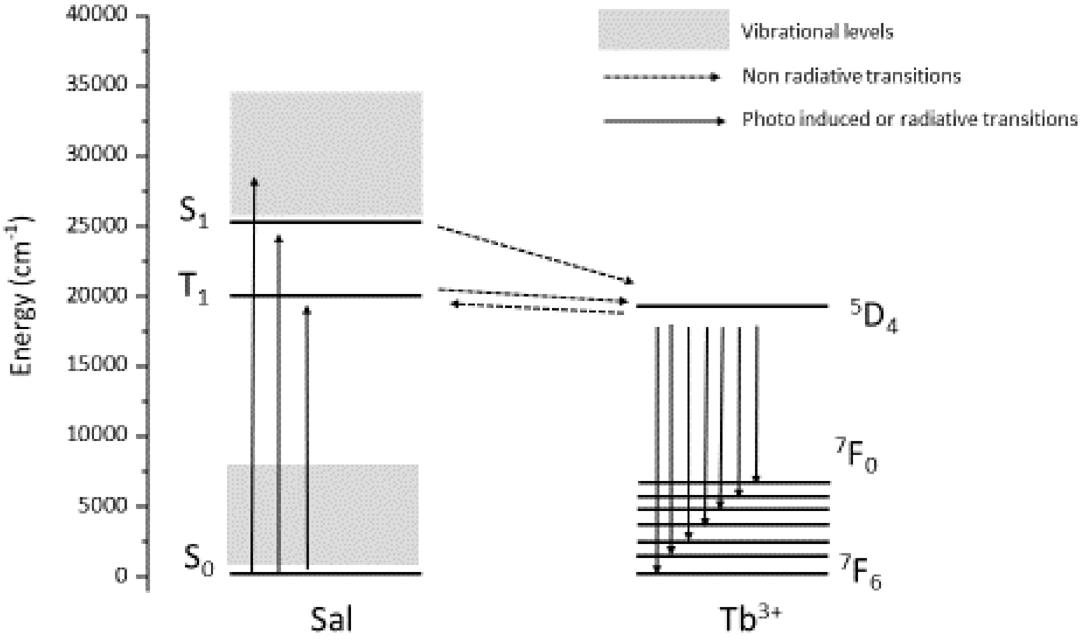

Figure S65. Proposed energy transfer mechanism for the terbium containing samples.

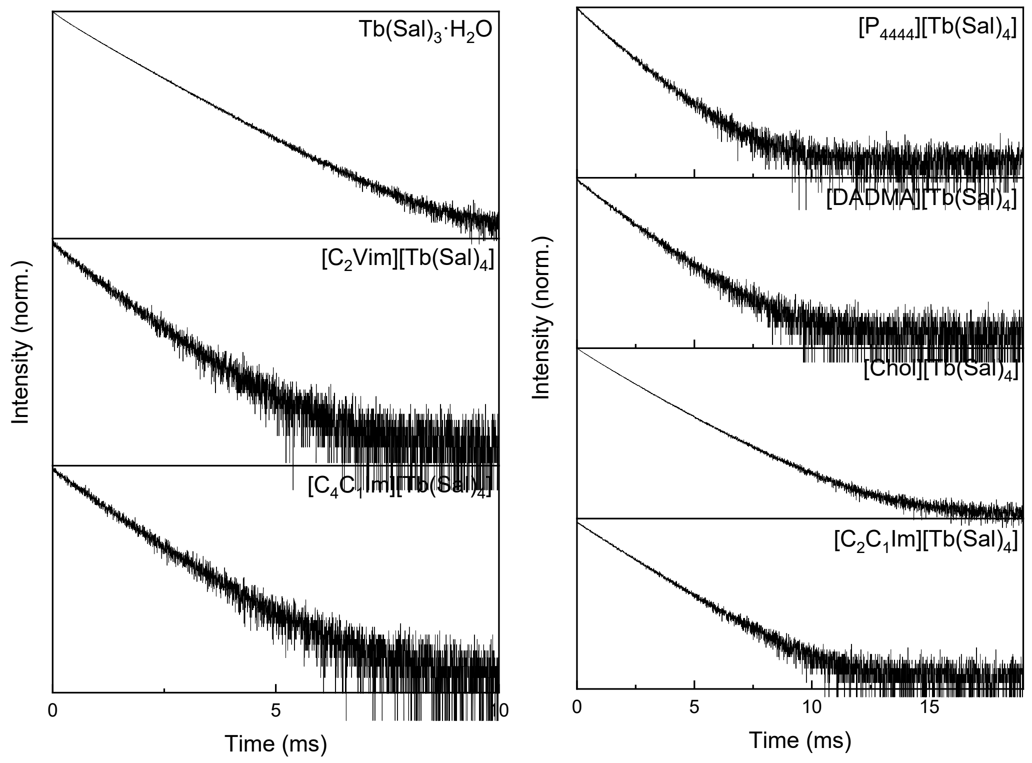

Figure S66. Lifetime decays curves for all the Tb-based compounds, acquired by exciting at 310 nm and detecting the emission at 542 nm.

IX. Reference

(1) Burns, J. H.; Baldwin, W. H. Crystal Structures of Aquotris(Salicylato)Samarium(III) and Aquotris(Salicylato)Americium(III). *Inorg. Chem.* **1977**, *16* (2), 289–294.
